# New Paracyclophanylthiazoles with Anti-Leukemia Activity: Design, Synthesis, Molecular Docking, and Mechanistic Studies

**DOI:** 10.3390/molecules25133089

**Published:** 2020-07-07

**Authors:** Ashraf A Aly, Stefan Bräse, Alaa A. Hassan, Nasr K. Mohamed, Lamiaa E. Abd El-Haleem, Martin Nieger, Nesrin M. Morsy, Elshimaa M. N. Abdelhafez

**Affiliations:** 1 Chemistry Department, Faculty of Science, Minia University, 61519-El-Minia, Egypt; alaahassan2001@mu.edu.eg (A.A.H.); nasrmohamed603@yahoo.com (N.K.M.); lamiaaelsayed2013@yahoo.com (L.E.A.E.-H.); 2 Institute of Organic Chemistry, Karlsruhe Institute of Technology, 76131 Karlsruhe, Germany; 3 Institute of Biological and Chemical Systems–Functional Molecular Systems (IBCS-FMS), Karlsruhe Institute of Technology, Hermann-von-Helmholtz-Platz 1, D-76344 Eggenstein-Leopoldshafen, Germany; 4 Department of Chemistry, University of Helsinki, P.O. Box 55 (A. I. Virtasen aukio I), 00014 Helsinki, Finland; martin.nieger@helsinki.fi; 5 Organometallic and Organometalloid Chemistry Department, National Research Centre, Dokki, 12622 Cairo, Egypt; nesrinmorsy@yahoo.com; 6 Department of Medicinal Chemistry, Faculty of Pharmacy, Minia University, 61519-El Minia, Egypt; shimaanaguib_80@yahoo.com

**Keywords:** paracyclophanes, NMR, X-ray, NCI-60, cancer cell lines, mechanism, docking

## Abstract

A new series of methyl 2-(2-(4′-[2.2]paracyclophanyl)-hydrazinylidene)-3-substituted-4-oxothiazolidin-5-ylidene)acetates **3a**–**f** were synthesized from the reaction of paracyclophanyl-acylthiosemicarbazides **2a**–**f** with dimethyl acetylenedicarboxylate. Based upon nuclear magnetic resonance (NMR), infrared (IR), and mass spectra (HRMS), the structure of the obtained products was elucidated. X-ray structure analysis was also used as unambiguous tool to elucidate the structure of the products. The target compounds **3a**–**f** were screened against 60 cancer cell lines. They displayed anticancer activity against a leukemia subpanel, namely, RPMI-8226 and SR cell lines. The activity of compound **3a** was found as the most cytotoxic potency against 60 cancer cell lines. Consequently, it was selected for further five doses analysis according to National Cancer Institute (NCI) protocol. The cytotoxic effect showed selectivity ratios ranging between 0.63 and 1.28 and between 0.58 and 5.89 at the GI_50_ and total growth inhibition (TGI) levels, respectively. Accordingly, compound **3a** underwent further mechanistic study against the most sensitive leukemia RPMI-8226 and SR cell lines. It showed antiproliferation with IC50 = 1.61 ± 0.04 and 1.11 ± 0.03 µM against RPMI-8226 and SR cell lines, respectively. It also revealed a remarkable tubulin inhibitory activity, compared to colchicine with IC50 = 4.97 µM/mL. Caspase-3, BAX, and Bcl-2 assays for **3a** using annexin V-FITC staining revealed significant pro-apoptotic activity. Furthermore, multidrug-resistant leukemia SR cells were used to show better resistance indices (1.285 ng/mL, 1.15-fold) than the reference. Docking studies with β-tubulin indicate that most of the tested compounds illustrated good binding at the colchicine binding site of the enzyme, especially for compound **3a**, which made several interactions better than that of the reference colchicine.

## Highlights

- Synthesis of methyl 2-(2-(4′-[2.2]paracyclophanyl)-hydrazine-ylidene)-4-oxothiazolidin-5-ylidene)acetates was here reported.
- Cytotoxic activity of the synthesized compounds toward the NCI-60 panel of cancer cell lines was determined, and the cellular mechanism of the most potent inhibitors was further investigated in leukemia cell lines.
- Compound **3a** was found as the most cytotoxic one, and it was selected for further five-dose analysis according to NCI protocol.
- Compound **3a**, in comparison to other derivatives, exhibited high specificity against leukemia RPMI-8226 and SR cell lines, and it also showed a remarkable tubulin inhibitory activity in relation to colchicine with IC_50_ = 4.97 µM/mL.
- Docking studies with β-tubulin revealed that most of the tested compounds showed good binding at the colchicine binding site of the enzyme, especially for compound **3a**, which made several interactions better than that of the reference colchicine.

## 1. Introduction

**Figure 1:**
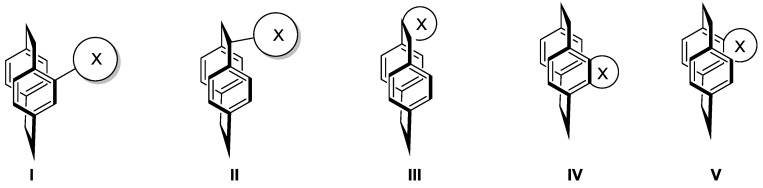
Five types of heterocycle-substituted [2.2]paracyclophanes.

Cyclophane chemistry is rapidly growing in the field of stereoselective synthesis with its incorporation into heterocyclic and/or polymer chemistry [1,2,3,4,5,6,7,8]. A great deal of attention is focused on developing new synthetic tools for synthesizing functionalized [2.2]paracyclophanes. Substituted [2.2]paracyclophanes can also serve as chiral templates and/or as auxiliaries [9]. The synthesis and application of heterocycles based on [2.2]paracyclophane [10,11] can be organized into five structural classes (Figure 1): heterocycle derived by paracyclophanyl group (type **I**), heterocycle derived by bridge (type **II**), heterocycle fused to ethano bridge (type **III**), fused heterocycle to the benzene moiety (type **IV**), and heterocycle between the two benzene rings of paracyclophane (type **V**). 

A [2.2]paracyclophanyl acetic acid enantiomer was tested as anti-inflammatory agent [12]. Recent studies reported that 1,3-thiazoles derivatives showed in vitro α-glucosidase inhibitory activity [13,14]. 

Recently, we have an interest in the preparation of heterocycles of similar structural features [15,16,17,18,19,20,21,22,23]. In addition, we prepared metal complexes of thiosemicarbazones with Cu(I) and Cu(II) and the tridentate and bidentate structures of the paracyclophanyl–thiosemicarbazone–metal complexes were elucidated [24]. 

**Figure 2:**
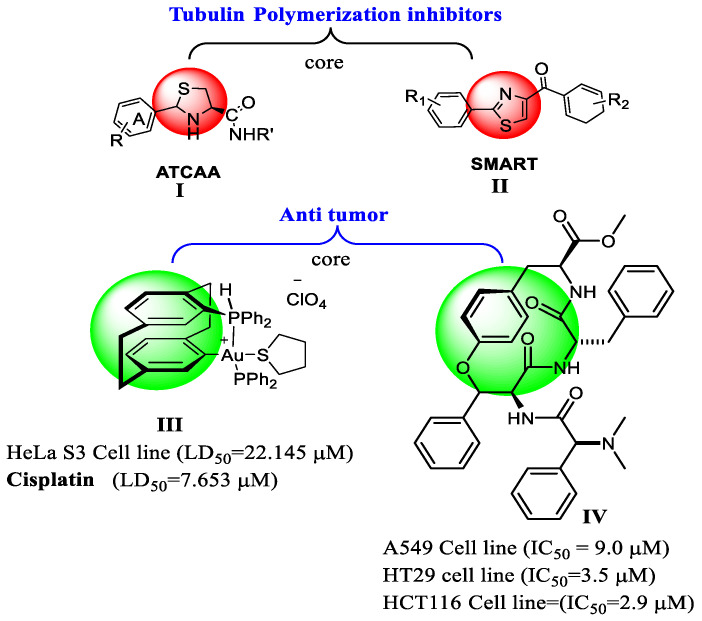
Structures of biologically active thiazole and paracyclophane derivatives.

The thiazole scaffold possesses potent cytotoxic activity in cancer cell lines. For example, thiazolidine-4-carboxylic acid amides (ATCAA) **I** showed activity against prostate cancer cells with an average IC_50_ in the range from 0.7 to 1.0 μM, while the average IC_50_ against melanoma cells ranged from 1.8 to 2.6 μM (Figure 2) [25]. 

**Figure 3:**
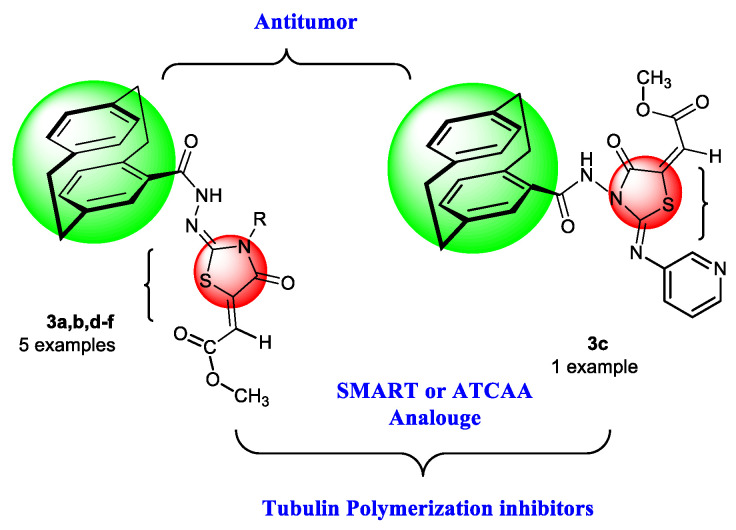
Design of the target compounds **3a**–**f.**

Moreover, gold(I) complexes of phosphino [2.2]paracyclophane ligands **III** exhibited their cytotoxic activity in the HeLa S3 cell line (LD_50_ = 22.15 μm) in comparison to cisplatin (LD_50_ = 7.65 μm). Their cytotoxicity and their mechanisms of action are different and involve apoptosis, necrosis, and DNA damage (Figure 2) [26]. Studies reported that methoxylbenzoylaryl-thiazole (SMART) **II** compounds showed nanomolar activity in inhibiting melanoma and prostate cancer cell growth [27,28,29]. Due to the interest in developing new anti-cancer agents, we designed a strategy of gathering both thiazole and paracyclophane skeletons for designing new anti-tumor agents (Figure 3).

## 2. Results and Discussion

### 2.1. Chemistry Section

**Scheme 1:**
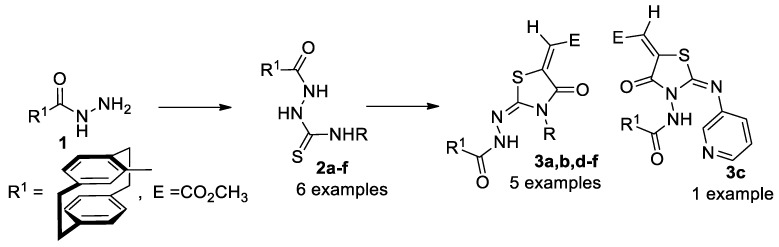
Strategy of the structure features planned to obtain the following compounds from acylhydrazide **1**: acylthiosemicarbzides **2a**–**f**, thiazolidin-3-ones **3a,b,d**–**f**, and the regioisomer **3c** derived from [2.2]paracyclophane.

Starting with acyl hydrazide **1** [30], the acylthiosemicarbazides **2a**–**f** could be prepared (Scheme 1). Compounds **2a**–**f** were used as the target compounds for the formation of thiazolidinones, **3a,b,d**–**f,** and **3c** (Scheme 1). 

**Scheme 2:**
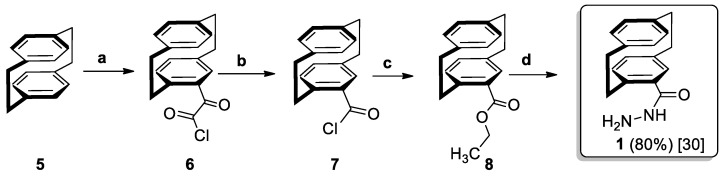
The preparation of acylthiosemicarbazide **1** from [2.2]paracyclophane (**5**): (a) (COCl)_2_/AlCl_3_, −10 °C to 5 °C, 20 min; (b) PhCl, ∆, 40 h; (c) CH_3_CH_2_OH, ∆, 10 h; (d) NH_2_NH_2_ as a solvent, ∆, 5 h.

Synthesis of compound **1** can be achieved from the reported route starting with the commercial hydrocarbon **5** (Scheme 2). The procedure consisted firstly of the conversion of **5** into the keto acid chloride **6** with oxalyl chloride/aluminum trichloride. Heating of **6** in chlorobenzene caused decarbonylation to give **7**, and, when the resulting acid chloride **7** was quenched with ethanol, the ester **8** [31] was obtained (Scheme 2). On refluxing the α-ketoester **8** with hydrazine hydrate in different solvents, the reaction failed to give the target hydrazide **1** in good yields (Scheme 2). However, heating **8** in an excess of hydrazine hydrate afforded the corresponding compound carbohydrazide **1** in 80% yield (Scheme 2).

#### Preparation of 2-(4′-[2.2]Paracyclophanyl-4*H*-hydrazinecarbothioamides **2a**–**f**

**Scheme 3:**
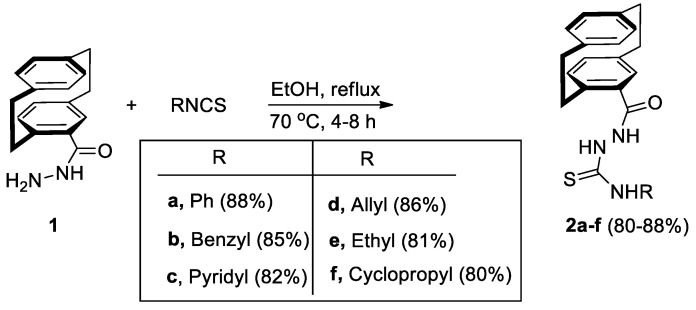
Synthesis of 2-(4′-[2.2]paracyclophanyl-4*H*-hydrazinecarbothioamides **2a**–**f.**

In the present work, 2-(4′-[2.2]paracyclophanyl-4*H*-*N*-substituted-hydrazinecarbothioamides **2a**–**f** were prepared by refluxing compound **1** with the isothiocyanates in EtOH as a solvent (Scheme 3). The infrared (IR) spectroscopy of hydrazinecarbothioamides **2a**–**f** showed that the carbonyl–amide and its N–H stretching bands were present at *ṽ* = 1656–1667 and 3360–3210 cm^–1^, respectively, in addition to a band at *ṽ* = 1390–1360 cm^−1^ due to the stretching vibration of the C=S groups. This fact was confirmed by the appearance of the carbon signal in the ^13^C-NMR at δ = 181.2–182.9 ppm. Spectroscopic details are shown from compound **2d**, as an example. The ^1^H-NMR spectrum of **2d** showed the two thiourea-NH protons at δ = 9.01 and 8.65 ppm. The paracyclophanyl protons resonated at δ = 6.96 as a doublet (*J* = 2.0 Hz) for 1H, a triplet at δ = 6.72 (*J* = 7.8, 1.9 Hz) for 2H, and at δ = 6.66–6.22 ppm as a multiplet for 4H. The allyl protons resonated as three multiplets at δ = 5.97–5.87 (CH=), 5.09–5.05 (CH_2_=), and 4.32–4.24 (CH_2_N). 

In the ^13^C-NMR spectrum, the C=S carbon appeared at δ = 182.7, whereas the C=O carbon appeared at δ = 167.7 (C=O). The allyl carbons resonated at δ = 134.8, 115.0, and 56.0 for the allyl-CH=, allyl-CH_2_=, and allyl-CH_2_, respectively. The four carbons of the paracyclophanyl CH_2_ appeared at δ = 34.8 (1C), 34.7 (1C), and 34.5 (2C) ppm. 

**Figure 4:**
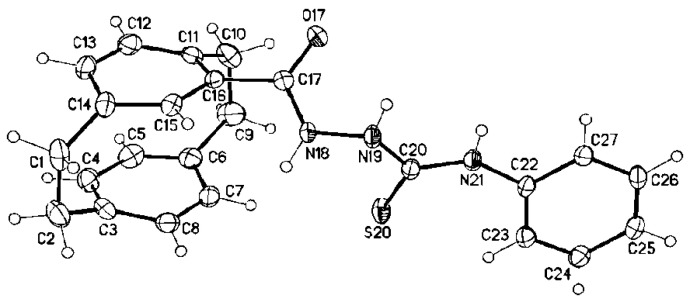
Molecular structure of compound **2a** identified according to IUPAC nomenclature as 2-(1,4(1,4)-dibenzenacyclohexaphane-1^2^-carbonyl)-*N*-phenylhydrazine-1-carbothioamide.

**Figure 5:**
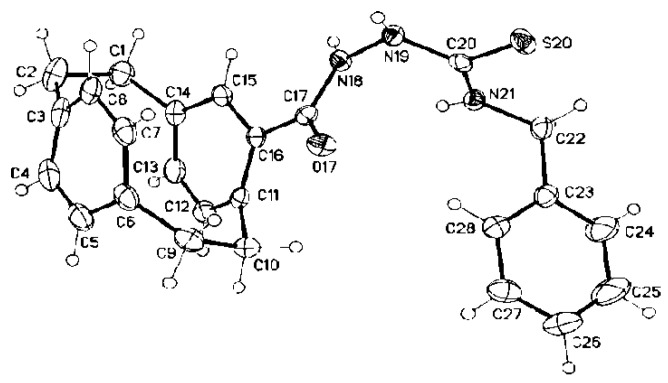
Molecular structure of compound **2b** identified according to IUPAC nomenclature as 2-(1,4(1,4)-dibenzenacyclohexaphane-1^2^-carbonyl)-*N*-benzylhydrazine-1-carbothioamide.

**Figure 6:**
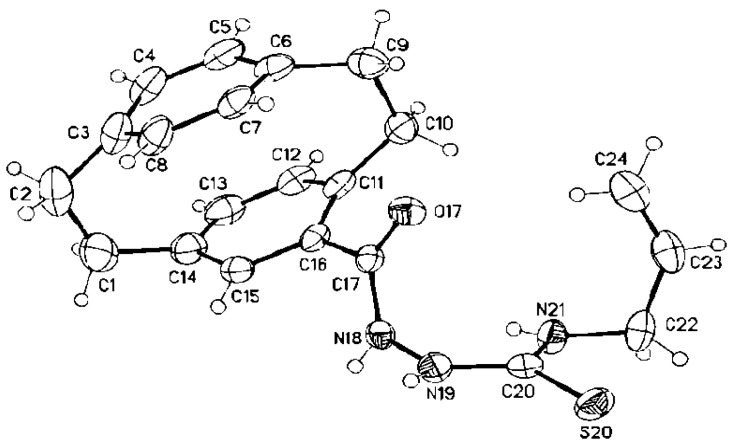
Molecular structure of compound **2d** identified according to IUPAC nomenclature as 2-(1,4(1,4)-dibenzenacyclohexaphane-1^2^-carbonyl)-*N*-vinylhydrazine-1-carbothioamide.

The X-ray structure analysis of compounds **2a,b,d** strongly confirmed the proposed structures as shown in Figure 4, Figure 5 and Figure 6, respectively. One can note that the dihedral angle of CS–NH–NH–CO was nearly 90°, and that angle was also seen in an example reported in reference [32].

**Scheme 4:**
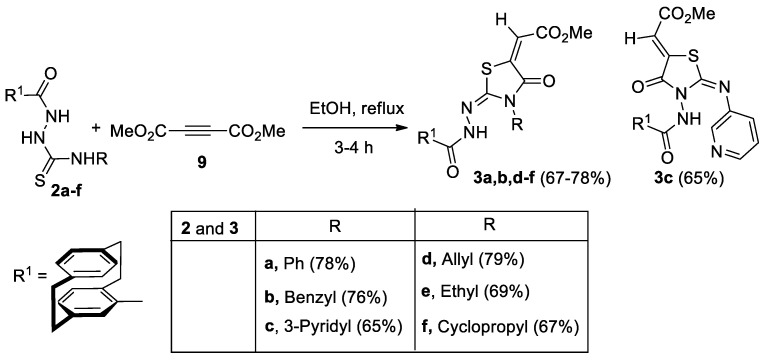
Reaction of **2a**–**f** with dimethyl acetylenedicarboxylate (**9**): synthesis of different regioisomers thiazolidines **3a,b,d**–**f** and **3c.**

Refluxing **2a**–**f** with dimethyl acetylenedicarboxylate (**9**) in EtOH afforded the expected thiazolidinones **3a,b,d**–**f** in 67%–78% yield together with **3c** in 65% yield (Scheme 4). 

**Figure 7:**
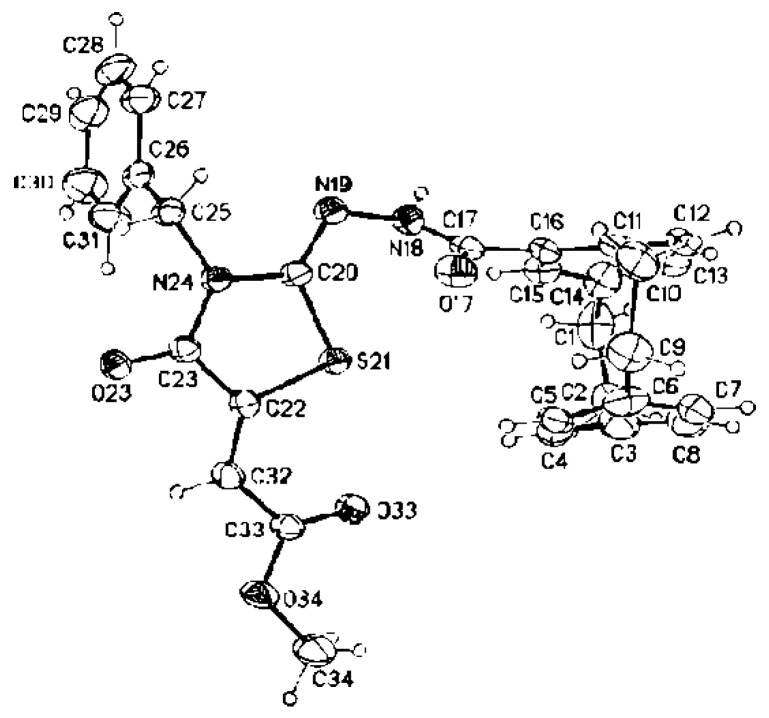
Molecular structure of compound **3b** identified according to IUPAC nomenclature as methyl (*E*)-2-((*E*)-2-(2-(1,4(1,4)-dibenzen-cyclohexaphane-12-carbonyl)hydrazinylidene)-3-benzyl-4-oxothiazolidin-5-ylidene)acetate.

Mass spectroscopy and elemental analysis identified the molecular formula of **3b** as C_30_H_27_N_3_O_4_S. Moreover, the ^1^H-NMR spectrum revealed three singlets at δ = 10.88 (NH), 6.93 (vinyl-CH), and 5.44 (benzylic-CH_2_). The ^13^C-NMR spectrum of **3b** showed three carbonyl carbon signals at δ = 166.0 (ester-CO), 165.6 (cyclic-C═O), and 161.5 (hydrazide-CO). The thiazolidine-4-one moiety showed carbon signals at δ =146.2 for C-2 and 140.2 for C-5. In addition, the vinyl-CH, OCH_3_-ester, and benzyl-CH_2_ resonated at δ =116.6, 52.6, and 51.3 ppm, respectively. The X-ray structure analysis of **3b** identified the structure as methyl (*E*)-2-((*E*)(2′-4-[2.2]paracyclohanyl)-hydrazinylidene)-3-benzyl-4-oxathiazolidin-5-ylidene)acetate (Figure 7). 

**Figure 8:**
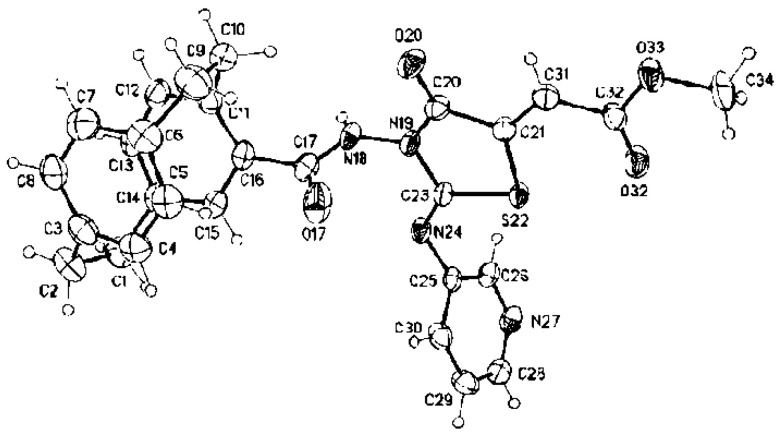
Molecular structure of compound **3c** identified according to IUPAC nomenclature as methyl (*E*)-2-((*E*)-3-(1,4(1,4)-dibenzenacyclohexaphane-1^2^-carboxamido)-4-oxo-2-(pyridin-3-ylimino)thiazolidin-5-ylidene)acetate.

The ^1^H-NMR spectrum showed two singlets at δ = 10.12 (NH) and 6.57 (vinyl-CH). The CO-ester, cyclic-CO, hydrazide-CO, thiazolidine-C-,2 and thiazolide-C-4 appeared in the ^13^C-NMR spectrum of **3c** at δ = 167.0, 166.6, 162.5, 151.3, and 141.1, respectively (see Section 3). Finally, the structure of **3c** was ambiguously identified by X-ray structure analysis (Figure 8). 

**Scheme 5:**
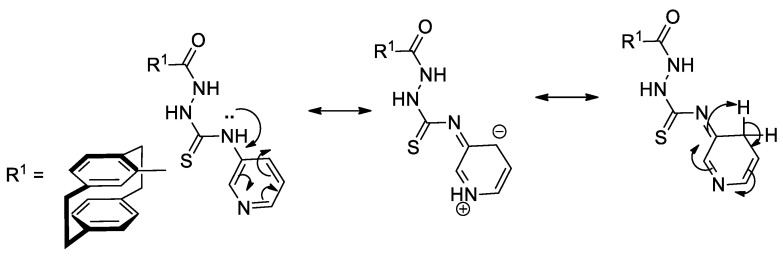
Resonance structures of compound **3c.**

The abnormal behavior of **2c** toward **9** might be attributed to the expected resonance structure of **2c** that would decrease the basicity of the *N^3^*-thioamide compared with the *N^2^*-hydrazide and, therefore, the *N^2^*-hydrazide would be more reactive toward nucleophilic addition (Scheme 5).

### 2.2. Biological Investigation

#### 2.2.1. Anti-Proliferative Investigation against 60 Cancer Cell Lines at the National Cancer Institute (NCI), USA.

The methodology of the NCI anticancer screening was described in detail elsewhere (http://www.dtp.nci.nih.gov). Briefly, the primary anticancer assay was performed using approximately 60 human tumor cell lines derived from nine neoplastic diseases, in accordance with the protocol of the Drug Evaluation Branch, National Cancer Institute, Bethesda. Tested compounds were added to the culture at a single concentration (10^−5^ M) and the cultures were incubated for 48 h. End-point determinations were made with a protein binding dye, SRB. Results for each tested compound were reported as the percentage growth of the treated cells when compared to the untreated control cells. The percentage growth was evaluated spectrophotometrically versus controls not treated with test agents. All experiments were repeated three times (Table 1). 

Compounds **3a**–**f** revealed that compound **3a** achieved complete cell death on the nine tested cancer cell lines. It is noteworthy that compounds **3a, 3b, 3d**, and **3e** were the most potent tested derivatives on the leukemia cell line. They showed growth inhibition percentages greater than 100%, which means that they were cytotoxic and displayed complete cancer cell death that killed all cells, including cancer cells. They may stop cancer cells from dividing and growing and may cause tumors to shrink in size. The complete cell death was against leukemia RRMI-8226 with inhibitions of 120.89%, 147.00%, 109.36%, and 114.28%, respectively, and against SR with inhibitions of 115.60%, 114.70%, 98.21%, and 113.40%, respectively. Compound **3e** showed remarkable activity on the other tested cell lines. Although **3f** showed moderate to weak activity on most of tested cancer cell lines, compound **3b** exhibited significant inhibition against non-small-cell lung cancer NCI-H522, colon cancer HT29 and SW-620, melanoma LOX IMVI, ovarian cancer OVCAR-3, renal cancer CAKI-1, prostate cancer PC-3, and breast cancer BT-549, T-47D, and MDA-MB-468 with inhibitions of 84.06%, 87.62%, 81.83%, 93.79%, 79.71%, 84.43%, 82.23%, 89.68%, 106.25%, and 78.91%, respectively. Furthermore, compounds **3c** and **3d** displayed mild to moderate activity on most of the cancer panel cell lines

##### Structure Activity Relationship (SAR)

It is notable that the new prepared paracyclophane/thiazole conjugates showed significant anti-cancer activity with different growth inhibition percentages. This disparity among the different derivatives may be attributed to the type of substitution on the thiazole ring, whether on nitrogen (compounds **3a,b,d**–**f**) or at position 2 (compound **3c**). It is expected that increasing thiazole flexibility through inserting a phenyl or benzyl group (compounds **3a** and **3b**, respectively) into the structure would improve binding to the target protein and, hence, show higher antiproliferative activity on all tested cell lines than those containing an aliphatic group, i.e., either compound **3d** (allyl) or **3e** (ethyl) of the same series, which provides less flexibility. Interestingly, the other paracyclophane/thiazole derivative **3c** bearing a pyridinyl amine moiety at position 2 of the thiazole ring altered the activity, showing lower cytotoxicity.

#### 2.2.2. In Vitro Five-Dose Full NCI 60 Cell Panel Assay

**Figure 9:**
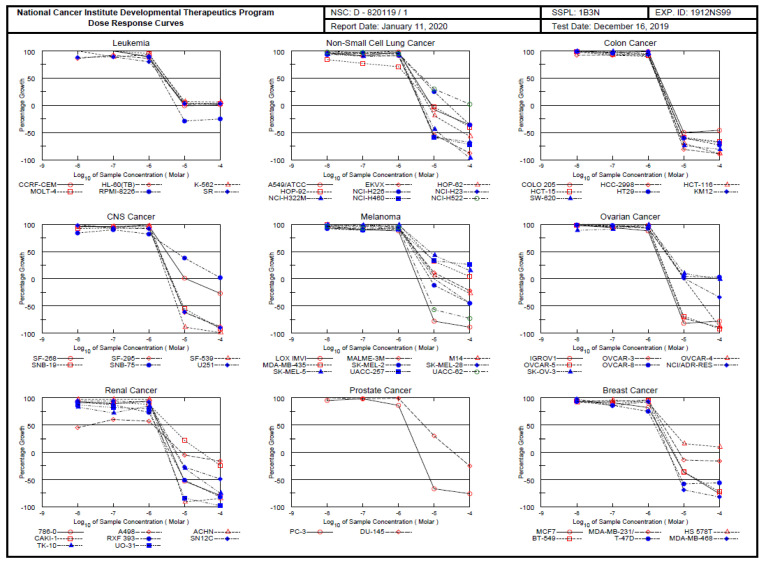
Dose–response curves of compound **3a** (log_10_ of sample concentrations 0.01, 0.1, 1, 10, and 100 μM) against all nine different cancer cell line panels (leukemia, non-small-cell lung, colon, CNS, melanoma, ovarian, renal, prostate, and breast cancers).

Compound **3a** was selected by NCI for five-dose investigation against 60 human tumor cell lines that were incubated at five different concentrations (0.01, 0.1, 1, 10, and 100 μM) (Figure 9). The outcomes were used to form log concentration vs. percentage growth inhibition curves and three response parameters (GI_50_, total growth inhibition (TGI), and LC_50_) were calculated for each cell line (Table 2). The GI_50_ value (growth inhibitory activity) corresponds to the concentration of the compound causing 50% decrease in net cell growth, the TGI value (cytostatic activity) is the concentration of the compound resulting in total growth inhibition (TGI), and the LC_50_ value (cytotoxic activity) is the concentration of the compound causing a net 50% loss of initial cells at the end of the incubation period of 48 h. 

The criterion for selectivity of a compound depends upon the ratio obtained by dividing the full-panel MID (the average sensitivity of all cell lines toward the test agent) (μM) by the individual subpanel MIDs (μM). Ratios between 3 and 6 refer to moderate selectivity, and ratios greater than 6 indicate high selectivity toward the corresponding cell line, while compounds not meeting either of these criteria are rated non-selective. 

Compound **3a** under investigation exhibited remarkable anticancer activity against most of the tested cell lines representing nine different subpanels with GI_50_ ranging from 1.52–7.56 μM (Table 2). Results indicate that **3a** showed high activity against renal cancer RXF-393, melanoma LOX IMIV, colon cancer HCC-2998, non-small-cell lung cancer EKVX, and leukemia RPMI-8226 with GI_50_ values of 1.52, 1.69, 1.78, 1.91, and 2.15 μM, respectively. An obvious sensitivity profile was seen toward the leukemia subpanel (GI_50_ values ranging from 2.15 to 3.15 μM), colon cancer subpanel (GI_50_ values ranging from 1.78 to 2.13 μM), breast cancer subpanel (GI_50_ values ranging from 1.54 to 1.87 μM), and ovarian cancer subpanel (GI_50_ values ranging from 1.66 to 3.55 μM). In this context, compound **3a** was found to have broad-spectrum antitumor activity against the nine tumor subpanels tested with selectivity ratios ranging from 0.63–1.28 and 0.58–5.89 at the GI_50_ and TGI levels, respectively; however, it exhibited noteworthy antiproliferative activity against all cancer cell lines with low selectivity ratios.

#### 2.2.3. Evaluation of In Vitro Antiproliferative Activities against Leukemia RPMI-8226 and SR

Since the antiproliferative investigation results against 60 cell lines at the NCI revealed greater activity toward leukemia cancer, especially leukemia RPMI-8226 and SR, we were encouraged to perform further in vitro antiproliferative studies against those two cell lines. Compounds **3a**–**e** were evaluated for their antiproliferative activity by performing an MTT assay against a panel of two human tumor cell lines, leukemia RPMI-8226 and SR, compared with colchicine as a reference. As shown in Table 3, the antiproliferative activities of the tested compounds were generally more pronounced against the two panels of leukemia cancer cells as compared with the reference. The calculated results were subjected to statistical analysis using GraphPad Prism 7 with the one-way ANOVA and non-parametric program. The difference in the results was considered significant when the *p*-values were less than 0.05. All the tested compounds were significant, as well as the reference (*** *p* < 0.05), in comparison to control. Compound **3a** exhibited the highest antiproliferation compared to reference and the other tested compounds, whereas it showed IC_50_ values 1.61 and 1.11 µM better than colchicine (i.e., the reference compound) of 4.05 and 1.81 µM against leukemia RPMI-8226 and SR, respectively. On the other hand, compound **3e** showed a significant antiproliferative activity with an IC_50_ value 3.17 µM better than the reference of 4.05 µM against leukemia RPMI-8226 only. This may be attributed to both compounds **3a** and **3b** having electron-withdrawing substitution of phenyl and benzyl, respectively, which positively affected their permeability to cancer cells. Compound **3b** showed comparable IC_50_ values of 4.62 and 2.02 µM to colchicine. 

Additionally, compound **3c** bearing a pyridinyl amine moiety at position 2 of the thiazole ring showed weak anti-proliferation activity with IC_50_ values of 9.69 and 4.84 µM, which explains its low cytotoxicity. It is interesting to mention that the proliferation inhibitory results were positively correlated with the anticancer results obtained from NCI. 

#### 2.2.4. Evaluation of In Vitro Tubulin Polymerization Inhibitory Activity

To investigate whether the antiproliferative activities of these target compounds **3a**–**e** were related to their interaction with tubulin, these compounds were tested for their ability to inhibit tubulin polymerization at their IC_50_ concentrations using an ELISA assay for β-tubulin.

The in vitro kinetics of microtubule assembly was measured using an ELISA kit for TUBb (Cloud-Clone. Corp.) on the leukemia SR cell line. The compounds tested were **3a–f** and colchicine. Briefly, growing cells from the SR cell line were trypsinized, counted, and seeded at the appropriate densities into 96-well microtiter plates. Cells were then incubated in a humidified atmosphere at 37 °C for 24 h.

The assay revealed that all the tested compounds **3a**–**e** showed tubulin polymerization inhibitory activity compared to colchicine as a reference (Table 4). Again, compound **3a** showed the highest ability to inhibit tubulin polymerization with an IC_50_ value of 4.97 µM compared to the reference with an IC_50_ value 3.76 µM and the other tested compounds. On the other hand, compounds **3e** and **3c** showed remarkable tubulin polymerization inhibition with IC_50_ values of 6.61 and 8.38 µM, while **3d** displayed weak inhibition with an IC_50_ value of 14.79 µM. Results are in agreement with the previous mentioned anti-proliferative activity

#### 2.2.5. Cell Cycle Analysis

Cell cycle analysis was performed using cytometers from Becton Dickinson Immunocytometry Systems, Beckman/Coulter Inc., DACO/Cytomation, and PARTEC GmbH. Regulation of cell growth is mainly controlled through cell-cycle control mechanisms. Proliferation inhibition can be triggered by cell-cycle arrest in cancer cells. During the cell cycle, the G2/M checkpoint is a potential target for cancer therapy.

**Figure 10:**
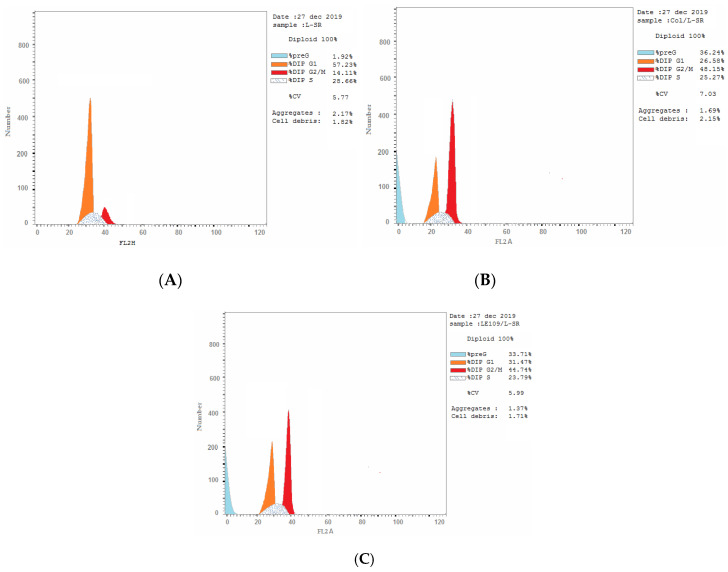
Cell-cycle analysis of SR cells treated with annexin PI at IC_50_. Concentration representing growth arrest at the pre-G1 (G0) and G2/M phases. (**A**) Untreated cells; (**B**) treated cells with colchicine; (**C**) treated cells with **3a.** The test was repeated three times, while **3a** and the reference were incubated for 24 h (2 × 10^5^ cells/well) at 37 °C.

**Figure 11:**
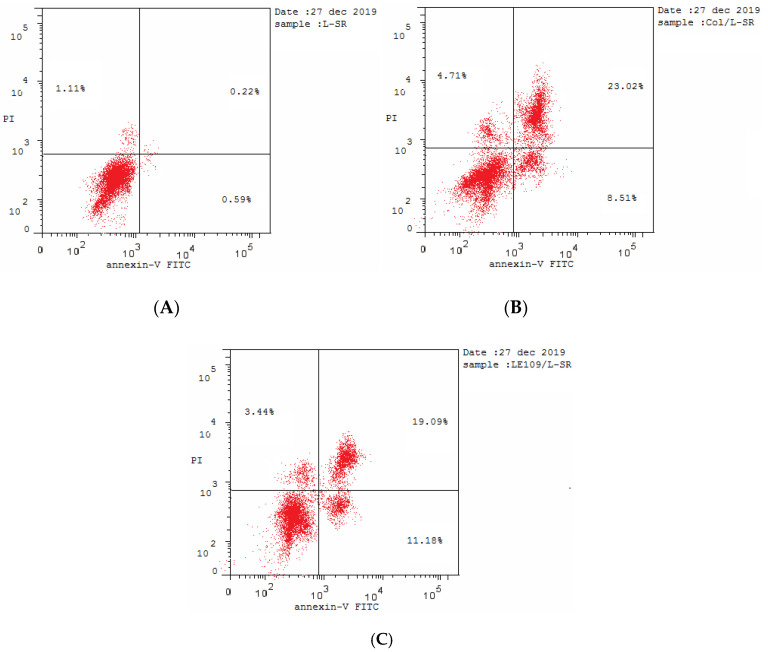
Contour diagram of annexin V/PI flow cytometry against SR cancer cell line. (**A**) Untreated cells; (**B**) treated cells with colchicine; (**C**) treated cells with **3a**. The test was repeated three times. Following incubation for 24 h, cells were analyzed by flow cytometry after double staining with annexin V/PI.

This prevents DNA-damaged cells from entering mitosis and allows for the repair of DNA that was damaged in late S or G2 phases prior to mitosis (Table 5). Induction of cell-cycle arrest is a common mechanism proposed for the cytotoxic effects of anticancer drugs containing paracyclophane/thiazole derivatives. The analysis indicated that leukemia SR cells treated with compound **3a** showed significant growth arrest at the G2/M phase compared to control cells, where the S-phase progression of SR cells was substantially delayed (Figure 10). The annexin V/PI flow cytometry of SR cells was repeated three times after treatment with the IC_50_ value (1.11 µM) of **3a**, which showed an increase in percentage of the necrotic cells in late apoptosis to 19% (upper right quadrant of the cytogram) (Figure 11). Hence, compound **3a** showed a considerable ability to dissipate cell membrane integrity, whereas the lower right quadrant illustrating the early apoptotic cells which kept their membrane integrity indicated the ability of **3a** to initiate apoptosis.

#### 2.2.6. Compound **3b** Induced Mitochondrial Depolarization and ROS Production

**Figure 12:**
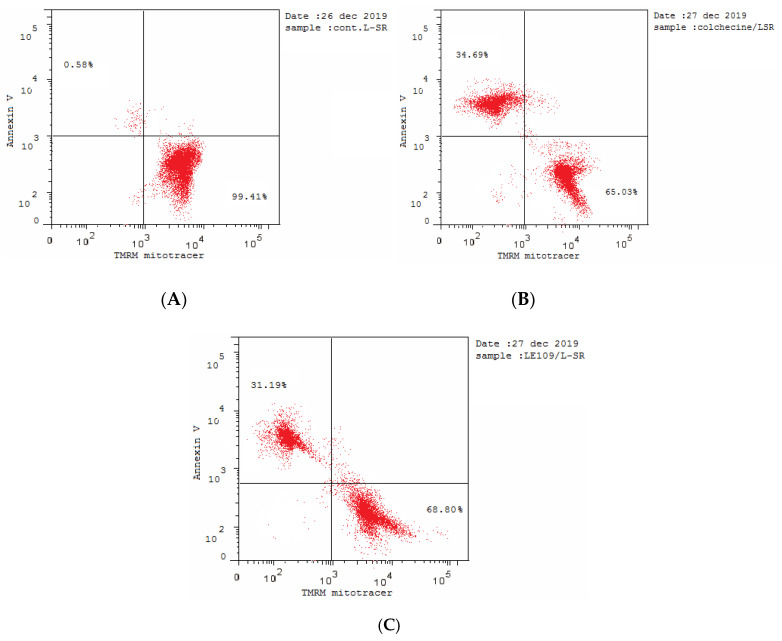
Contour diagram of annexin V/PI flow cytometry illustrating induction of the loss of mitochondrial membrane potential (Δψmt) against SR cancer cell line. (**A**) Untreated cells; (**B**) treated cells with colchicine; (**C**) treated cells with **3a**. (B and C were performed at concentrations of 20, 10, 5, 2.5, 1.2, 0.63, and 0.31 ng/mL). The tests were repeated three times and the substrate was incubated at 37 °C in the dark for 15–30 min.

Mitochondria play an essential role in the propagation of apoptosis [33]. It is reported that, at an early stage, apoptotic stimuli are capable of modifying the mitochondrial transmembrane potential (Δψmt). Δψmt was recorded by the fluorescence of the dye JC-1.20. SR cells treated with **3a** displayed a non-significant shift in fluorescence compared with control cells, explaining the weak depolarization of the mitochondrial membrane potential. In contrast, colchicine showed significant results (Figure 12). The disruption of Δψmt is associated with the appearance of annexin V positivity in the treated cells when they are in an early apoptotic stage. It is documented that the dissipation of Δψmt is characteristic of apoptosis, and this was indicated with both microtubule-stabilizing and -destabilizing agents in different cell types [34]. Induction of cell-cycle arrest is a common mechanism proposed for the cytotoxic effects of anticancer drugs containing paracyclophane/thiazole derivatives. Mitochondrial membrane depolarization is associated with mitochondrial production of ROS [35]. Therefore, we investigated whether ROS production increased after treatment with the test compounds. 

**Figure 13:**
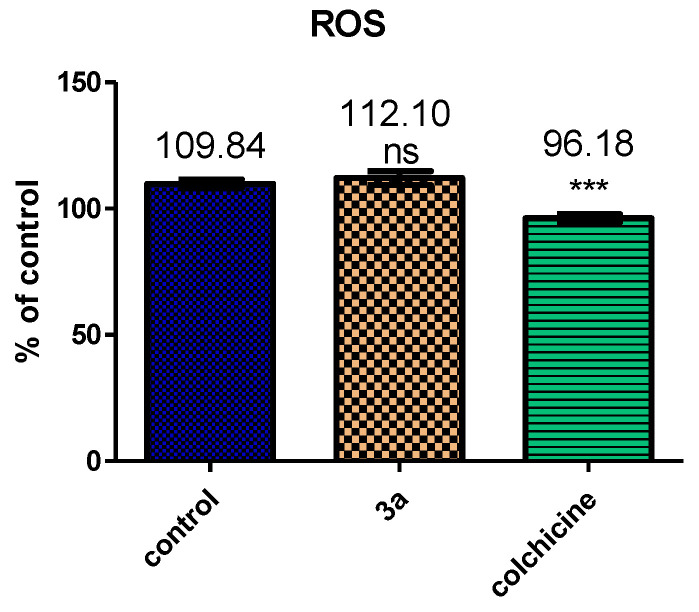
Mitochondrial production of ROS in SR cells with **3a** and colchicine reference compared to control. Mean ± standard deviation plotted for three replicates per condition. Results are significantly different from control at *** *p* < 0.05. The substrate was incubated for 30 min at 37 °C protected from light.

The presented results in Figure 13 show that **3a** induced the production of significant amounts of ROS (109.84%) in comparison with control cells and the colchicine reference. This result is compatible with the dissipation of Δψmt described above.

#### 2.2.7. Effect of Compound **3a** on Multidrug-Resistant (MDR) Leukemia SR Cells

A previous study reported that multidrug resistance was observed for numerous chemotherapeutic agents and could be identified through overexpression of P-glycoprotein (Pgp) [36,37].

Compound **3a** and tubulin-targeting agent colchicine were tested on the MDR-SR Leukemia cell line. Compound **3a** showed Pgp-mediated MDR expression at 1.285 ng/mL (1.15-fold change), which means that it had much better resistance indices comparable to control at 1.121 ng/mL (1-fold) than colchicine at 1.726 ng/mL (1.54-fold change). Results of compound **3a** (Table 6) showed the ability of overcoming the assumed glycoprotein overexpression of the cell line; thus, **3a** could be considered as an important substrate candidate against MDR cells.

#### 2.2.8. Effect of **3a** on Caspase-3 Activation

Since caspase-3 is important for spreading the apoptotic signal after exposure to antimitotic compounds [38], the effect of compound **3a** on the caspase-3 activated enzyme was evaluated using ELISA and replicated three times. The reference compound and **3a** were incubated for 30 min at room temperature in the dark on the leukemia SR cell line. Results revealed that **3a** is a potential caspase-3 activator with a slight increase in the level of active caspase-3 at a concentration of 471.2 ng/mL (8.84-fold) compared to colchicine at a concentration of 428.9 ng/mL (8.05-fold) as shown in Table 7.

#### 2.2.9. Effect of **3a** on BAX and Bcl-2 Proteins 

**Figure 14:**
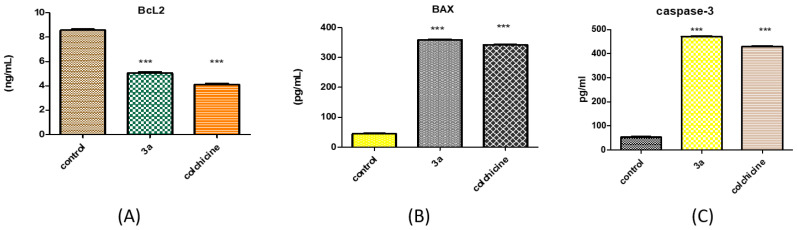
Concentrations of (**A**) Bcl-2 (concentrations 32, 16, 8, 4, 2, 1, 0.5 ng/mL); (**B**) BAX (concentrations 2000, 1000, 500, 250, 125, 62.5, 31.25 pg/mL), and (**C**) caspase-3 expression (2500, 1250, 625, 313, 156, 78, 39 pg/mL) for **3a** and colchicine on leukemia SR cell line relative to control. Results significantly different from control at *** *p* < 0.05.

The proteins of the Bcl2 family [39] play a major role in controlling apoptosis through the regulation of mitochondrial processes and the release of mitochondrial proapoptotic molecules that are important for the cell death pathway [40]. Compound **3a** caused nearly 7.98-fold upregulation (Table 8), while it showed markedly higher levels of the antiapoptotic Bcl-2 proteins up to 0.59-fold compared to the control untreated cells (Figure 14).

### 2.3. Docking Studies

**Figure 15:**
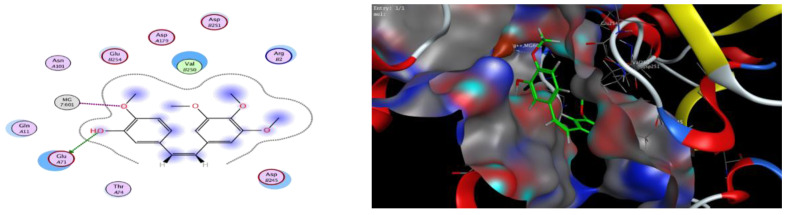
The two- and three-dimensional (2D and 3D) diagrams illustrate the binding mode of the reference colchicine interacting with the colchicine binding site of β-tubulin.

**Figure 16:**
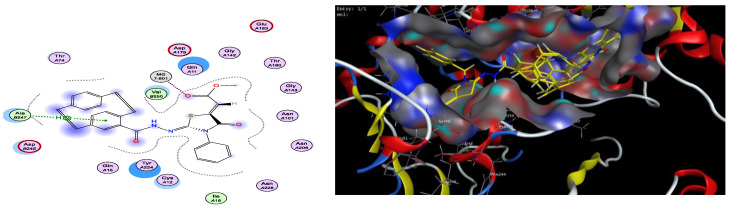
The 2D and 3D diagrams illustrate the binding mode of **3a** interacting with the colchicine binding site of β-tubulin.

**Figure 17:**
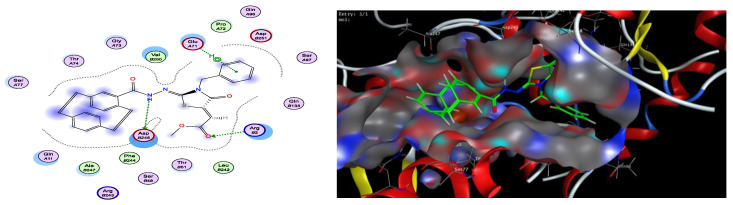
The 2D and 3D diagrams illustrate the binding mode of **3b** interacting with the colchicine binding site of β-tubulin.

**Figure 18:**
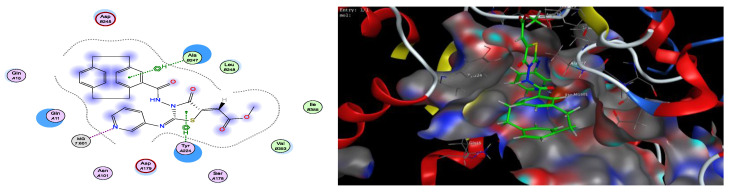
The 2D and 3D diagrams illustrate the binding mode of **3c** interacting with the colchicine binding site of β-tubulin.

**Figure 19:**
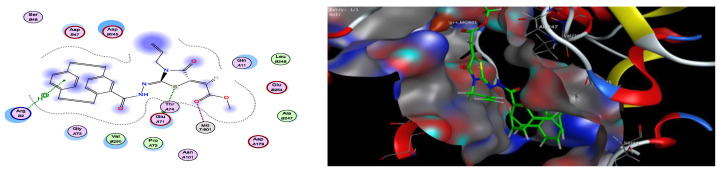
The 2D and 3D diagrams illustrate the binding mode of **3d** interacting with the colchicine binding site of β-tubulin.

**Figure 20:**
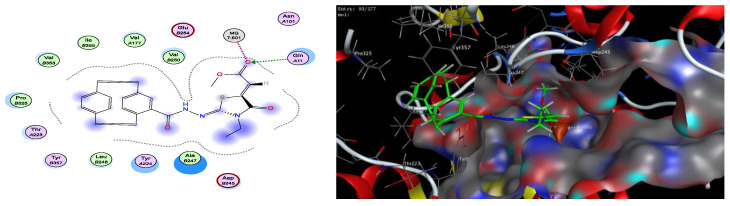
The 2D and 3D diagrams illustrate the binding mode of **3e** interacting with colchicine binding site of β-tubulin.

The molecular modeling of the possible binding modes for the newly synthesized thiazole/paracyclophane hybrids **3a**–**e** and colchicine as a reference was done to predict the binding interactions between them and β-tubulin at the colchicine binding site, which was obtained from the protein data bank (PDB: 3HKC). Docking studies were carried out using Molecular Operating Environment (MOE^®^) version 2014.09 (Chemical Computing Group Inc., Montreal, QC, Canada) using colchicine as a reference for validation of the method. The theoretical predictions from the molecular docking study agreed for some highly active derivatives such as **3a, 3b, 3d**, and **3e** with the experimentally observed tubulin polymerization inhibition. All derivatives were successfully docked into the colchicine binding site of β-tubulin. The binding free energies from the major docked poses are listed in Table 9, and the most favorable poses of the tested compounds are shown in Figure 15, Figure 16, Figure 17, Figure 18, Figure 19 and Figure 20. Most of the tested compounds had high binding affinity to the enzyme as the binding free energy (ΔG) values of them ranged from −0.5 to −3.4 kcal/mol, which was comparable to the reference colchicine (ΔG = −0.6 to −2.3 kcal/mol). The docking result of the reference compound colchicine was completely consistent with the mode of action of thiazole/paracyclophane derivatives (Figure 15).

The two-dimensional (2D) diagram showed crucial binding involving Ala 247, Tyr 224, and Mg 601 through the [2.2]paracyclophane ring, thiazole ring, and ester C=O functionality. Moreover, stabilization of the reference colchicine within the active site occurred through one strong hydrogen bond interaction with amino-acid residue Glu 71 and metal intercalation with Mg 601. Docking results with the colchicine binding site revealed that most of the tested compounds showed good binding with the enzyme and made several interactions comparable to that of the reference colchicine (Figure 15).

Compounds **3a**, **3c**, **3d**, and **3e** exhibited the same interaction as the reference with Mg 601; however, both **3d** and **3b** showed hydrogen bonding interactions with Glu 71 (Figure 16, Figure 17, Figure 18, Figure 19 and Figure 20). On the other hand, compound **3d** possessed greater interactions than the reference with the same amino acids with additional one H–pi bond with Arg 2 (Figure 20); however, compounds **3a** and **3c** displayed an extra H–pi interaction with Ala 247 (Figure 16 and Figure 18). Compound **3c** (Figure 18) did not feature hydrogen binding interactions with the amino acid residue Glu 71, but kept an H–Pi interaction with Tyr 224**.**

## 3. Experimental

### 3.1. Chemistry Part

#### 3.1.1. Materials and Methods

Melting points were taken in open capillaries on a Gallenkamp melting point apparatus (Weiss-Gallenkamp, Loughborough, UK) and are uncorrected. The IR spectra were recorded using the attenuated total reflection (ATR) technique with a FT device (FT-IR Bruker IFS 88, Bruker, Leiderdorp, The Netherlands), Institute of Organic Chemistry, Karlsruhe Institute of Technology, Karlsruhe, Germany. The NMR spectra were measured in DMSO-*d*_6_ and acetone-*d_6_* on a Bruker AV-400 spectrometer at 400 MHz for ^1^H and 100 MHz for ^13^C; the chemical shifts are expressed in δ (ppm) versus internal tetramethylsilane (TMS) = 0 for ^1^H and ^13^C, and external liquid ammonia = 0. The description of signals includes s = singlet, d = doublet, t = triplet, q = quartet, m = multiplet, dd = doublet of doublet, ddd = doublet of dd, dt = doublet of triplet, td = triplet of doublet, bs = broad singlet, and m = multiplet. Mass spectra were recorded on a FAB (fast atom bombardment) Thermo Finnigan Mat 95 (70 eV). For the high-resolution mass, the following abbreviations were used: calc.= theoretical calculated mass; found = mass found in analysis at the Institute of Organic Chemistry, Karlsruhe University, Karlsruhe, Germany. Thin Layer Chromatography (TLC) was performed on analytical Merck 9385 silica aluminum sheets (Kieselgel 60) with Pf_254_ indicator; TLCs were viewed at λ_max_ = 254 nm. Crude products were purified by flash chromatography with silica gel 60 (0.040 × 0.063 mm, Geduran^®^) (Merck, Darmstadt, Germany). Compounds **2** and **5**–**8** were prepared according to the methodology mentioned in References [30] and [31], respectively.

#### 3.1.2. Preparation of Paracyclophanyl-*N*-substituted Hydrazinecarbothioamides **2a**–**f**

A mixture of carbohydrazide paracyclophane (**1**) (1.00 g, 3.7 mmol) in 60 mL of ethanol and different derivatives of isothiocyanate (3.7 mmol) was refluxed for 4–8 h. The reaction mixture was poured into a beaker and was allowed to stand until a precipitate was formed. Then, the precipitate was filtered and washed with heptane several times (3 × 100 mL).

*2-(4′-[2.2]Paracyclophanyl)-N-phenylhydrazinecarbothioamide* (**2a**). Colorless crystals (ethanol), 1.33 g (88%), melting point (m.p.) 190–92 °C, ^1^H-NMR (400 MHz, DMSO-*d_6_*, ppm): δ = 9.86 (s, 1H, NHNHCS), 9.63 (s, 1H, NH-CO), 7.55 (d, *J* = 7.8 Hz, 1H, Ph-H), 7.50 (s, 1H, NHCS), 7.33 (t, *J* = 7.9 Hz, 2H, Ph-H), 7.14 (t, *J* = 7.6 Hz, 2H, Ph-H), 6.95 (s, 1H, PC-H), 6.63 (dd, *J* = 7.8, 1.8 Hz, 2H, PC-H), 6.52 (d, *J* = 8.0 Hz, 3H, PC-H), 6.47 (d, *J* = 7.8 Hz, 1H, PC-H), 3.76 (s, 1H, PC-CH_2_-2′), 3.33–2.79 (m, 7H, PC-CH_2_-CH_2_). ^13^C-NMR (100 MHz, DMSO-*d_6_*, ppm): δ = 181.2 (C=S), 171.8 (C=O), 140.1 (Ph-CH), 140.0 (PC-C-6′), 139.5 (PC-C-11′), 139.4 (PC-C-14′), 139.3 (PC-C-3′), 139.0 (PC-2CH), 135.7 (PC-2CH), 135.1, 132.6 (PC-CH), 132.5 (Ph-2CH), 132.4 (Ph-2CH), 131.6 (PC-CH),129.0 (Ph-C), 128.2 (PC-C-4′), 34.8 (PC-CH_2_-1′), 34.7 (PC-CH_2_-10′), 34.5 (PC-CH_2_-9′), 34.4 (PC-CH_2_-2′). IR (ATR, cm^−1^) *ṽ* = 3250–3210 (br, m, NH), 3009 (m, Ar-CH), 2890 (w, Aliph-CH), 1656 (s, CO), 1390 (s, C=S), 980 (s, C-N str). MS (FAB) *m*/*z* (%) = 402.2 ([M + H]^+^ (85). HRMS ([M + H]^+^, C_24_H_24_O_1_N_3_^32^S_1_) Calcd.: 402.1635, Found: 402.1633.

*2-(4′-[2.2]Paracyclophanyl)-N-benzylhydrazine-1-carbothioamide* (**2b**). Colorless crystal (ethanol), 1.33 g (85%), m.p. 172–174 °C, ^1^H-NMR (400 MHz, DMSO-*d_6_*, ppm) δ = 9.76 (s, 1H, NHNHCS), 9.41 (s, 1H, NH-CO), 8.34 (s, 1H, NHCS), 7.34–7.32 (m, 2H, Ph-H), 7.31–7.28 (m, 2H, Ph-H), 7.22-7.20 (m, 1H, Ph-H), 6.93 (s, 1H, PC-H), 6.64–6.61 (m, 2H, PC-H), 6.53–6.40 (m, 4H, PC-H), 4.81–4.74 (m, 2H, Benzyl-CH_2_), 3.70–3.68 (m, 1H, PC-CH_2_-2′), 3.14–2.78(m, 7H, PC-CH_2_-CH_2_). ^13^C-NMR (100 MHz, DMSO-*d_6_*, ppm) δ = 182.9 (C=S), 168.3 (C=O), 140.6 (Ph-H), 140.0 (PC-C-6′), 139.9 (PC-C-11′), 139.7 (PC-C-14′), 139.4 (PC-C-3′), 136.2 (PC-2CH), 135.7 (PC-CH), 133.1 (Ph-C), 133.0, 132.9 (PC-2CH), 131.9 (PC-C-4′), 128.5 (Ph-2CH), 127.5, 127.1 (Ph-CH), 47.2 (Benzyl-CH_2_), 35.3 (PC-CH_2_-1′), 35.2 (PC-CH_2_-10′), 35.0 (PC-2CH_2_-9′,2′). IR (ATR, cm^−1^) *ṽ* = 3346 (s, NH), 3199 (s, Ar-CH), 2980 (w, Aliph-CH), 1653 (s, CO), 1365 (s, C=S), 978 (C-N str). MS (FAB) *m*/*z* (%) = 416.2 ([M + H]^+^ (70)]. HRMS ([M + H]^+^, C_25_H_26_O_1_N_3_^32^S_1_) Calcd.: 416.1797, Found: 416.1799.

*2-(4′-[2.2]Paracyclophanyl)-N-(pyridin-3-yl)hydrazine-1-carbothioamide* (**2c**). Colorless crystal (methanol), 1.24 g (82%), 152–154 °C, ^1^H-NMR (400 MHz, DMSO-*d*_6_, ppm) δ = 9.93 (s, 1H, NHNHCS), 9.72 (s, 1H, NHCO), 8.57 (s, 1H, NHCS), 8.34 (dd, *J* = 4.8, 1.5 Hz, 1H, Pyr-H), 7.90–7.40 (m, 2H, Pyr-H), 7.38 (dd, *J* = 8.2, 4.7 Hz, 1H, Pyr-H), 7.12–6.87 (m, 2H, PC-H), 6.84–6.57 (m, 2H, PC-H), 6.58–6.48 (m, 2H, PC-H), 6.46 (d, *J* = 7.8 Hz, 1H, PC-H), 3.75–3.34 (m, 1H, PC-CH_2_-2′), 3.27–3.05 (m, 3H, PC-CH_2_-CH_2_), 3.05–2.90 (m, 3H, PC-CH_2_-CH_2_), 2.90–2.77 (m, 1H, PC-CH_2_-CH_2_). ^13^C-NMR (100 MHz, DMSO-*d*_6_, ppm) δ = 181.5 (C=S), 167.7 (C=O), 147.0, 145.7 (Pyr-CH), 140.2 (PC-C-6′), 139.4 (PC-C-11′), 139.2 (PC-C-14′), 138.9 (PC-C-3′), 136.1 (Pyr-C), 135.7 (PC-2CH), 135.2 (PC-CH), 133.4, 132.5 (PC-CH), 132.4 (Pyr-CH), 132.3 (PC-2CH), 131.4 (PC-C-4′), 122.9 (Pyr-CH), 38.8 (PC-CH_2_-1′), 34.7 (PC-CH_2_-10′), 34.6 (PC-CH_2_-9′), 34.4 (PC-CH_2_-4′). IR (ATR, cm^−1^) *ṽ* = 3206 (m, NH), 3091 (w, Ar-CH), 2925 (w, aliph-CH), 1645 (m, CO), 1376 (s, C=S), 980 (s, C-N str). MS (FAB) *m*/*z* (%) = 403.2 [M + H]^+^ (55). HRMS ([M + H]^+^, C_23_H_23_O_1_N_4_^32^S_1_) Calcd.: 403.1593, Found: 403.1591.

*N-Allyl-2-(4′-[2.2]paracyclophanyl)hydrazine-1-carbothioamide* (**2d**). Colorless crystal (methanol), 1.18 g (86%), m.p. 162–164 °C, ^1^H-NMR (400 MHz, DMSO-*d_6_*, ppm) δ = 9.01 (s, 1H, NHNHCS), 8.65 (s, 1H, NHCO), 7.74 (s, 1H, NHCS), 6.96 (d, *J* = 2.0 Hz, 1H, PC-H), 6.72 (t, *J* = 7.8, 1.9 Hz, 2H, PC-H), 6.66–6.22 (m, 4H, PC-H), 5.97–5.87 (m, 1H, allyl-CH=), 5.09–5.05 (m, 1H, allyl-CH_2_=), 4.32–4.24 (m, 2H, allyl-CH_2_) 3.84–3.74 (m, 1H, PC-CH_2_-2′), 3.28–2.78 (m, 7H, PC-CH_2_-CH_2_). ^13^C-NMR (100 MHz, DMSO-*d_6_*, ppm) δ = 182.7 (C=S), 167.7 (C=O), 140.1 (PC-C-6′), 139.5 (PC-C-11′), 139.3 (PC-C-14′), 139.0 (PC-C-3′), 135.7, 135.2, 135.1 (PC-CH), 134.8 (allyl-CH=), 132.6, 132.5, 132.3 (PC-CH), 131.5 (PC-CH), 128.9 (PC-C-4′) 115.0 (allyl-CH_2_=), 56.0 (allyl-CH_2_), 34.8 (PC-CH_2_-1′), 34.7 (PC-CH_2_-10′), 34.5 (PC-2CH_2_-9′,2′). IR (ATR, cm^−1^) *ṽ* = 3360 (s, NH), 3132 (m, Ar-CH), 2970 (w, Aliph-CH), 1660 (s, CO), 1380 (s, C=S), 982 (s, C-N str). MS (FAB) *m*/*z* (%) = 366.2 ([M + H]^+^ (100). HRMS ([M + H]^+^, C_21_H_24_O_1_N_3_^32^S_1_) Calcd.: 366.1640, Found: 366.1638.

*2-(4′-[2.2]Paracyclophanyl)-N-ethylhydrazine-1-carbothioamide* (**2e**). Colorless crystal (ethanol/cyclohexane), 1.08 g (81%), m.p. 130–132 °C, ^1^H-NMR (400 MHz, DMSO-*d*_6_, ppm) δ = 9.65 (s, 1H, NHNHCS), 9.22 (s, 1H, NH-CO), 7.81 (s, 1H, NHCS), 6.91 (d, *J* = 1.8 Hz, 1H, PC-H), 6.63 (dd, *J* = 7.7 Hz, *J* = 1.9 Hz, 2H, PC-H), 6.51 (d, *J* = 9.6 Hz, 3H, PC-H), 6.44 (d, *J* = 7.8 Hz, 1H, PC-H), 3.69 (m, 1H, PC-CH_2_-2′), 3.60–3.26 (m, 2H, ethyl-CH_2_), 3.26–2.68 (m, 7H, PC-CH_2_-CH_2_), 1.06 (t, *J* = 10.1 Hz, *J* = 7.0 Hz, 3H, ethyl-CH_3_). ^13^C-NMR (100 MHz, DMSO-*d*_6_, ppm) δ = 181.7 (C=S), 167.6 (C=O), 140.1 (PC-C-6′), 139.5 (PC-C-11′), 139.2 (PC-C-14′), 139.0 (PC-C-3′), 135.7 (PC-2CH), 135.1 (PC-CH), 132.7, 132.6, 132.5, 132.4 (PC-CH), 131.5 (PC-C-4′), 56.0 (ethyl-CH_2_), 35.0 (PC-CH_2_-1′), 34.9 (PC-CH_2_-10′), 34.6 (PC-2CH_2_-9′,2′), 14.7 (ethyl-CH_3_). IR (ATR, cm^−1^) *ṽ* = 3357 (m, NH), 3190 (m, Ar-CH), 2870 (w, Aliph-CH), 1667 (s, CO), 1365 (s, C=S), 980 (s, C-N str). MS (FAB) *m*/*z* (%) = 354.2 ([M + H]^+^ (100). HRMS ([M + H]^+^, C_21_H_24_O_1_N_3_^32^S_1_) Calcd.: 354.1640, Found: 354.1640.

*2-(4′-[2.2]Paracyclophanyl)-N-cyclopropylhydrazine-1-carbothioamide* (**2f**). Colorless crystal (ethanol), 1.10 g (80%), m.p. 158–160 °C, ^1^H-NMR (400 MHz, DMSO-*d*_6_, ppm) δ = 9.66 (s, 1H, NHNHCS), 9.34 (s, 1H, NHCO), 6.90 (s, 1H, NHCS), 6.61 (dd, *J* = 7.7, 1.8 Hz, 2H, PC-H), 6.55–6.46 (m, 4H, PC-H), 6.43 (d, *J* = 7.8 Hz, 1H, PC-H), 3.74–3.43 (m, 1H, PC-CH_2_-CH_2_), 3.38 (d, *J* = 38.4 Hz, 2H, PC-CH_2_-CH_2_), 3.09–2.95 (m, 2H, PC-CH_2_-CH_2_), 2.94–2.80 (m, 3H, PC-CH_2_-CH_2_), 1.23–1.06 (m, 1H, cyclopropyl-CH), 1.05–1.03 (m, *J* = 7.0 Hz, 2H, cyclopropyl-CH_2_), 0.63–0.56 (m, *J* = 52.5 Hz, 2H, cyclopropyl-CH_2_). ^13^C-NMR (100 MHz, DMSO-*d*_6_, ppm) δ = 190.6 (C=S), 167.2 (C=O), 140.0 (PC-C-6′), 139.4 (PC-C-11′), 139.2 (PC-C-14′), 138.9 (PC-C-3′), 135.6 (PC-2CH), 135.0, 132.5, 132.4 (PC-CH), 132.3 (PC-2CH), 131.6 (PC-C-4′), 34.7 (PC-CH_2_-1′), 34.6 (PC-CH_2_-10′), 34.5 (PC-CH_2_-9′), 34.4 (PC-CH_2_-2′), 27.1 (cyclopropyl-CH), 6.7 (cyclopropyl-CH_2_), 6.5 (cyclopropyl-CH_2_). IR (ATR, cm^−1^) *ṽ* = 3208 (s, NH), 3008 (w, Ar-CH), 2919 (s, aliph-CH), 1672 (s, CO), 10 (s, C=S), 978 (s, C-N str). MS (FAB) *m*/*z* (%) = 366.2 ([M + H]^+^ (85). HRMS ([M + H]^+^, C_21_H_24_O_1_N_3_^32^S_1_) Calcd.: 366.1640, Found: 366.1639.

#### 3.1.3. Reactions of Hydrazinecarbothioamide Derivatives **2a**–**f** with **9**: Preparation of Compounds **3a**–**f**

A mixture of hydrazinecarbothioamide derivatives (**2a**–**f**, 1 mmol) and **9** (0.142 g, 1 mmol) in 40 mL of absolute methanol was refluxed for 3–4 h (the reaction was monitored by thin-layer chromatography). After removal of the solvent on vacuum, the crude residue was purified by column chromatography (cyclohexane/ethyl acetate 10:6) as eluent to afford **3a**–**f**. 

*Methyl (E)-2-((E)-2-(2-(4′-[2.2]paracyclophanyl)hydrazinylidene)-4-oxo-3-phenylthiazolidin-5-ylidene)acetate* (**3a**). Colorless crystal (ethanol), 0.20 g (78%), m.p. 202–204 °C, ^1^H-NMR (400 MHz, DMSO-*d*_6_, ppm) δ = 10.77 (s, 1H, NH), 7.57 (d, *J* = 7.5 Hz, 2H, Ph-H), 7.5–7.41 (m, 2H, Ph-H), 7.16–7.00 (m, 1H, Ph-H), 7.05–6.97 (m, 1H,PC-H), 6.86 (s, 1H, vinyl-CH), 6.80–6.67 (m, 2H, PC-H), 6.65–6.41 (m, 4H, PC-H), 3.87 (s, 3H, OCH_3_), 3.14–3.09 (m, 3H, PC-CH_2_-CH_2_), 3.05–2.94 (m, 2H, PC-CH_2_-CH_2_), 2.93–2.81 (m, 3H, PC-CH_2_-CH_2_). ^13^C-NMR (100 MHz, DMSO-*d*_6_, ppm) δ = 165.8 (ester-CO), 165.5 (cyclic-CO), 165.4 (hydrazide-CO), 140.9 (thiazolidine-C-2), 139.6 (thiazolidine-C-5), 139.5 (PC-C-6′), 139.3 (PC-C-11′), 139.0 (PC-C-14′), 138.9 (PC-C-3′), 137.6 (Ph-C), 135.8, 135.6, 134.0 (PC-CH), 132.6, 132.4, 132.2, 132.1 (PC-CH), 131.2, 129.6, 129.5, 129.0 (Ph-CH), 128.1 (Ph-CH), 125.3 (PC-C-4′), 117.5 (vinyl-CH), 52.8 (OCH_3_), 34.7 (PC-CH_2_-1′), 34.5 (PC-CH_2_-10′), 34.3 (PC-CH_2_-9′), 34.1 (PC-CH_2_-2′). IR (ATR, cm^−1^) *ṽ* = 38 (s, NH), 3190 (w, Ar-CH), 2927 (m, aliph-CH), 1724, 1704, 1649 (s, CO), 1613 (s, C═N), 1587 (m, Ar-C═C), 980 (s, C-N str). MS (FAB) *m*/*z* (%) = 512.2 ([M + H]^+^ (100). HRMS ([M + H]^+^, C_29_H_26_O_4_N_3_^32^S_1_) Calcd.: 512.1644, Found: 512.1645.

*Methyl (E)-2-((E)-2-(2-(4′-[2.2]paracyclophanyl)hydrazinylidene)-3-benzyl-4-oxothiazolidin-5-ylidene)acetate* (**3b**). Colorless crystal (ethanol/cyclohexane), 0.20 g (76%), m.p. 175–177 °C, ^1^H-NMR (400 MHz, DMSO-*d*_6_, ppm) δ = 10.88 (s, 1H, NH), 7.43–7.31 (m, 3H, Ph-H), 7.28–7.23 (m, 1H, Ph-H), 6.99 (d, *J* = 4.8 Hz, 1H, Ph-H), 6.93 (s, 1H, vinyl-CH), 6.88–6.82 (m, 1H, PC-H), 6.71–6.55 (m, 2H, PC-H), 6.45 (d, *J* = 10.3 Hz, 2H, PC-H), 6.28 (d, *J* = 2.5 Hz, 2H, PC-H), 5.44 (s, 2H, benzyl-CH_2_), 3.83 (s, 3H, OCH_3_), 3.68–3.62 (m, 2H, PC-CH_2_-CH_2_), 3.18–3.05 (m, 2H, PC-CH_2_-CH_2_), 3.02–2.82 (m, 4H, PC-CH_2_-CH_2_). ^13^C-NMR (100 MHz, DMSO-*d*_6_, ppm) δ = 166.0 (ester-CO), 165.6 (cyclic-CO), 161.5 (hydrazide-CO), 146.2 (thiazolidine-C-2), 140.2 (thiazolidine-C-5), 139.6 (PC-C-6′), 139.3 (PC-C-11′), 139.2 (PC-C-14′), 139.1 (PC-C-3′), 139.0 (Ph-C), 138.9, 138.6, 138.4, 137.8 (PC-CH), 136.0, 135.9, 135.8 (PC-CH), 132.5, 132.3, 132.2, 131.4 (Ph-CH), 128.5 (Ph-CH), 127.3 (PC-C-4′), 116.6 (vinyl-CH), 52.6 (OCH_3_), 51.3 (benzyl-CH_2_), 34.8 (PC-CH_2_-1′), 34.6 (PC-CH_2_-10′), 34.5 (PC-CH_2_-9′), 34.4 (PC-CH_2_-2′). IR (ATR, cm^−1^) *ṽ* = 3390 (m, NH), 3091 (m, Ar-CH), 2927 (m, aliph-CH), 1749, 1695, 1659 (s, CO), 1626 (s, C═N), 1595 (m, Ar-C═C), 980 (s, C-N str). MS (FAB) *m*/*z* (%) = 526.2 ([M + H]^+^ (60). HRMS ([M + H]^+^, C_30_H_28_N_3_O_4_^32^S_1_) Calcd.: 526.1801, Found: 526.1799.

*Methyl (E)-2-((E)-3-[2.2]paracyclophanyl)amido-4-oxo-2-(pyridin-3-ylimino)-thiazolidin-5-ylidene)acetate* (**3c**). Colorless crystal (ethanol), 0.17 g (65%), m.p. 262–264 (decomp) °C, ^1^H-NMR (400 MHz, Acetone-*d*_6_, ppm) δ = 10.12 (s, 1H, NH), 8.43–8.28 (m, 2H, Pyr-H), 7.48–7.44 (m, 2H, Pyr-H), 7.05–6.98 (m, 2H, PC-H), 6.86–6.74 (m, 2H, PC-H), 6.65–6.62 (m, 1H, PC-H), 6.57 (s, 1H, vinyl-CH), 6.54–6.43 (m, 2H, PC-H), 4.00–3.90 (m, 1H, PC-CH_2_-CH_2_), 3.82 (s, 3H, OCH_3_), 3.20–2.91 (m, 7H, PC-CH_2_-CH_2_). ^13^C-NMR (100 MHz, Acetone-*d*_6_, ppm) δ = 167.0 (ester-CO), 166.6 (cyclic-CO), 162.5 (hydrazide-CO), 151.3 (thiazolidine-C-2), 147.5, 144.3 (Pyr-CH), 143.1 (Pyr-C), 141.1 (thiazolidine-C-5), 140.7 (PC-C-6′), 140.2 (PC-C-14′), 138.9 (PC-C-11′), 137.1 (PC-C-3′), 137.0 (PC-3CH), 133.7 (PC-2CH), 133.4, 133.2 (PC-CH), 132.6 (PC-C-4′), 128.8 (Pyr-CH), 125.0 (Pyr-CH), 118.6 (vinyl-CH), 53.3 (OCH_3_), 35.8 (PC-CH_2_-1′), 35.7 (PC-CH_2_-10′), 35.6 (PC-CH_2_-9′), 35.5 (PC-CH_2_-2′). IR (ATR, cm^−1^) *ṽ* = 3187 (w, NH), 2924 (m, Ar-CH), 2852 (w, aliph-CH), 1727, 1693, 1649 (s, CO), 1602 (s, C═N), 1480 (s, Ar-C═C), 980 (s, C-N str). MS (FAB) *m*/*z* (%) = 513.2 ([M + H]^+^ (50). HRMS ([M + H]^+^, C_28_H_25_O_4_N_4_^32^S_1_) Calcd.: 513.1597, Found: 513.1597.

*Methyl (E)-2-((E)-3-allyl-2-(2-(4′-[2.2]paracyclophanyl)hydrazinylidene)-4-oxothiazolidin-5-ylidene)acetate* (**3d**). Colorless crystal (ethanol), 0.17 g (79%), m.p. 241–243 °C, ^1^H-NMR (400 MHz, Acetone-*d*_6_, ppm) δ = 9.78 (s, 1H, NH), 7.08–6.88 (m, 2H, PC-H), 6.82 (s, 1H, Vinyl-CH), 6.79–6.68 (m, 2H, PC-H), 6.63–6.57 (m, 2H, PC-CH), 6.50 (d, *J* = 8.1 Hz, 1H, PC-H), 6.03–5.94 (m, 1H, allyl-CH=), 5.38–5.13 (m, 2H, allyl-CH_2_=), 4.55–4.43 (m, 2H, allyl-CH_2_), 4.28–4015 (m, 1H, PC-CH_2_-CH_2_), 3.87 (s, 3H, OCH_3_), 3.26–3.10 (m, 3H, PC-CH_2_-CH_2_), 3.09–2.99 (m, 3H, PC-CH_2_-CH_2_), 2.96–2.87 (m, 1H, PC-CH_2_-CH_2_). ^13^C-NMR (101 MHz, Acetone-*d*_6_, ppm) δ = 166.5 (ester-CO), 166.2 (cyclic-CO), 162.0 (hydrazide-CO), 141.6 (thiazolidine-C-2), 140.6 (thiazolidine-C-5), 140.2 (PC-C-6′), 139.8 (PC-C-11′), 139.1 (PC-C-14′), 136.6 (PC-C-3′), 136.4, 135.9, 133.3, 133.2, 133.1, 133.0, 132.8 (PC-CH), 132.2 (allyl-CH=), 131.3 (PC-C-4′), 118.0 (allyl-CH_2_=), 116.7 (vinyl-CH), 54.2 (OCH_3_), 52.5 (allyl-CH_2_), 35.6 (PC-CH_2_-1′), 35.3 (PC-CH_2_-10′), 35.2 (PC-CH_2_-9′), 35.1 (PC-CH_2_-2′). IR (ATR, cm^−1^) *ṽ* = 30 (w, NH), 3009 (m, Ar-CH), 2892 (w, aliph-CH), 1703, 1697, 1655 (s, CO), 1601 (s, C═N), 1583 (m, Ar-C═C), 980 (s, C-N str). MS (FAB) *m*/*z* (%) = 476.2 [M + H]^+^ (55). HRMS ([M + H]^+^, C_26_H_26_N_3_O_4_^32^S_1_) Calcd.: 476,1644, Found: 476.1646.

*Methyl (E)-2-((E)-2-(2-(4′-[2.2]paracyclophanyl)hydrazinylidene)-3-ethyl-4-oxothiazolidin-5-ylidene)acetate* (**3e**). Colorless crystal (methanol), m.p. 220–222 °C, 0.16 g (69%), ^1^H-NMR (400 MHz, DMSO-*d*_6_, ppm) δ = 10.83 (s, 1H, NH), 6.80 (s, 1H, vinyl-CH), 6.67 (d, *J* = 7.8 Hz, 3H, PC-H), 6.57 (s, 2H, PC-H), 6.45 (d, *J* = 7.8 Hz, 2H, PC-H), 3.95–2.89 (m, 2H, ethyl-CH_2_), 3.78 (s, 3H, OCH_3_), 3.67–3.59 (m, 1H, PC-CH_2_-CH_2_), 3.16–3.05 (m, 4H, PC-CH_2_-CH_2_), 3.02–2.96 (m, 2H, PC-CH_2_-CH_2_), 2.94–2.87 (m, 1H, PC-CH_2_-CH_2_), 1.26 (t, *J* = 7.1 Hz, 3H, ethyl-CH_3_). ^13^C-NMR (100 MHz, DMSO-*d*_6_, ppm) δ = 165.9 (ester-CO), 164.8 (cyclic-CO), 163.4 (hydrazide-CO), 155.0 (thiazolidine-C-2), 141.0 (thiazolidine-C-5), 139.6 (PC-C-6′), 139.5 (PC-C-11′), 139.2 (PC-C-14′), 139.1 (PC-C-3′), 135.8 (PC-2CH), 135.2 (PC-C-4′), 132.6 (PC-2CH), 132.4, 132.3, 131.4 (PC-CH), 114.9 (vinyl-CH), 52.7 (OCH_3_), 38.1 (ethyl-CH_2_), 34.9 (PC-CH_2_-1′), 34.7 (PC-CH_2_-10′), 34.5 (PC-CH_2_-9′), 34.4 (PC-CH_2_-2′), 12.5 (ethyl-CH_3_). IR (ATR, cm^−1^) *ṽ* = 32 (w, NH), 2931 (m, Ar-CH), 2880 (w, aliph-CH), 1698, 1694, 1648 (s, CO), 1606 (s, C═N), 1545 (s, Ar-C═C), 976 (s, C-N str). MS (FAB) *m*/*z* (%) = 464.2 ([M + H]^+^ (100). HRMS ([M + H]^+^, C_25_H_26_N_3_O_4_^32^S_1_) Calcd.: 464.1644, Found: 464.1643.

*Methyl-(E)-2-((E)-2-(2-(4′-[2.2]paracyclophanyl)hydrazinylidene)-3-cyclopropyl-4-oxothiazolidin-5-ylidene)acetate* (**3f**). Colorless crystal (ethanol), m.p. 180–182 °C, 0.16 g (67%), ^1^H-NMR (400 MHz, DMSO-*d*_6_, ppm) δ = 10.80 (s, 1H, NH), 6.85 (s, 1H, vinyl-CH), 6.75 (s, 1H, PC-H), 6.70–6.65 (m, 2H, PC-H), 6.63–6.52 (m, 3H, PC-H), 6.46 (d, *J* = 7.8 Hz, 1H, PC-H), 3.76 (s, 3H, OCH_3_), 3.74–3.34 (m, 1H, PC-CH_2_-CH_2_), 3.22–3.05 (m, 3H, PC-CH_2_-CH_2_), 3.03–2.96 (m, 2H, PC-CH_2_-CH_2_), 2.98–2.84 (m, 2H, PC-CH_2_-CH_2_), 1.08–1.04 (m, 1H, cyclopropyl-CH), 1.03–0.98 (m, 4H, cyclopropyl-CH_2_). ^13^C-NMR (100 MHz, DMSO-*d*_6_, ppm) δ = 165.9 (ester-CO), 164.9 (cyclic-CO), 164.1 (hydrazide-CO), 157.3 (thiazolidine-C-2), 141.3 (thiazolidine-C-5), 139.6 (PC-C-6′), 139.2 (PC-C-11′), 139.1 (PC-C-14′), 135.8 (PC-C-3′), 135.1, 133.0 (PC-CH), 132.6, 132.4 (PC-2CH), 132.3(PC-C-4′), 131.4 (PC-CH), 114.5 (vinyl-CH), 52.6 (OCH_3_), 34.9 (PC-CH_2_-1′), 34.7 (PC-CH_2_-10′), 34.5 (PC-CH_2_-9′), 34.4 (PC-CH_2_-2′), 25.7 (cyclopropyl-CH), 6.7 (cyclopropyl-2CH_2_). IR (ATR, cm^−1^) *ṽ* = 3223 (w, NH), 3010 (m, Ar-CH), 2891 (w, aliph-CH), 1700, 1697, 1652 (s, CO), 1605 (s, C═N), 1560 (s, Ar-C═C), 980 (s, C-N str). MS (FAB) *m*/*z* (%) = 476.2 ([M + H]^+^ (100). HRMS ([M + H]^+^, C_26_H_26_N_3_O_4_^32^S_1_) Calcd.: 476.1644, Found: 476.1643.

### 3.2. Biology Part

#### NCI Screening Assay

As mentioned previously, the methodology of the NCI procedure for the primary anticancer assay is detailed on their site (http://www.dtp.nci.nih.gov). Briefly, the protocol was performed on 60 human tumor cell lines derived from different nine neoplastic diseases. NCI-60 testing was performed either as a single concentration, which was tested on all 60 cell lines at a single dose of 10^−5^ M, or with a concentration of 5 μg/mL. All of the assays were in accordance with the protocol of the Drug Evaluation Branch, National Cancer Institute, Bethesda, USA. If the results obtained met selection criteria, then the compound was tested again on all 60 cell lines in 5× 10-fold dilutions with the top dose being 10^−4^ M or 150 μg/mL. Detailed methods are described in the Supplementary Materials related to this article.

Other methods of biology items were reported such as the MTT cytotoxicity assay method [41], analysis of cell cycle by flow cytometry [42], caspase assay [43], BAX assay [44], Bcl-2 assay [45], assessment of mitochondrial changes [46], tubulin polymerization inhibitory activity [47], methods of detection of ROS in suspension, and adherent cells by microplate assay [48]. In addition, the methodology dealing with the multidrug resistance assay was reported in Reference [48].

### 3.3. Docking Studies

A docking simulation study is performed using Molecular Operating Environment (MOE^®^) version 2014.09, Chemical Computing Group Inc., Montreal, Canada. The computational software was operated under “Windows XP” installed on an Intel Pentium IV personal computer (PC) with a 1.6-GHz processor and 512 MB of memory. The target compounds were constructed into a three-dimensional (3D) model using the builder interface of the MOE program [49]. 

### 3.4. Crystallographic Structure Analyses 

#### Crystal Structure Determinations of **2a**, **2b**, **2d**, **3b**, and **3c**

The single-crystal X-ray diffraction studies were carried out on a Bruker D8 Venture diffractometer with PhotonII detector at 123(2) K using Cu-Kα radiation (λ = 1.54178 Å). Dual space methods (SHELXT) [50] were used for structure solution, and refinement was carried out using SHELXL-2014 (full-matrix least-squares on *F^2^*) [51]. Hydrogen atoms were localized by difference electron density determination and refined using a riding model (H(N) free). Semi-empirical absorption corrections were applied.

**1**: Colorless crystals, C_17_H_18_N_2_O, *M*_r_ = 266.33, crystal size 0.16 × 0.06 × 0.02 mm, monoclinic, space group *C*2/c (No. 15), *a* = 11.8196(4) Å, *b* = 7.9087(3) Å, *c* = 28.20(10) Å, *β* = 92.708(2)°, *V* = 2636.58(16) Å^3^, *Z* = 8, *ρ* = 1.342 Mg/m^−3^, *µ*(Cu-K_α_) = 0.67 mm^−1^, *F*(000) = 1136, *2θ_max_* = 144.6°, 10,645 measured reflections (2589 independent reflection in the HKLF 5 file, *R*_int_ = 0.000), 191 parameters, 3 restraints *R*_1_ = 0.071 (for 2452 I > 2σ(I)), w*R*_2_ = 0.174 (all data), *S* = 1.16, largest diff. peak/hole = 0.33/−0. e Å^−3^. Refined as a 2-component twin (BASF 0.139(4)). The option TwinRotMat of the program package PLATON [52] was used to create a HKLF 5 file, which was used for the refinement. Therefore, only unique reflections were used for the refinement (Rint = 0.00) (see cif-file for details).

**2a**: Colorless crystals, C_24_H_23_N_3_OS, *M*_r_ = 401.51, crystal size 0.16 × 0.12 × 0.04 mm, monoclinic, space group *P*2_1_/c (No. 14), *a* = 12.2835(4) Å, *b* = 10.5257(3) Å, *c* = 15.5777(5) Å, *β* = 104.805(2)°, *V* = 1947.21(11) Å^3^, *Z* = 4, *ρ* = 1.0 Mg/m^−3^, *µ*(Cu-K_α_) = 1.64 mm^−1^, *F*(000) = 848, *2θ_max_* = 144.8°, 17,562 reflections, of which 3830 were independent (*R*_int_ = 0.032), 271 parameters, 3 restraints, *R*_1_ = 0.039 (for 3480 I > 2σ(I)), w*R*_2_ = 0.107 (all data), *S* = 1.04, largest diff. peak/hole = 0.34/−0.20 e Å^−3^.

**2b**: Colorless crystals, C_25_H_25_N_3_OS, *M*_r_ = 415.54, crystal size 0.28 × 0.06 × 0.03 mm, orthorhombic, space group *P*ccn (No. 56), *a* = 19.4444(6) Å, *b* = 25.2548(7) Å, *c* = 8.7599(2) Å, *V* = 4301.7(2) Å^3^, *Z* = 8, *ρ* = 1.283 Mg/m^−3^, *µ*(Cu-K_α_) = 1.50 mm^−1^, *F*(000) = 1760, *2θ_max_* = 144.4°, 48,155 reflections, of which 4241 were independent (*R*_int_ = 0.049), 283 parameters, 3 restraints, *R*_1_ = 0.038 (for 3846 I > 2σ(I)), w*R*_2_ = 0.096 (all data), *S* = 1.05, largest diff. peak/hole = 0.27/−0.19 e Å^−3^.

**2d**: Colorless crystals, C_21_H_23_N_3_OS, *M*_r_ = 365.48, crystal size 0.18 × 0.04 × 0.02 mm, orthorhombic, space group *P*bca (No. 61), *a* = 19.5761(14) Å, *b* = 8.2709(7) Å, *c* = 24.0176(17) Å, *V* = 3888.7(5) Å^3^, *Z* = 8, *ρ* = 1.249 Mg/m^−3^, *µ*(Cu-K_α_) = 1.58 mm^−1^, *F*(000) = 1552, *2θ_max_* = 144.4°, 24,493 reflections, of which 3815 were independent (*R*_int_ = 0.087), 244 parameters, 198 restraints (a general RIGU restraint was applied, see cif.file for details), *R*_1_ = 0.059 (for 2913 I > 2σ(I)), w*R*_2_ = 0.141 (all data), *S* = 1.043, largest diff. peak/hole = 0.35/−0.27 e Å^−3^.

**3b**: Yellow crystals, C_30_H_27_N_3_O_4_S, *M*_r_ = 525.60, crystal size 0.14 × 0.12 × 0.04 mm, monoclinic, space group *P*2_1_/c (No. 14), *a* = 13.0205(4) Å, *b* = 12.40(4) Å, *c* = 15.9584(5) Å, *β* = 92.8(1)°, *V* = 2574.16(14) Å^3^, *Z* = 4, *ρ* = 1.356 Mg/m^−3^, *µ*(Cu-K_α_) = 1.46 mm^−1^, *F*(000) = 1104, *2θ_max_* = 144.6°, 41,983 reflections, of which 5074 were independent (*R*_int_ = 0.038), 347 parameters, 1 restraint, *R*_1_ = 0.070 (for 4800 I > 2σ(I)), w*R*_2_ = 0.187 (all data), *S* = 1.06, largest diff. peak/hole = 1.13 (due to possible disorder in the [2.2]paracyclophane moiety)/−0.35 e Å^−3^.

**3c**: Colorless crystals, C_28_H_24_N_4_O_4_S ∙ 0.5 CH_3_OH ∙ 0.5 H_2_O, *M*_r_ = 5.60, crystal size 0.15 × 0.09 × 0.03 mm, monoclinic, space group *C*2/c (No. 15), *a* = 19.3130(6) Å, *b* = 12.4233(6) Å, *c* = 21.8993(8) Å, *β* = 90.008(2)°, *V* = 5254.3(4) Å^3^, *Z* = 8, *ρ* = 1.359 Mg/m^−3^, *µ*(Cu-K_α_) = 1.49 mm^−1^, *F*(000) = 2256, *2θ_max_* = 144.4°, 28,186 reflections, of which 5170 were independent (*R*_int_ = 0.041), 338 parameters, 1 restraint, *R*_1_ = 0.043 (for 4649 I > 2σ(I)), w*R*_2_ = 0.110 (all data), *S* = 1.04, largest diff. peak/hole = 0.42/−0.21 e Å^−3^. 

Refinement with the listed atoms show residual electron density due to a heavily disordered methanol and water in four voids around a center of symmetry, which could not be refined with split atoms. Therefore, the option "SQUEEZE" of the program package PLATON [52] was used to create an hkl file taking into account the residual electron density in the void areas. Therefore, the atom list and unit card do not agree (see cif-file for details). CCDC 1,971,268 (**1**), 1,971,269 (**2a**), 1,971,270 (**2b**), 1,971,271 (**2d**), 1,971,272 (**3b**), and 1,971,273 (**3c**) contain the supplementary crystallographic data for this paper. These data can be obtained free of charge from The Cambridge Crystallographic Data Center via www.ccdc.cam.ac.uk/data_request/cif.

## 4. Conclusions

Herein, methyl 2-(2-(4′-[2.2]paracyclophanyl)-hydrazinylidene)-3-substituted-4-oxothiazolidin-5-ylidene)acetates were synthesized and screened against 60 cancer cell lines. Since the designed compounds showed moderate to high activity as anticancer agents, much work was done with the synthesis and biology of paracyclophane heterocyclic compounds in terms of proving the hypothesis that paracyclophane/thiazole conjugates may work through tubulin polymerization inhibition. Results indicated that compound **3a** could be considered a good pharmacophore for further medicinal study.

## Figures and Tables

**Table 1:** Growth inhibition percentage of compounds **3a**–**f** (concentration 10^−5^ M) against different cell lines.

Panel/Cell Line		3a	3b	3c	3d	3e	3f
**Leukemia**	CCRF-CEM	87.04	96.17	31.27	77.08	66.08	41.43
	HL-60(TB)	83.60	105.46	48.64	8.16	11.64	26.38
	K-562	72.95	81.13	20.72	48.13	45.78	30.52
	MOLT-4	96.97	98.91	21.86	64.55	62.85	55.61
	RPMI-8226	**120.89**	147.00	83.01	109.36	**114.28**	49.83
	SR	**115.60**	114.70	74.18	98.21	**113.40**	39.13
**Non-Small-Cell Lung Cancer**	A549/ATCC	30.92	22.95	2.04	5.20	3.00	11.39
	EKVX	41.03	22.30	2.61	9.16	6.44	12.16
	HOP-62	97.00	22.76	4.00	0	0	0
	HOP-92	24.66	24.50	8.14	18.59	18.43	16.16
	NCI-H226	26.22	35.20	7.48	12.45	7.31	9.41
	NCI-H23	59.57	22.64	9.18	11.85	0.40	9.32
	NCI-H322M	31.54	6.70	1.72	2.94	7.75	4.43
	NCI-H460	39.35	6.29	0.75	0	0	1.98
	NCI-H522	58.47	84.06	17.74	39.25	40.31	30.01
**Colon Cancer**	COLO 205	52.26	27.31	0	0	0	4.48
	HCC-2998	96.36	20.49	0	0	0	0
	HCT-116	95.95	50.48	6.35	46.02	50.54	24.59
	HCT-15	99.66	72.16	11.75	33.11	31.60	29.43
	HT29	**113.13**	87.62	7.71	53.99	54.76	23.99
	KM12	91.51	34.90	7.32	18.40	15.01	1.32
	SW-620	92.61	81.83	10.84	55.46	91.21	12.94
**CNS Cancer**	SF-268	27.49	14.95	3.08	0	2.18	2.56
	SF-295	41.00	10.63	5.63	6.42	3.13	8.80
	SF-539	122.50	56.74	11.63	0.33	9.82	0
	SNB-19	60.06	43.08	16.64	15.31	14.43	11.26
	SNB-75	54.29	48.78	0.25	21.92	21.18	24.73
	U251	84.20	45.04	14.66	18.17	15.64	20.27
**Melanoma**	LOX IMVI	127.77	93.79	9.78	21.22	26.51	9.41
	MALME-3M	21.23	11.57	0	7.56	8.06	11.28
	M14	46.88	36.59	7.99	21.14	12.85	19.23
	MDA-MB-435	10.39	8.44	0	0	0.72	8.06
	SK-MEL-2	12.98	14.42	0	2.70	5.78	13.53
	SK-MEL-28	17.54	0	0.86	0	0	10.28
	SK-MEL-5	30.65	17.72	3.64	7.53	7.50	14.50
	UACC-257	30.75	19.02	0.83	8.89	7.26	16.33
	UACC-62	74.85	48.11	10.67	22.84	21.39	17.97
**Ovarian Cancer**	IGROV1	94.03	57.72	15.44	10.76	11.62	7.20
	OVCAR-3	106.25	79.71	4.51	13.56	7.09	15.87
	OVCAR-4	70.61	55.43	7.76	2.93	10.00	10.51
	OVCAR-5	51.75	34.65	0.61	3.90	0	0
	OVCAR-8	59.27	28.00	1.51	11.78	7.42	13.28
	NCI/ADR-RES	68.61	29.61	4.70	10.50	13.46	7.72
	SK-OV-3	0	0	0	0	0	0
**Renal Cancer**	786-0	**100.28**	50.45	8.52	8.77	9.45	14.62
	A498	50.75	53.92	3.59	12.10	6.63	3.39
	ACHN	30.67	34.18	11.02	12.21	10.45	17.78
	CAKI-1	94.65	84.43	4.78	5.23	0	24.49
	SN12C	77.51	38.76	8.87	13.14	4.97	17.07
	TK-10	84.83	4.53	0	0	6.50	0
	UO-31	41.51	54.96	21.86	24.62	21.03	21.73
**Prostate Cancer**	PC-3	135.88	82.23	18.19	51.62	43.28	18.95
	DU-145	49.13	16.79	0	0	0	6.20
**Breast Cancer**	MCF7	76.79	67.26	26.75	48.47	56.29	36.38
	MDA-MB-231/ATCC	67.47	43.16	10.64	2.27	3.95	3.50
	HS 578T	22.06	12.58	0	6.41	1.03	5.01
	BT-549	64.50	89.68	18.41	38.28	7.73	26.81
	T-47D	**138.25**	**106.25**	31.13	25.00	5.77	23.00
	MDA-MB-468	**100.39**	78.91	18.03	23.68	12.74	29.74

**Table 2:** Results of five in vitro doses (concentrations 0.01, 0.1, 1, 10, and 100 µM) tested with nine human cancer types and selectivity for compound **3a.**

Panel	Cell Line	GI_50_			TGI			LC_50_
		Concentration/Cell Line	Subpanel MID^b^	Selectivity Ratio (MID^a^: MID^b^)	Concentration/Cell Line	Subpanel MID^d^	Selectivity Ratio (MID^c^: MID^d^)	
Leukemia	CCRF-CEM	2.71	2.72	0.88	-	81.14	3.85	>100
	HL-60(TB)	2.70			>100			>100
	K-562	3.15			>100			>100
	MOLT-4	3.12			>100			>100
	RPMI-8226	2.15			5.69			>100
	SR	2.46			>100			>100
Non-Small-Cell Lung Cancer	A549/ATCC	2.83	2.74	0.87	8.00	16.05	1.31	>100
	EKVX	1.91			4.23			9.38
	HOP-62	2.68			6.97			6.60
	HOP-92	1.91			9.14			>100
	NCI-H226	4.38			2.55			>100
	NCI-H23	1.95			4.15			8.85
	NCI-H322M	2.02			4.75			1.32
	NCI-H460	2.08			4.29			8.82
	NCI-H522	4.89			>100			>100
Colon Cancer	COLO 205	2.13	1.91	1.24	4.61	3.95	5.34	
	HCC-2998	1.78			3.45			6.68
	HCT-116	1.79			3.66			7.51
	HCT-15	1.87			4.05			8.76
	HT29	1.93			4.07			8.56
	KM12	2.03			4.15			8.51
	SW-620	1.85			3.66			7.23
CNS Cancer	SF-268	3.09	2.71	0.88	1.12	19.48	1.08	>100
	SF-295	2.07			4.19			8.50
	SF-539	1.79			3.32			6.18
	SNB-19	1.95			4.25			9.26
	SNB-75	5.44			>100			>100
	U251	1.89			4.00			8.47
Melanoma	LOX IMVI	1.69	3.77	0.63	3.	36.54	0.58	6.75
	MALME-3M	3.27			2.14			>100
	M14	3.27			1.54			>100
	MDA-MB-435	5.44			100			>100
	SK-MEL-2	2.65			7.76			>100
	SK-MEL-28	2.97			9.87			>100
	SK-MEL-5	7.56			>100			>100
	UACC-257	5.14			>100			>100
	UACC-62	1.96			4.21			9.02
Ovarian Cancer	IGROV1	1.66	2.59	0.92	3.27	18.46	1.14	6.44
	OVCAR-3	1.80			3.61			7.23
	OVCAR-4	3.17			9.88			3.69
	OVCAR-5	1.92			3.84			7.68
	OVCAR-8	3.08			>100			>100
	NCI/ADR-RES	2.92			1.06			>100
	SK-OV-3	3.55			7.53			>100
Renal Cancer	786-0	2.00	1.86	1.28	4.38	16.64	1.27	9.58
	A498	--			8.36			>100
	ACHN	1.78			3.29			6.08
	RXF393	3.74			3.86			>100
	CAKI-1	1.52			3.00			9.78
	SN12C	2.27			5.94			>100
	TK-10	2.03			5.60			2.92
	UO-31	1.53			3.08			6.20
Prostate Cancer	PC-3	1.71	3.41	0.69	3.63	3.58	5.89	7.70
	DU-145	5.11			3.53			>100
Breast Cancer	MCF7	1.87	2.27	1.05	4.95	24.28	0.87	2.25
	MDA-MB-231/ATCC	2.63			7.46			>100
	HS 578T	3.49			>100			>100
	BT-549	2.22			5.35			2.47
	T-47D	1.54			3.65			8.68
	MDA-MB-468	1.84			3.74			7.61
MID^a^	2.38			MID^c^	21.08			

MID^a,c^ = average sensitivity of all cell lines in mM; MID^b,d^ = average sensitivity of all cell lines of a particular subpanel in mM.

**Table 3:** MTT assay for the antiproliferative IC_50_ ± SD (μM) activity of compounds **3a**–**e** and colchicine.

Compound	Cytotoxicity IC_50_ (µM) ^a^	
	RPMI-8226	L.SR
**3a**	1.61 ± 0.0654 ***	1.11 ± 0.06849 ***
**3b**	4.62 ± 0.0459 ***	2.02 ± 0.06875 ***
**3c**	9.96 ± 0.3451 ***	4.84 ± 0.08425 ***
**3d**	17.81 ± 0.0544 ***	22.06 ± 0.06792 ***
**3e**	3.17 ± 0.0489 ***	5.04 ± 0.07908 ***
**Colchicine**	4.05 ± 0.0478 ***	1.81 ± 0.07009 ***

^a^ IC50 = compound concentration required to inhibit tumor cell proliferation by 50%. Data are expressed as the mean ± SD from the dose–response curves of at least three independent experiments. Results significantly different from control at *** *p* < 0.05.

**Table 4:** Inhibition of tubulin polymerization displaying IC_50_ ± standard error of the mean (SEM) (μM) for compounds **3a**–**e** and colchicine as a reference.

Compound	Tubulin Polymerization Inhibition IC_50_ (µM) ^a^
**3a**	4.97 ± 0.11
**3b**	8.38 ± 0.18
**3c**	8.28 ± 0.18
**3d**	14.79 ± 0.32
**3e**	6.61 ± 0.14
**Colchicine**	3.76 ± 0.08

^a^ Tubulin, colchicine, and the tested compound concentrations were 1, 5, and 5 μM, respectively.

**Table 5:** DNA content % using propidium iodide flow cytometry.

Compound	DNA Content %				
	%G0-G1	%S	%G2-M	%Pre G1	Comment
**3a/SR**	31.47	23.79	44.74 ± 0.1747 ***	33.71	cell growth arrest (G2/M)
**Colchicine/SR**	26.58	25.27	48.15 ± 1.000 ***	36.24	cell growth arrest (G2/M)
**cont.SR**	57.23	28.66	14.11 ± 0.1050	1.92	---

Results significantly different from control at *** *p* < 0.05.

**Table 6:** In vitro growth inhibitory effects of **3a** in comparison to colchicine on multidrug-resistant leukemia cell line (MDR cells). Pgp—P-glycoprotein.

Compound Code	IC_50_ ± SEM (ng/mL) (*n* = 3)	
	Pgp-Mediated MDR	Fold Change
**3a**/**SR**	1.285 ± 0.06	1.15
Colchicine/**SR**	1.726 ± 0.05	1.54
Control/**SR**	1.121 ± 0.05	1

**Table 7:** Caspase-3 conc (pg/mL) ± SD and fold change levels for compounds **3a** and colchicine on leukemia SR cell line.

Compound	Caspase 3	
	Conc pg/mL	Fold Change
**3a**/SR	471.2 ± 6.11	8.84
Colchicine/SR	428.9 ± 5.47	8.05
Control/SR	53.28 ± 1.09	1

**Table 8:** Bax and Bcl-2 conc ± SD and fold change levels for compounds **3a** and colchicine on leukemia SR cell line.

Compound	BAX		Bcl2	
	Concentration (pg/mL)	Fold Change	Concentration (ng/mL)	Fold Change
**3a**/SR	359 ± 3.830 ***	7.98	5.045 ± 0.1918 ***	0.59
Colchicine/SR	341.8 ± 4.310 ***	7.60	4.101 ± 0.1441 ***	0.48
Control/SR	44.96 ± 1.270	1	8.569 ± 0.1565	1

Results significantly different from control at *** *p* < 0.05.

**Table 9:** Energy scores for the complexes formed by the tested compounds **3a**–**e** and the reference colchicine in the active site of β-tubulin enzyme (PDB: 3HKC).

Compound	S Score	ΔG (kcal/mol)^a^	Ligand–Receptor Interaction		
			Residue	Type	Length (Å)
**Colchicine**	−5.62	−3.4 −0.5	Glu 71 Mg 601	H–donor metal	3.16 2.61
**3a**	−7.05	−2.2 −0.6	Mg 601 Ala 247	metal pi–H	2.00 4.17
**3b**	−7.16	−0.9 −2.3 −0.9	Asp 245 Arg 2 Glu 71	H–donor H–acceptor pi–H	3.27 3.41 3.98
**3c**	−6.29	−1.8 −0.7 −0.7	Mg 601 Tyr 224 Ala 247	metal pi–H pi–H	2.40 3.79 4.50
**3d**	−6.94	−1.0 −2.1 −0.7	Glu 71 Mg 601 Arg 2	H–donor metal pi–H	3.90 2.51 4.69
**3e**	−6.43	−2.0 −2.2	Gln 11 Mg 601	H–acceptor metal	3.09 2.33

ΔG (kcal/mol)^a^: the binding free energies.

## Supplementary Materials

Supplementary data for this article can be found online.
