# Supplementary material for: New Paracyclophanylthiazoles with Anti-Leukemia Activity: Design, Synthesis, Molecular Docking, and Mechanistic Studies
Source: Molecules. 2020 Jul 7;25(13):3089. doi: 10.3390/molecules25133089 (PMC7411887; doi:10.3390/molecules25133089)
Supplement: Supplementary file 1 [file molecules-25-03089-s001.pdf]

*Article*

# New Paracyclophanylthiazoles with Anti-Leukemia Activity: Design, Synthesis, Molecular Docking, and Mechanistic Studies

**Ashraf A Aly [1,*], Stefan Bräse [2,3,*], Alaa A. Hassan [1], Nasr K. Mohamed [1], Lamiaa E. Abd El-Haleem [1,2], Martin Nieger [4], Nesrin M. Morsy [5] and Elshimaa M. N. Abdelhafez [6]**

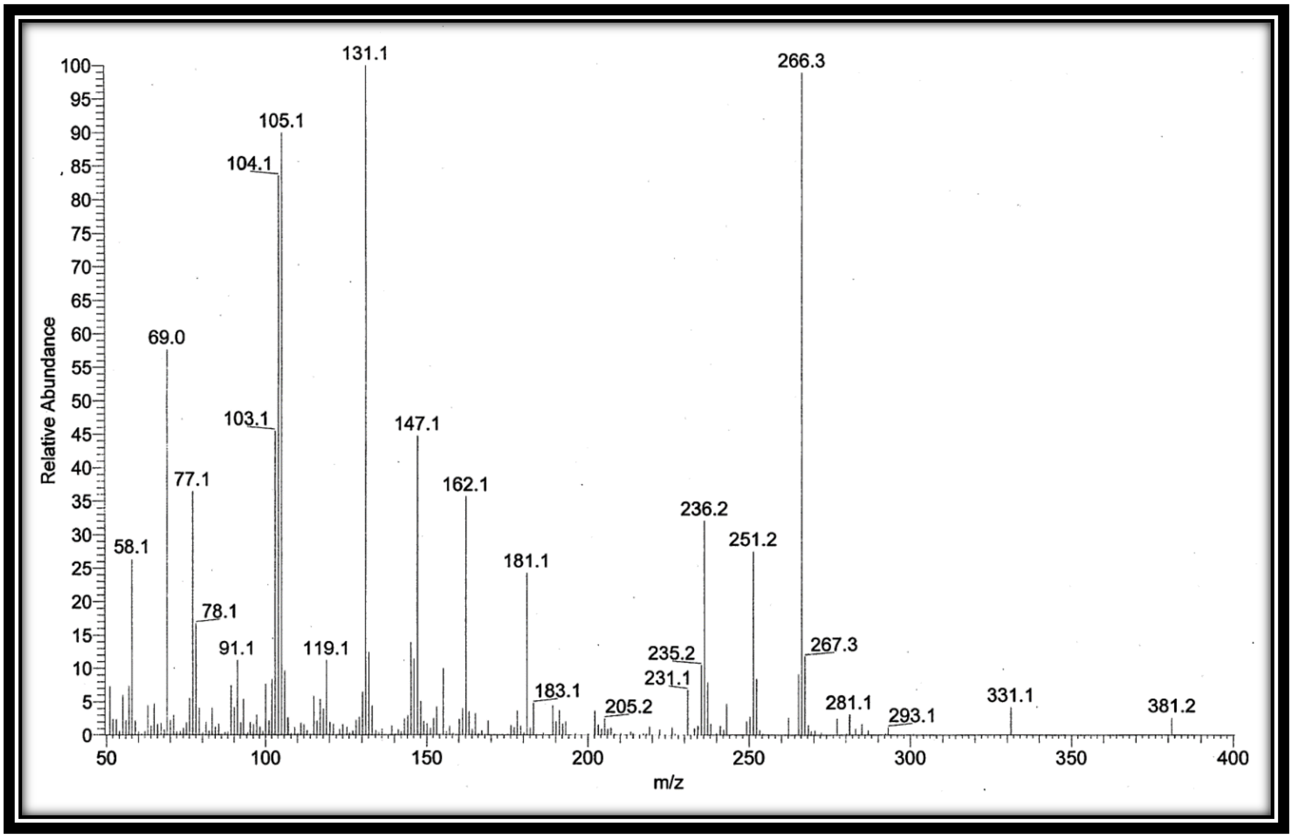


**Figure 1.** Mass spectrum of compound **1**

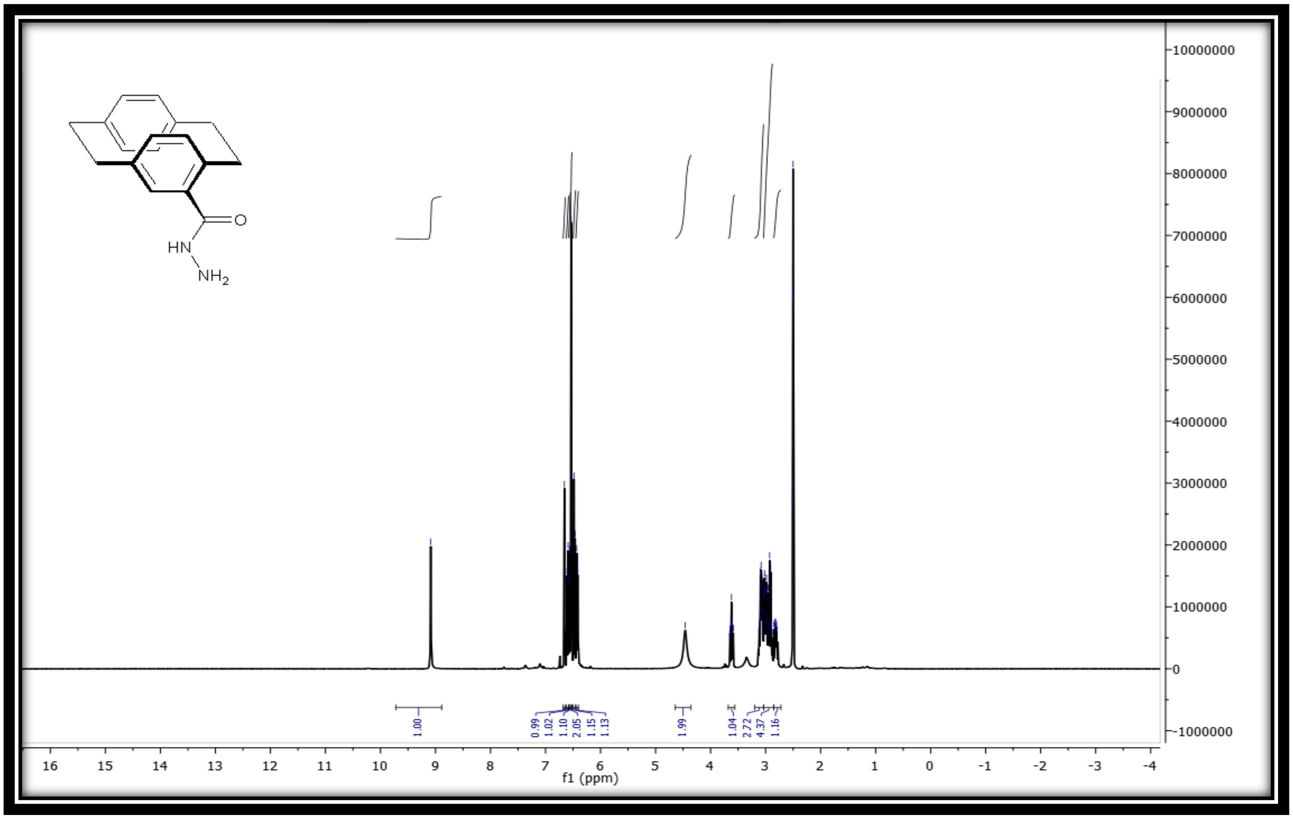

**Figure 2.** $^{1}$H NMR spectrum of compound **1**

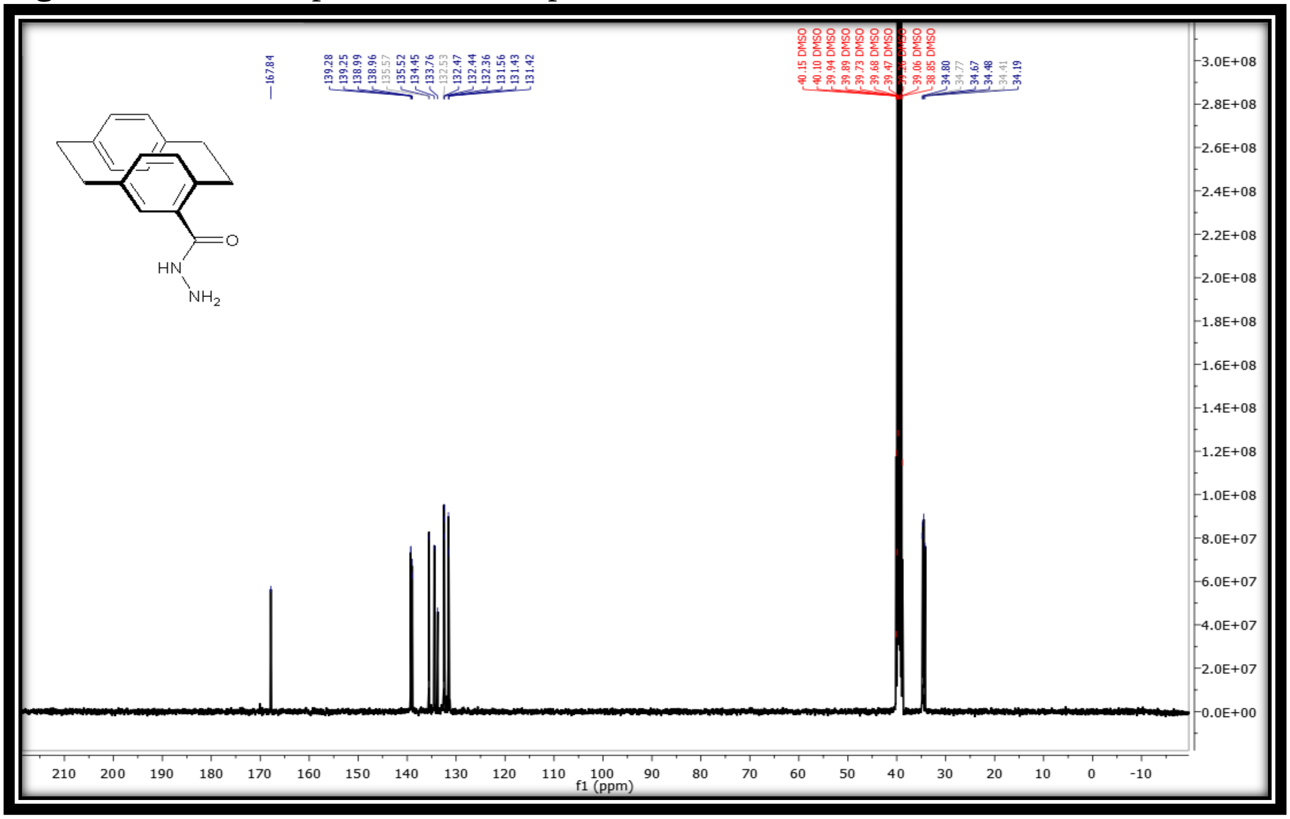

**Figure 3.** $^{13}$C NMR spectrum of compound **1**

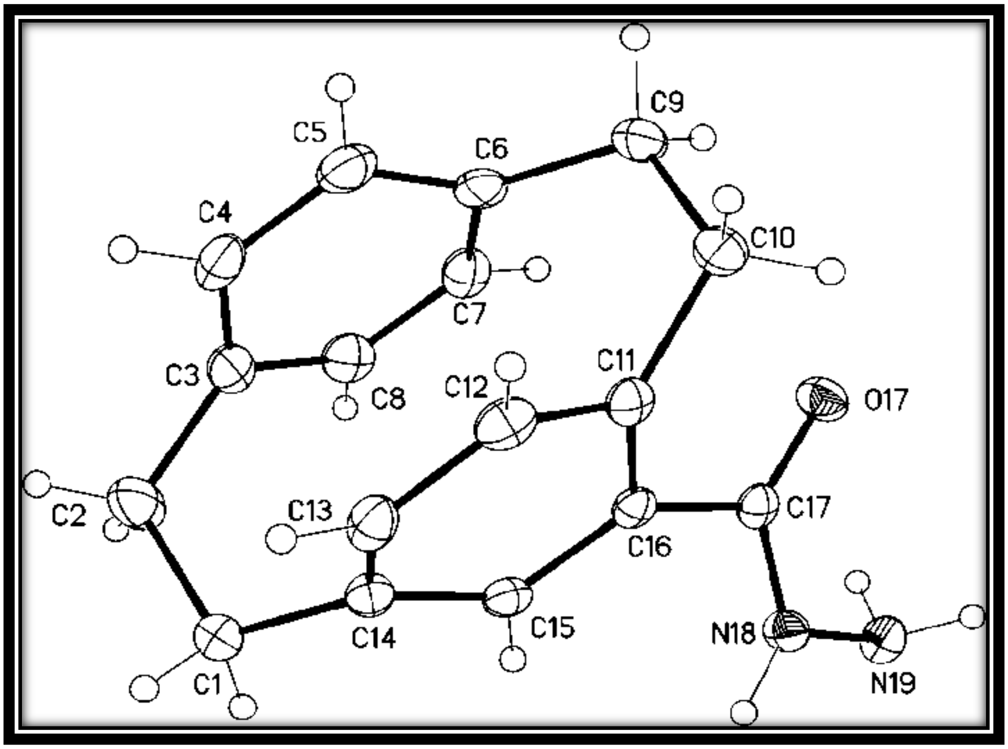

**Figure 4.** Molecular structure of compound **1**

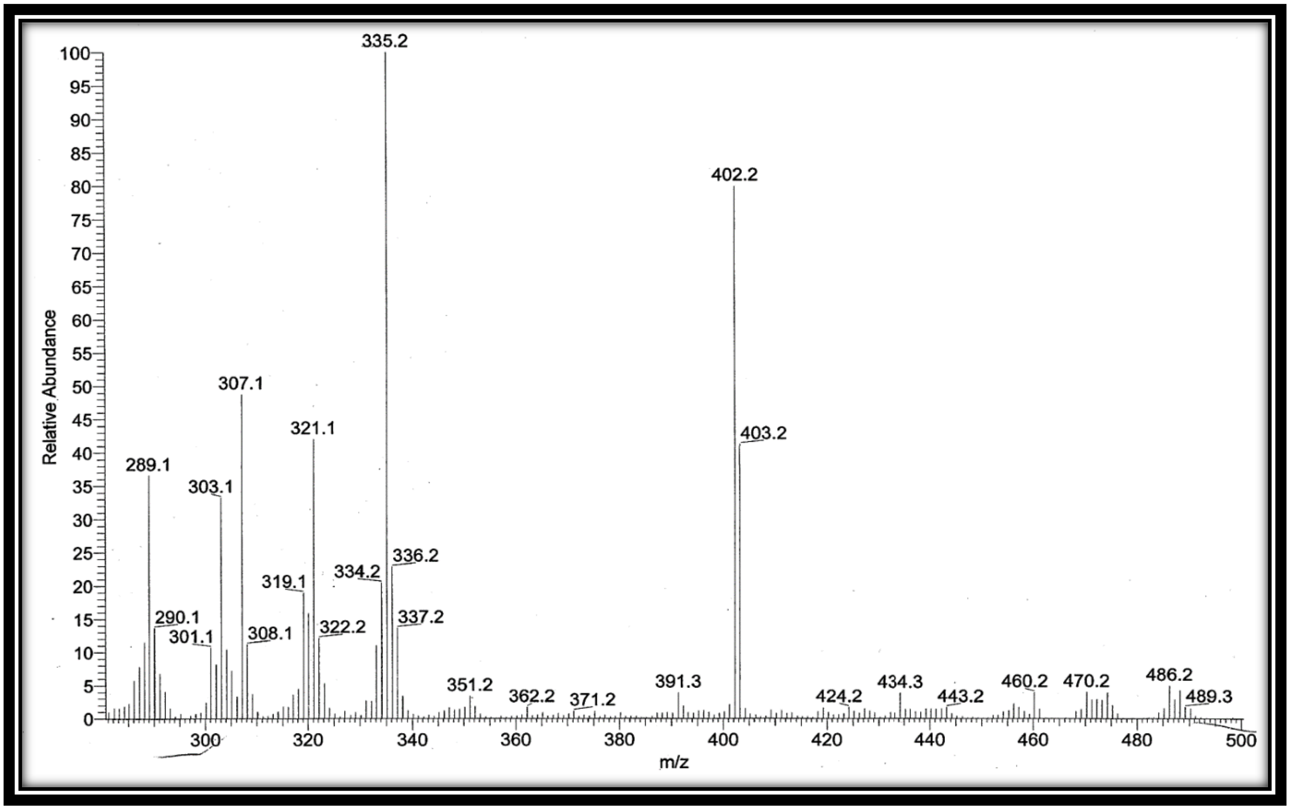

**Figure 5.** Mass spectrum of compound **2a**

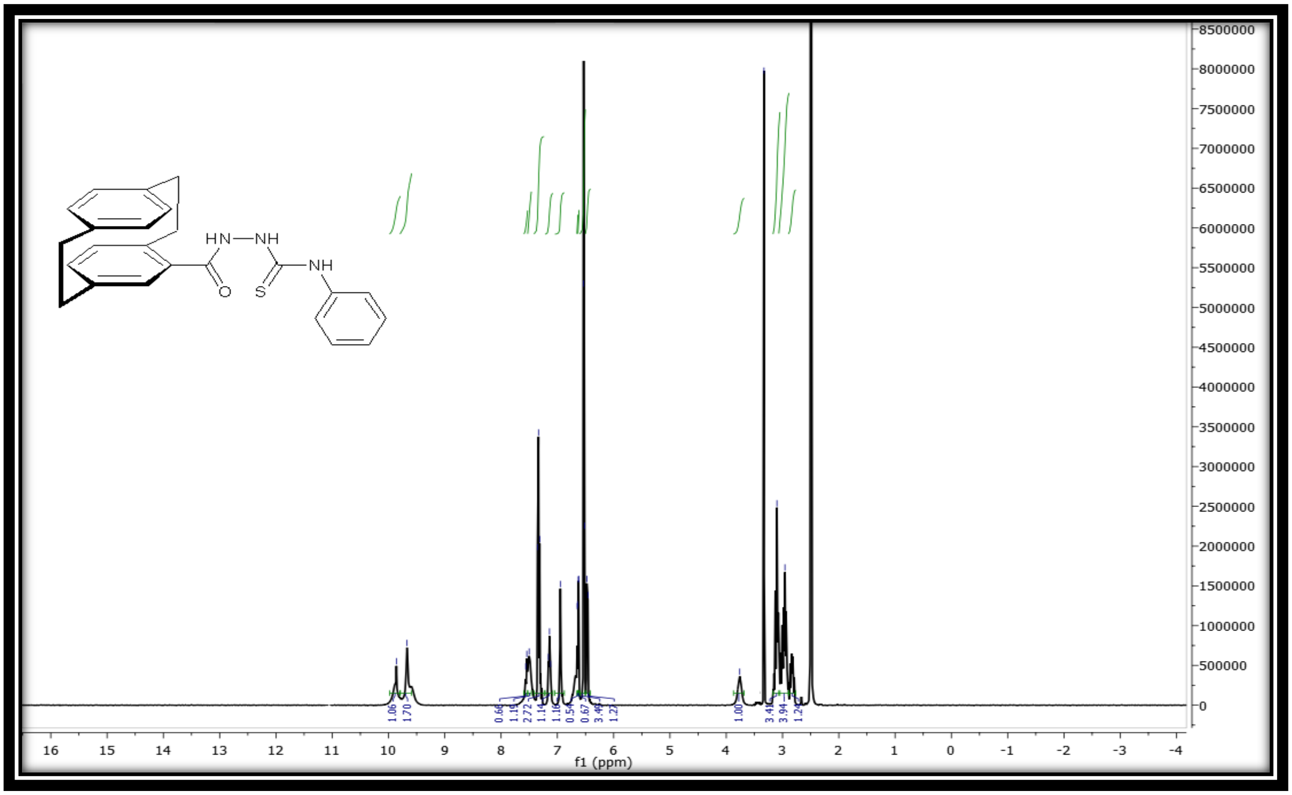

**Figure 6.** $^{1}$H NMR spectrum of compound **2a**

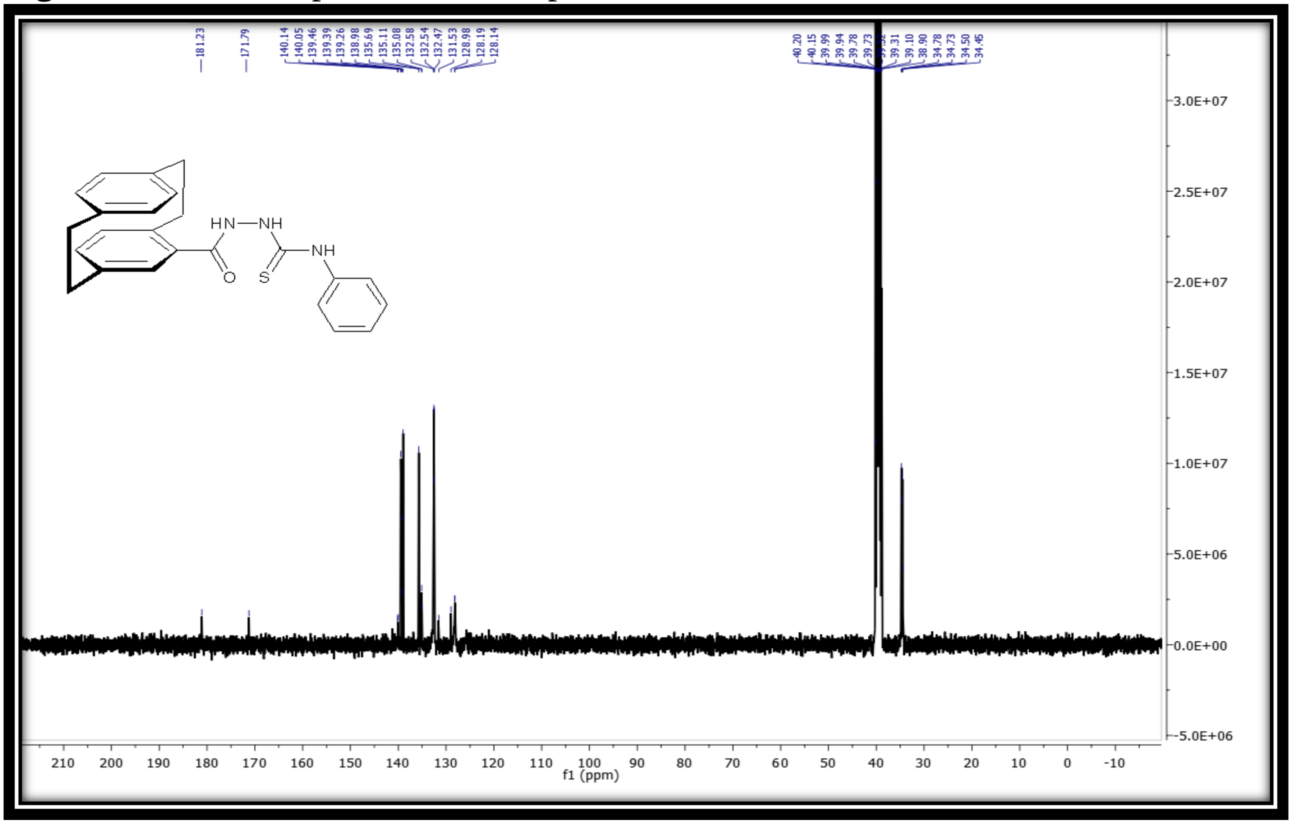

**Figure 7.** $^{13}$**C** NMR spectrum of compound **2a**

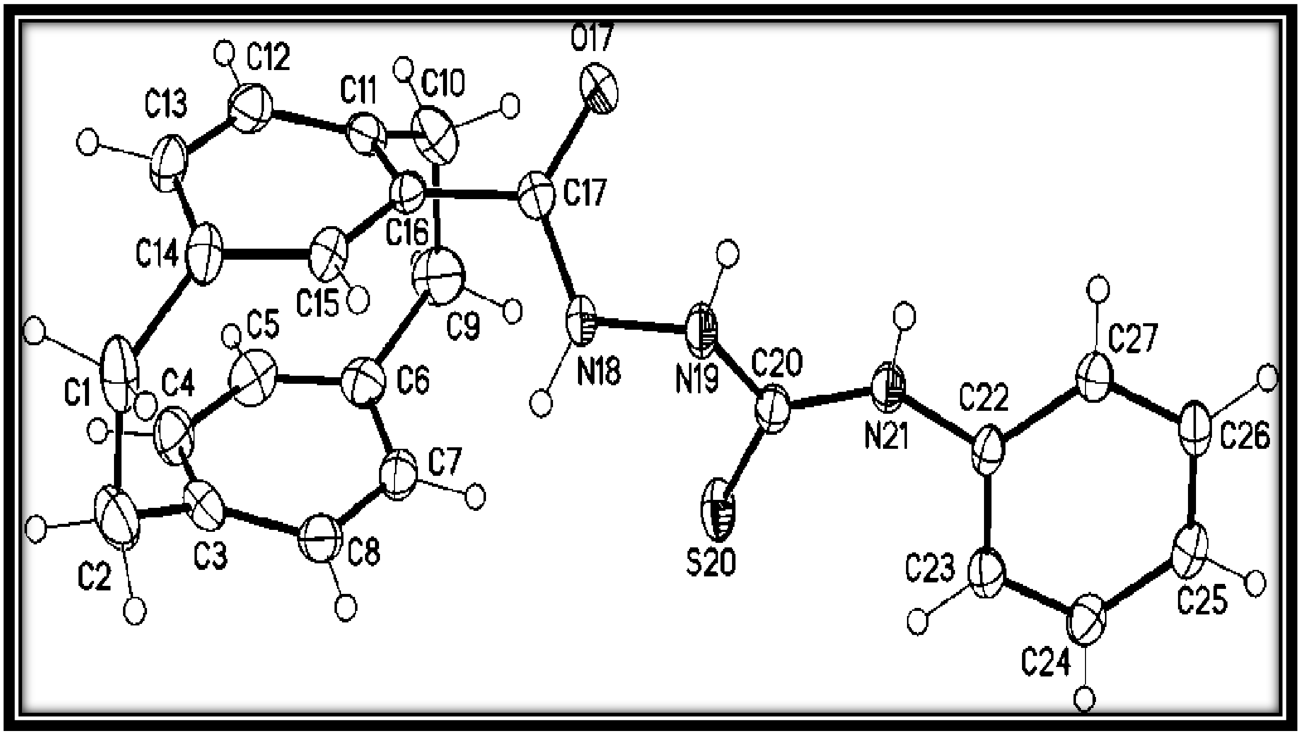

**Figure 8.** Molecular structure of compound **2a**

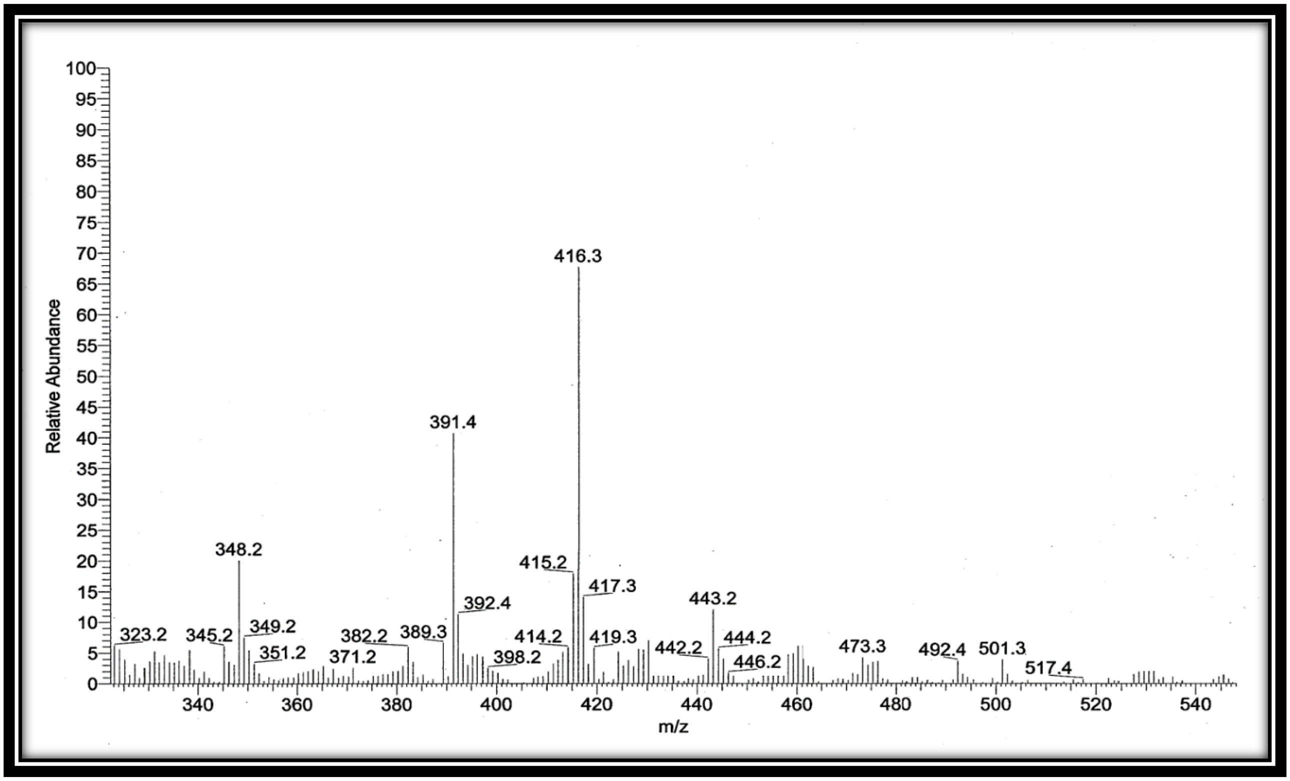

**Figure 9.** Mass spectrum of compound **2b**

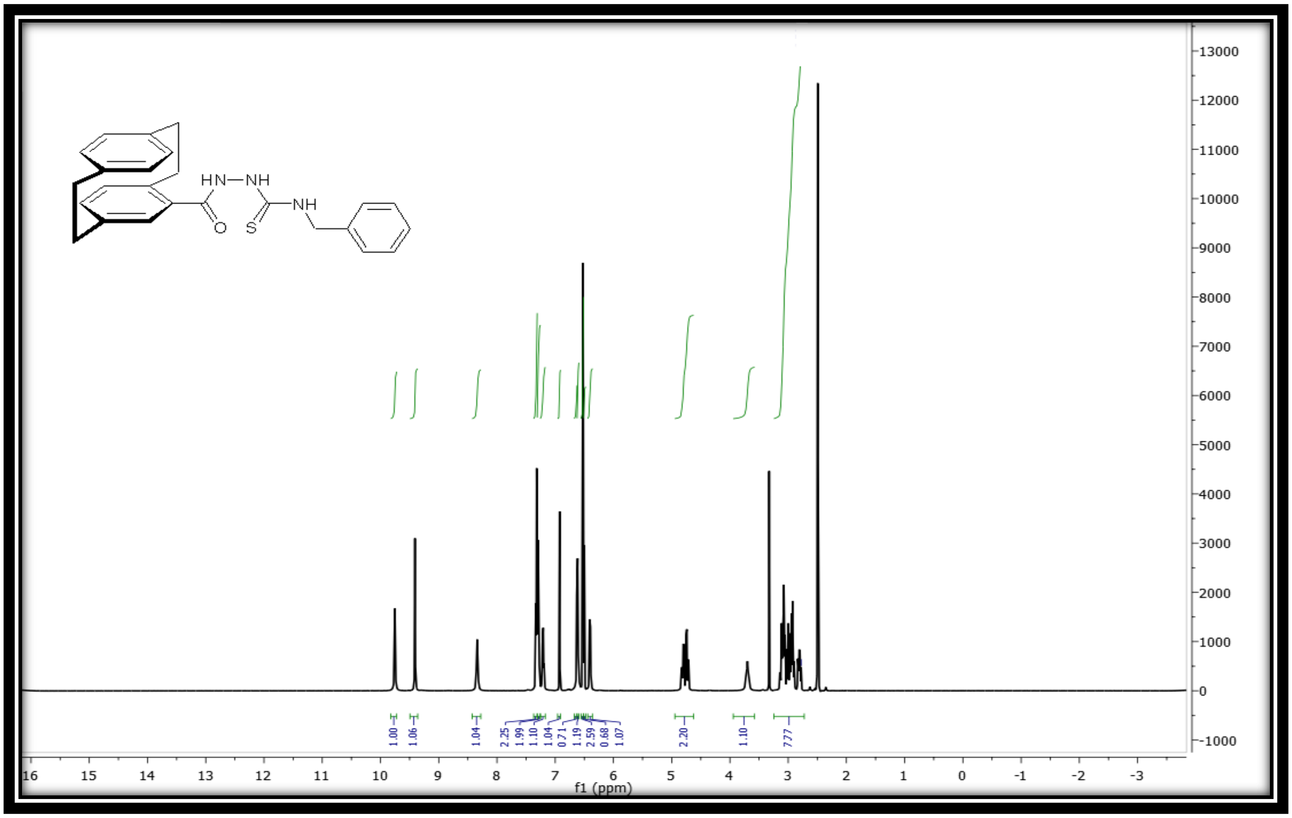


**Figure 10.** $^{1}$H NMR spectrum of compound **2b**

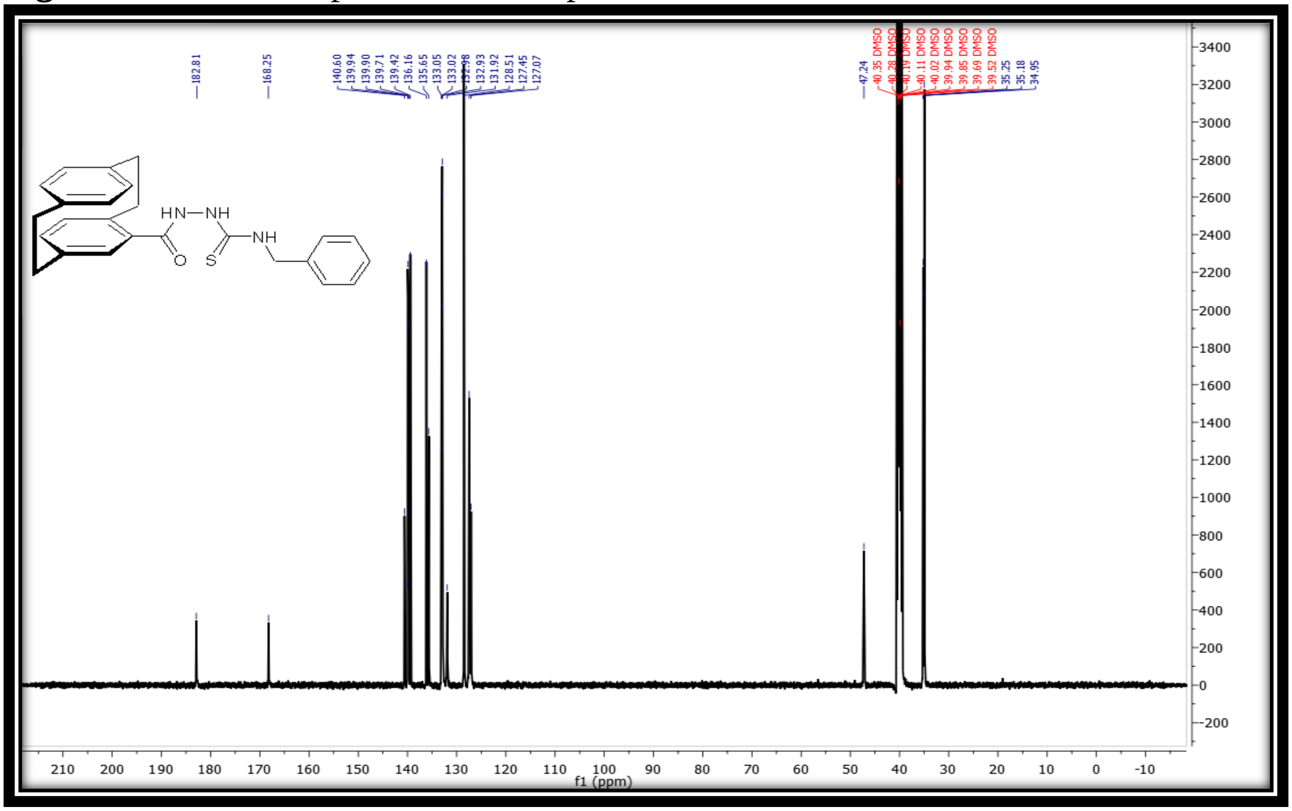


**Figure 11.** $^{13}$C NMR spectrum of compound **2b**

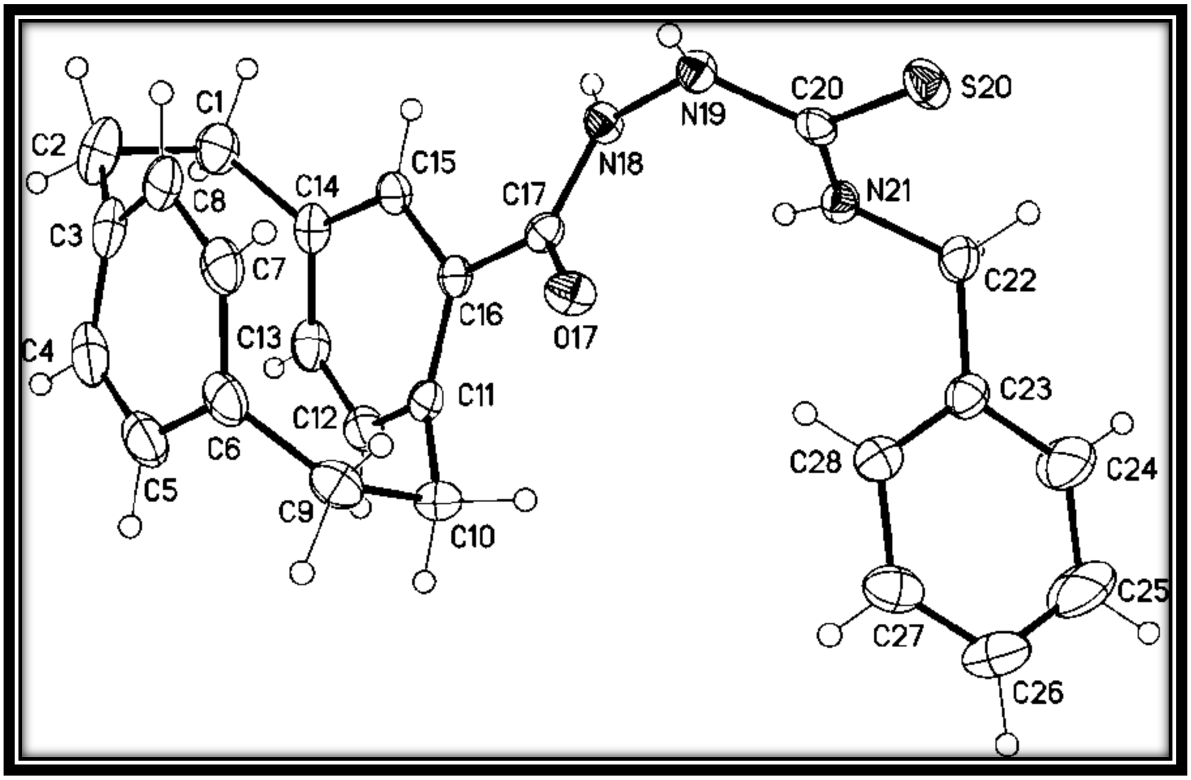


**Figure 12.** Molecular structure of compound **2b**

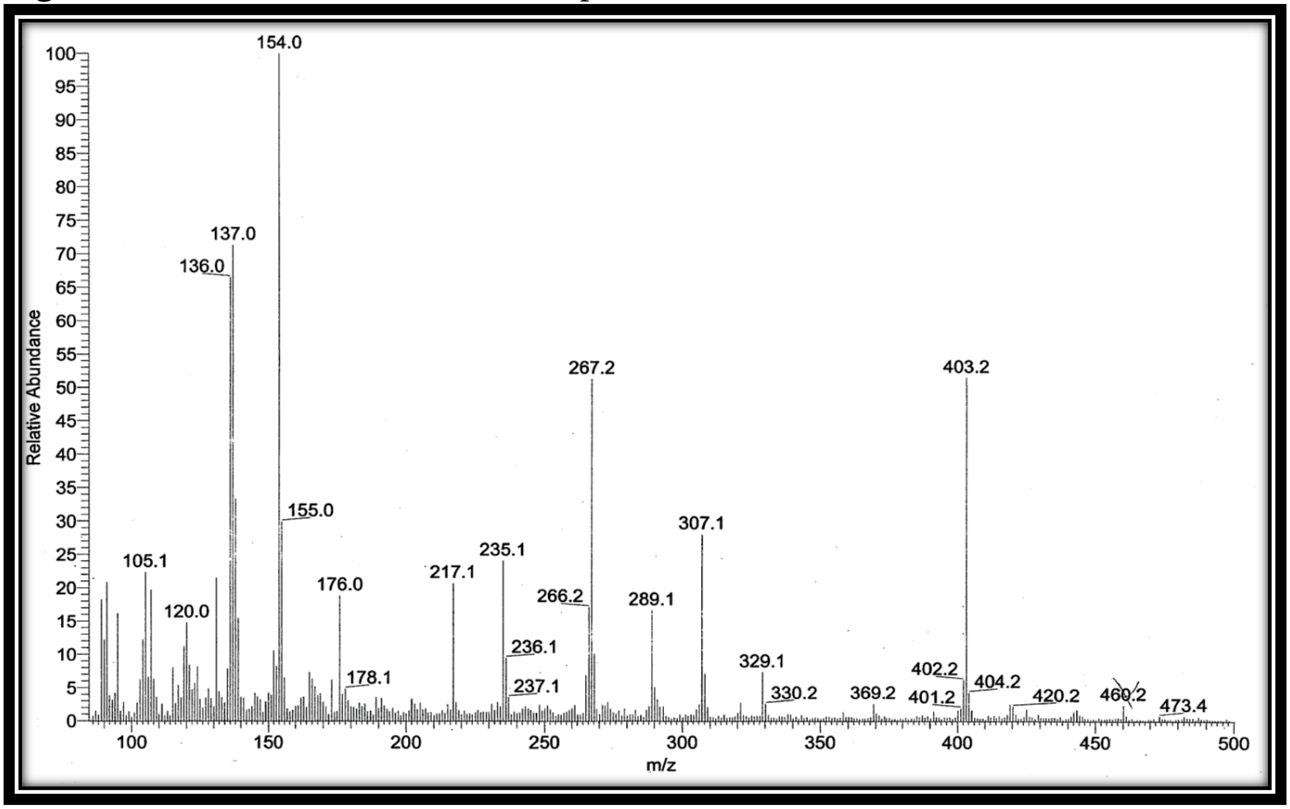


**Figure 13.** Mass spectrum of compound **2c**

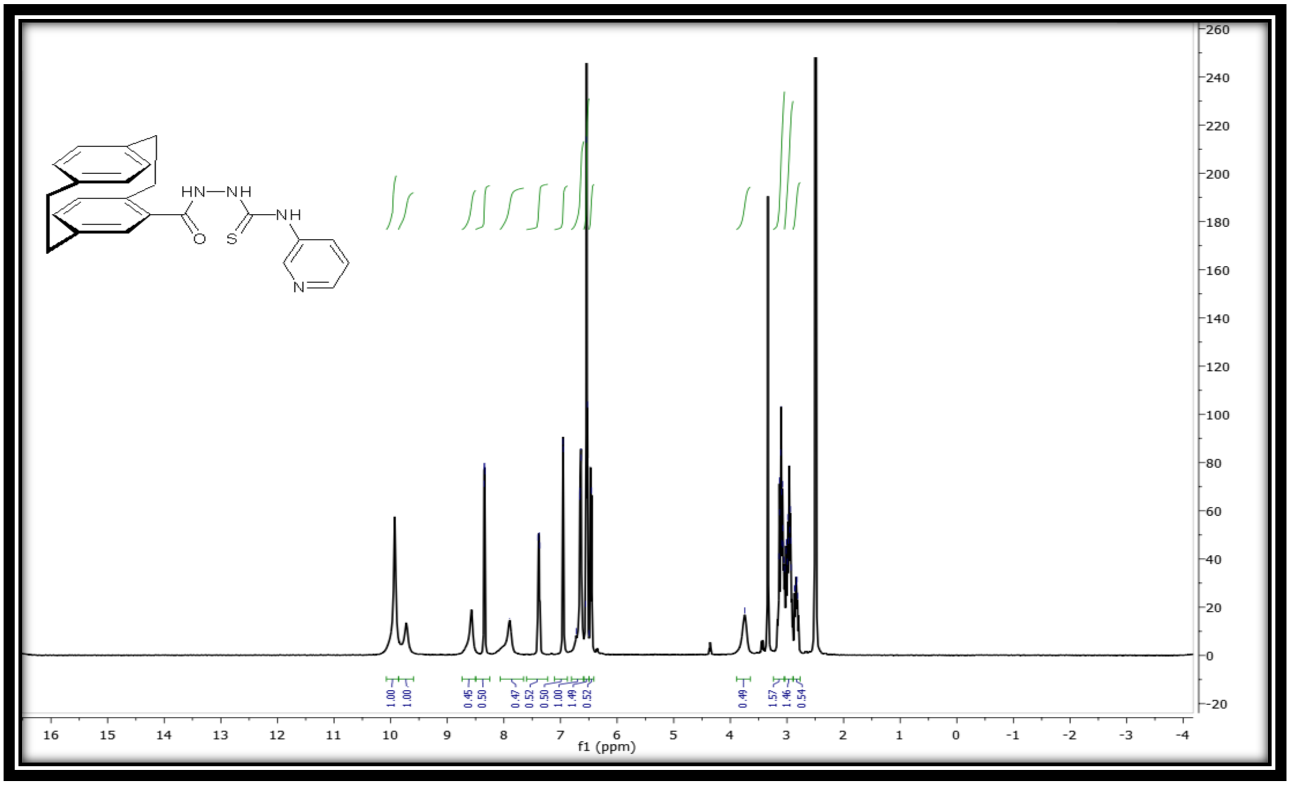


**Figure 14.** $^{1}$H NMR spectrum of compound **2c**

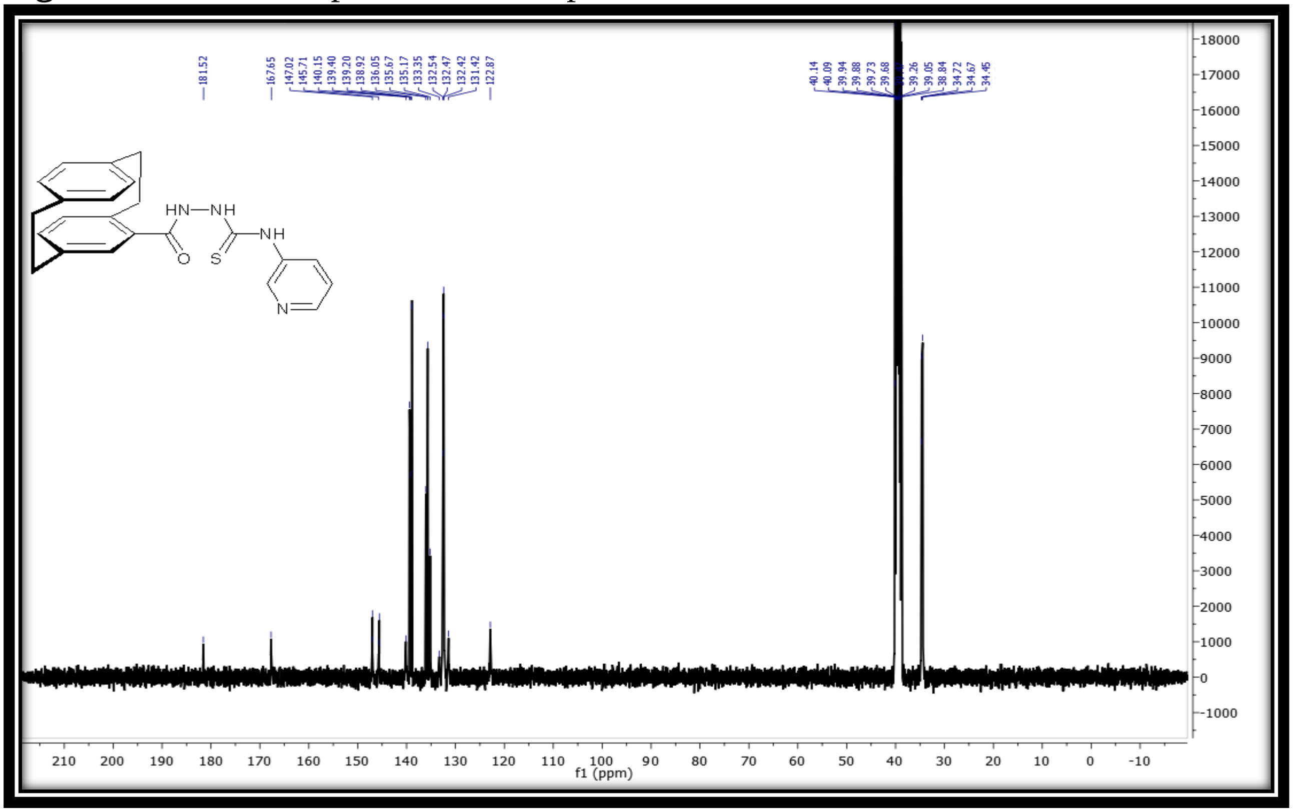


**Figure 15.** $^{13}$**C** NMR spectrum of compound **2c**

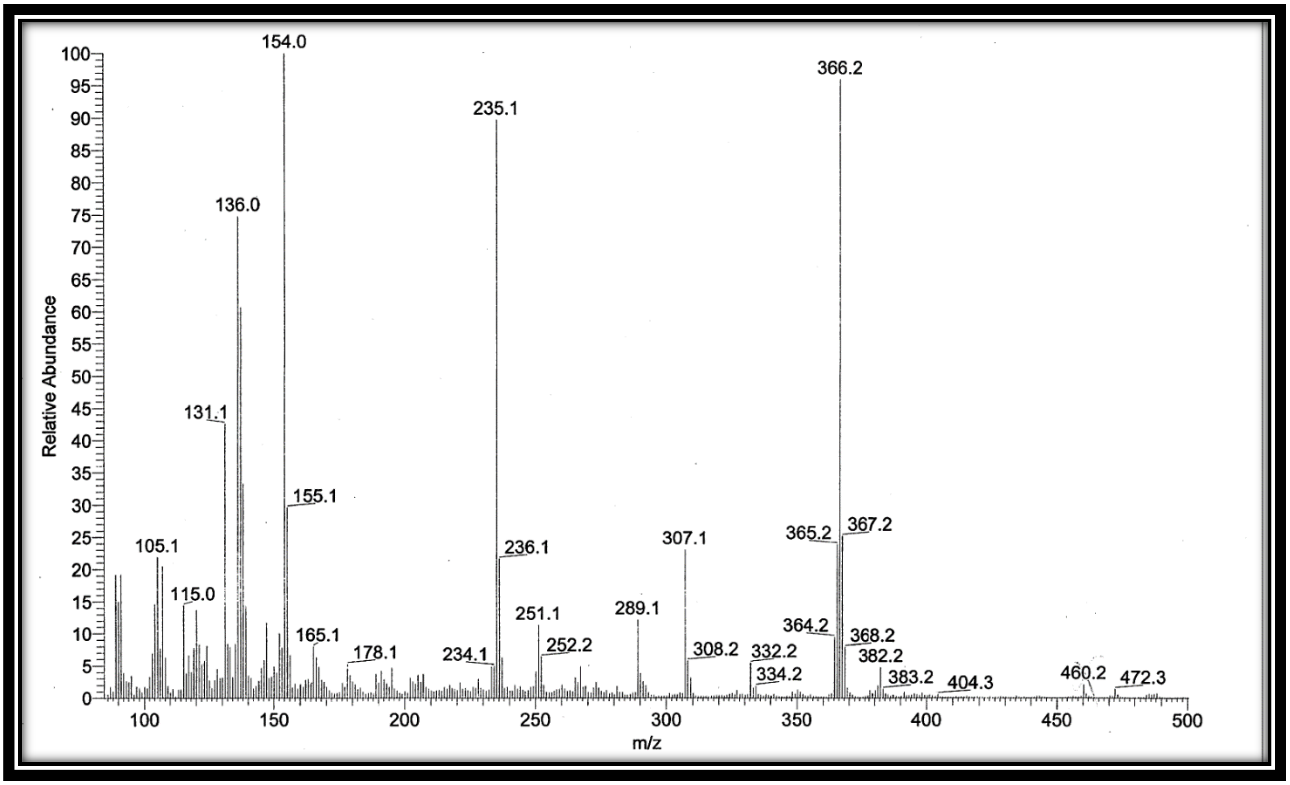


**Figure 16.** Mass spectrum of compound **2d**

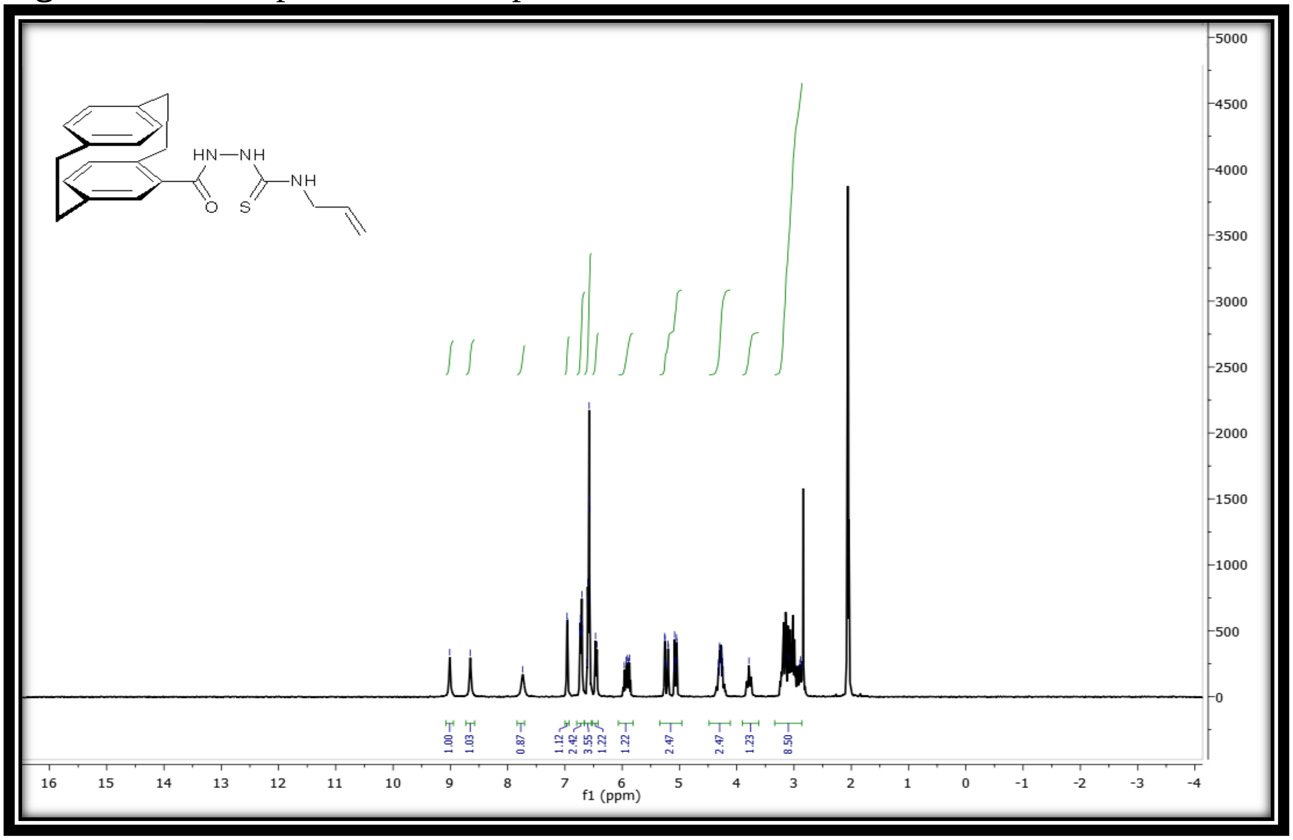


**Figure 17.** $^{1}$H NMR spectrum of compound **2d**

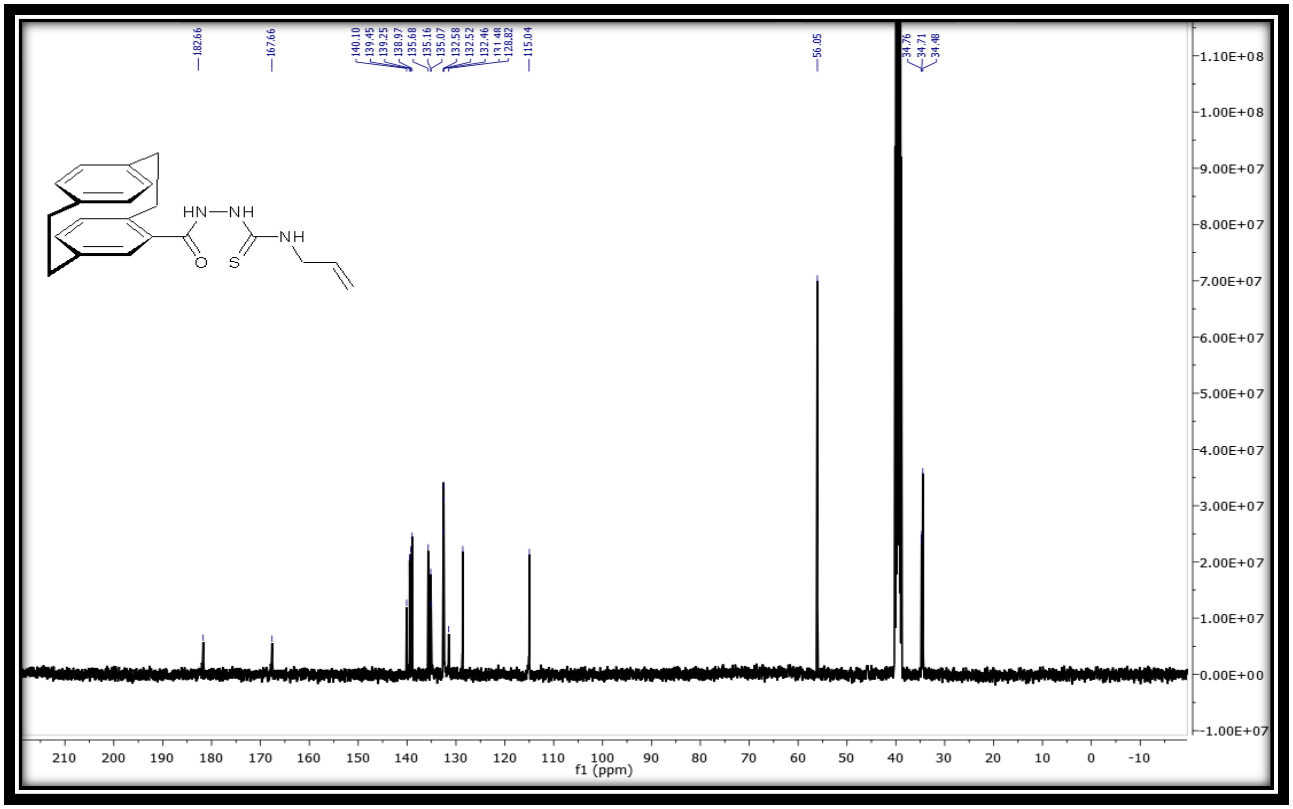


**Figure 18.** $^{13}$**C** NMR spectrum of compound **2d**

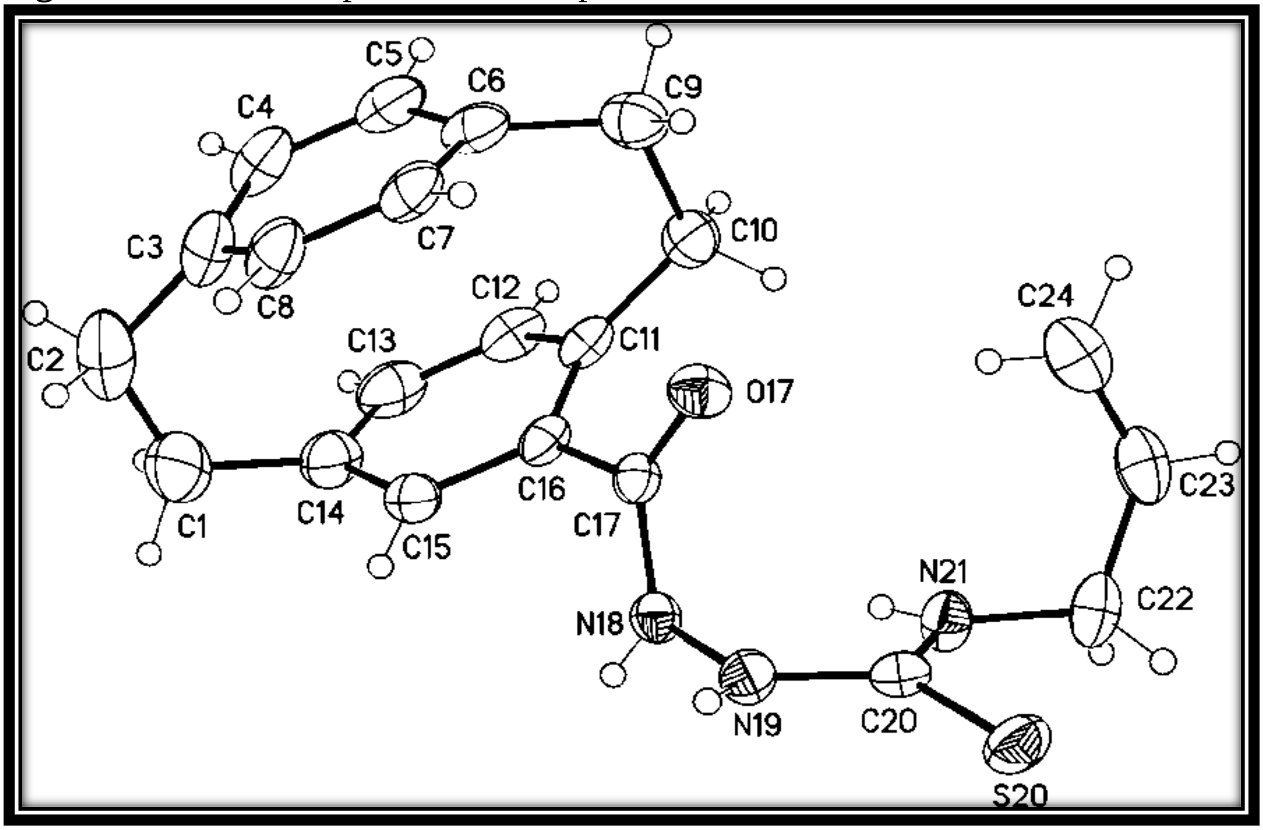


**Figure 19.** Molecular structure of compound **2d**

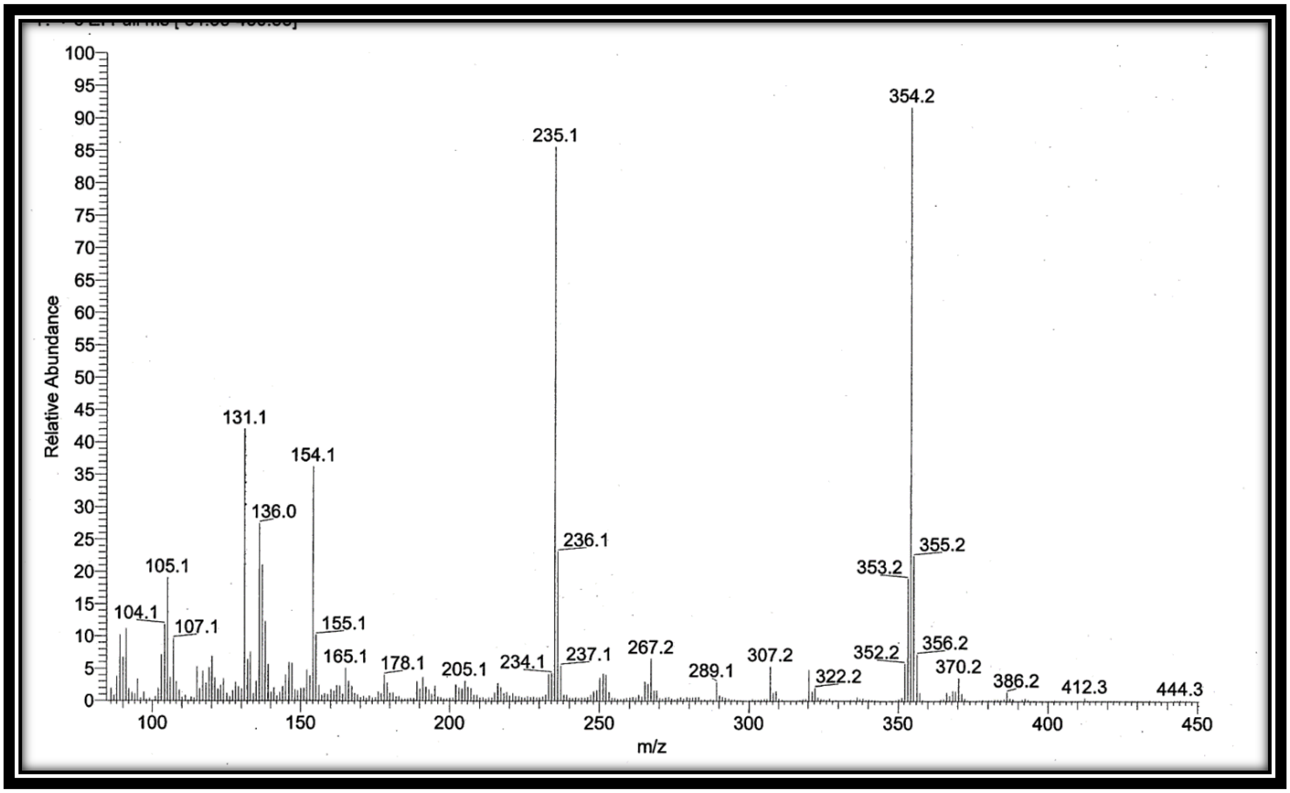


**Figure 20.** Mass spectrum of compound **2e**

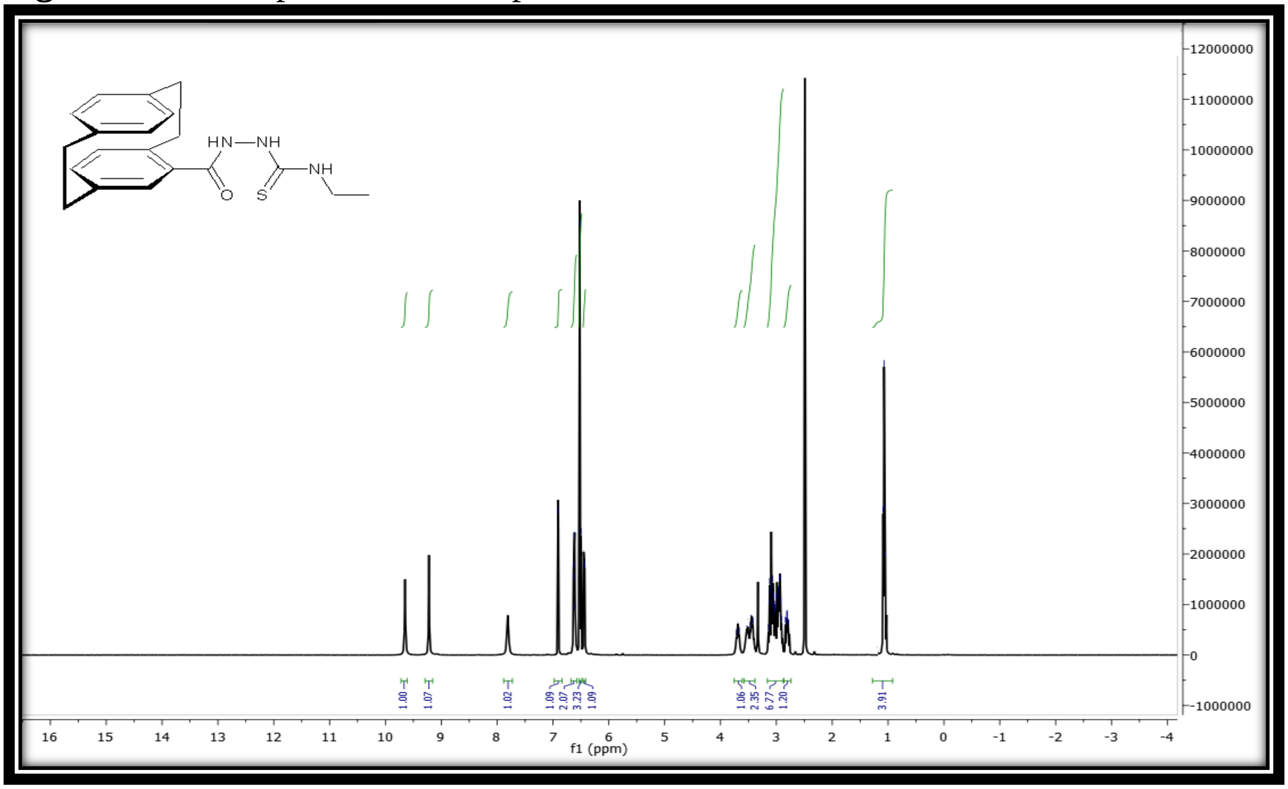


**Figure 21.** $^{1}$H NMR spectrum of compound **2e**

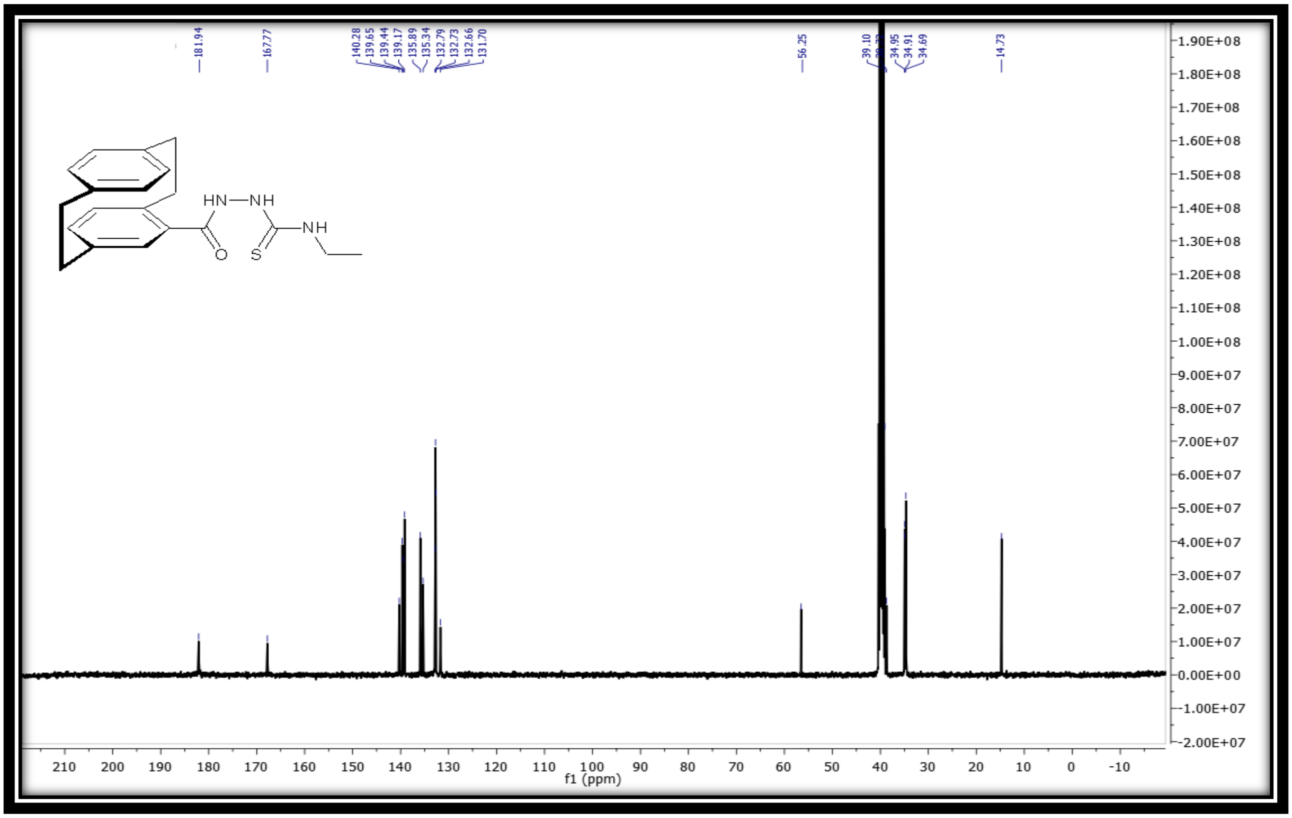


**Figure 22.** $^{13}$C NMR spectrum of compound **2e**

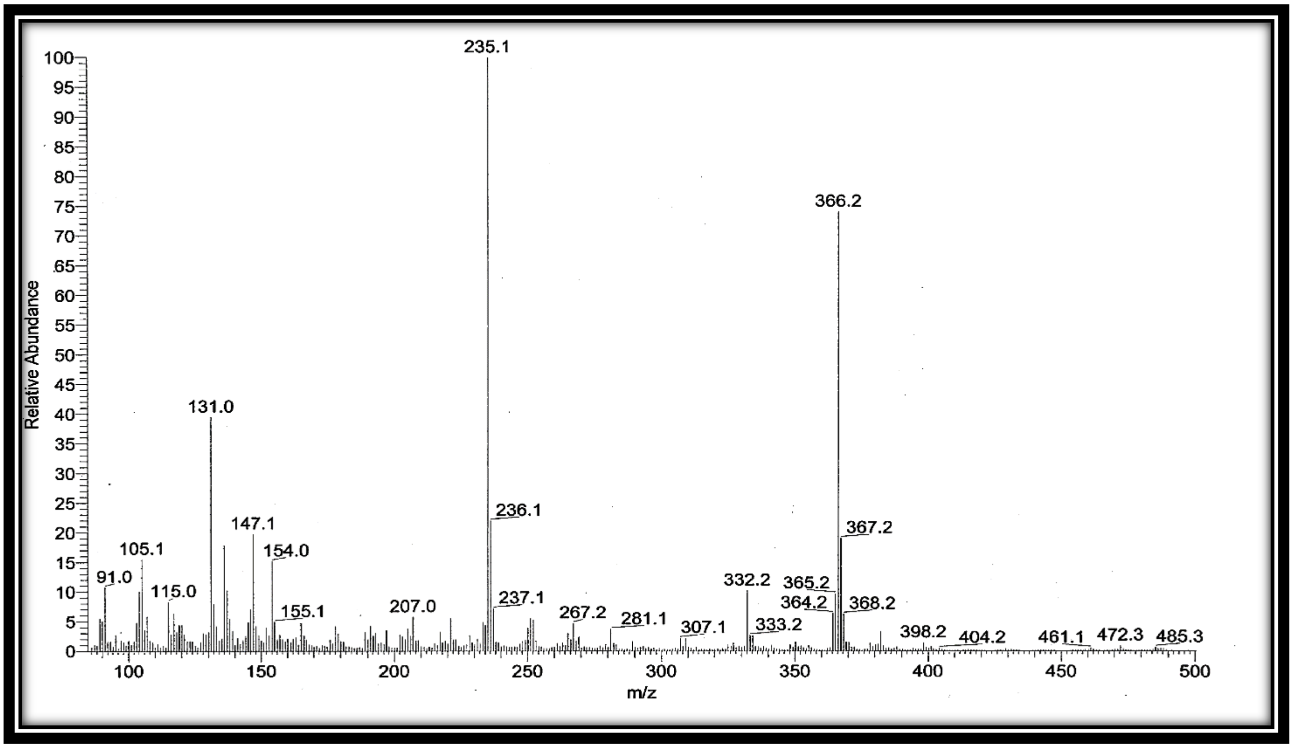


**Figure 23.** Mass spectrum of compound **2f**

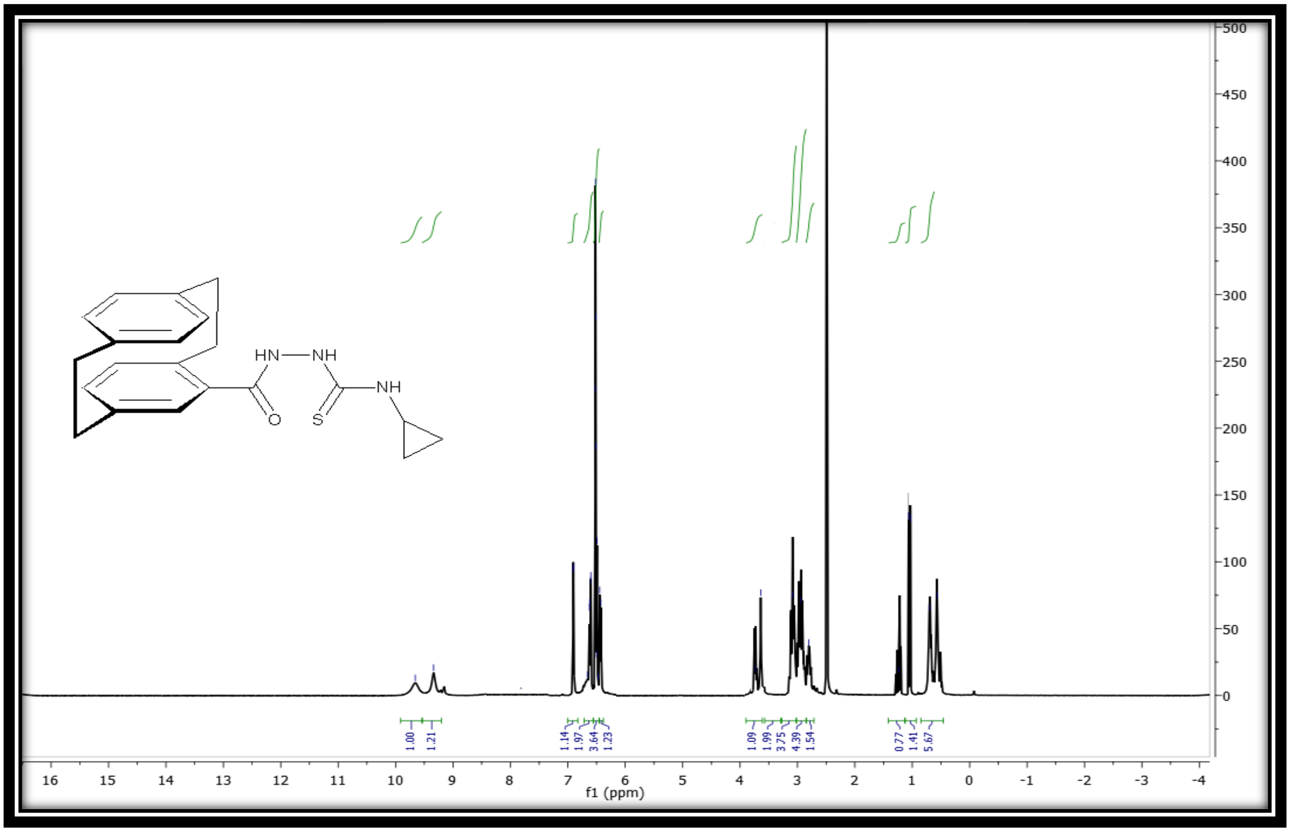


**Figure 24.** $^{1}$H NMR spectrum of compound **2f**

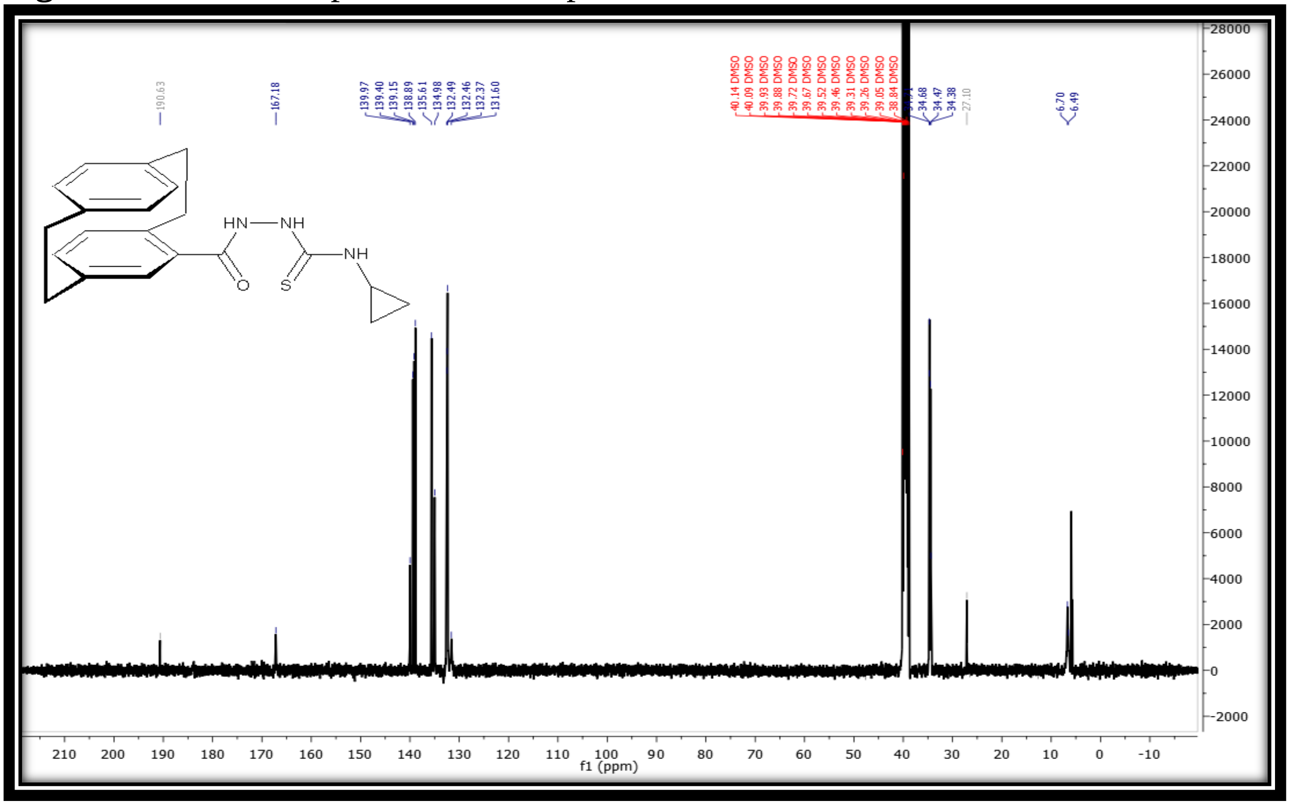


**Figure 25.** $^{13}$C NMR spectrum of compound **2f**

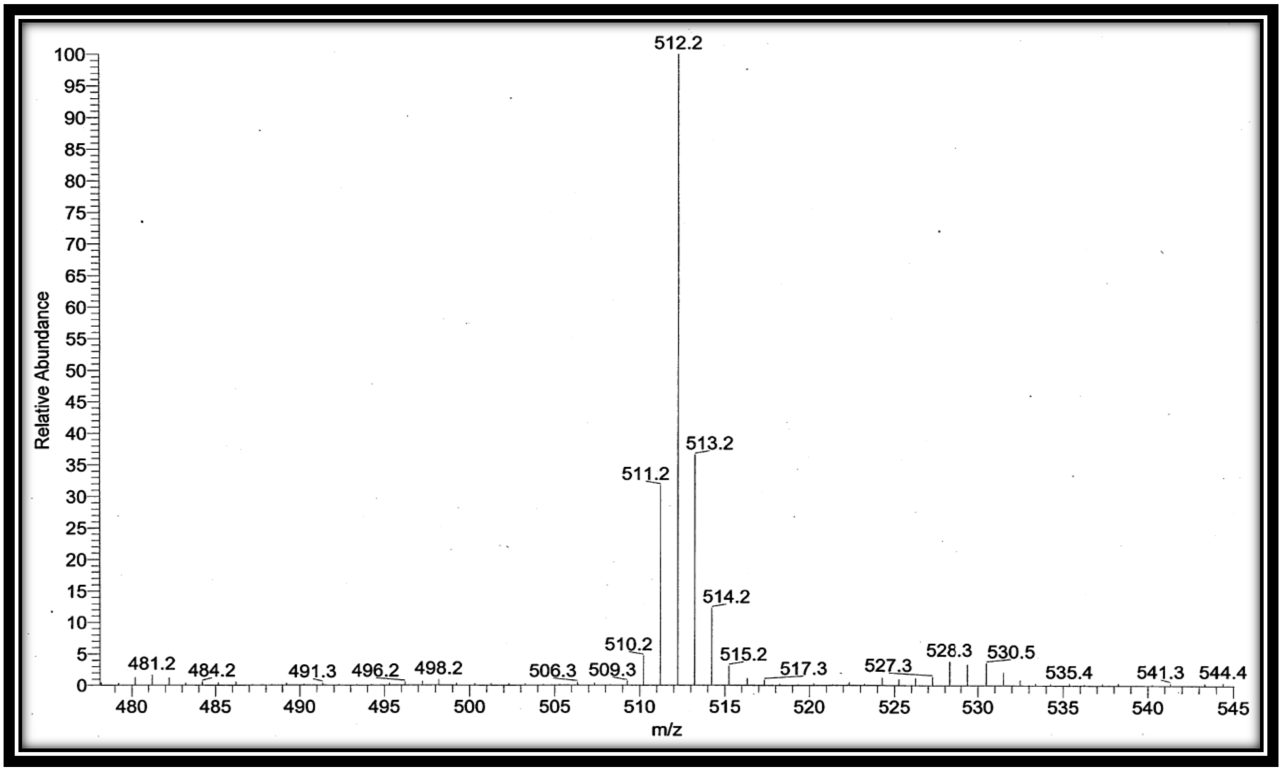


**Figure 26.** Mass spectrum of compound **3a**

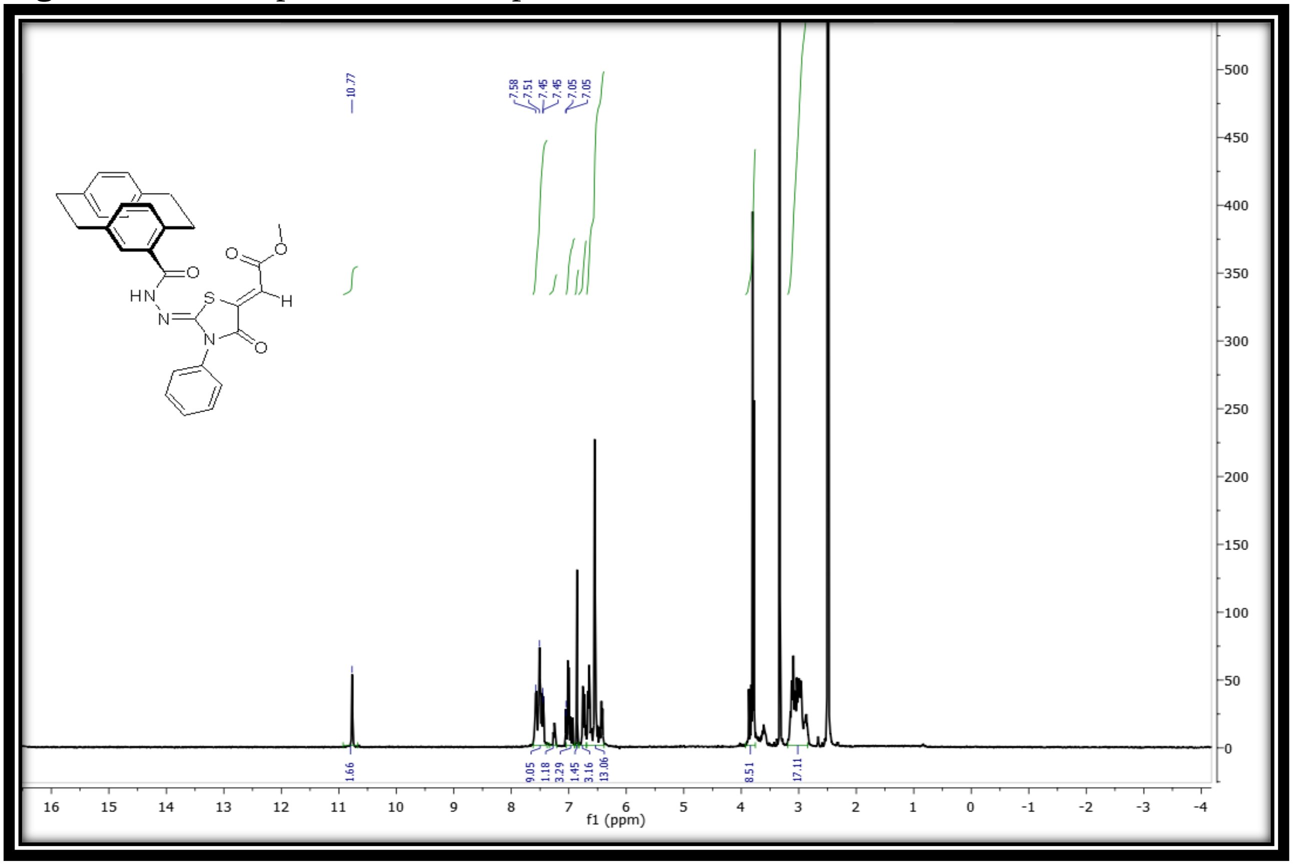


**Figure 27.** $^{1}$H NMR spectrum of compound **3a**

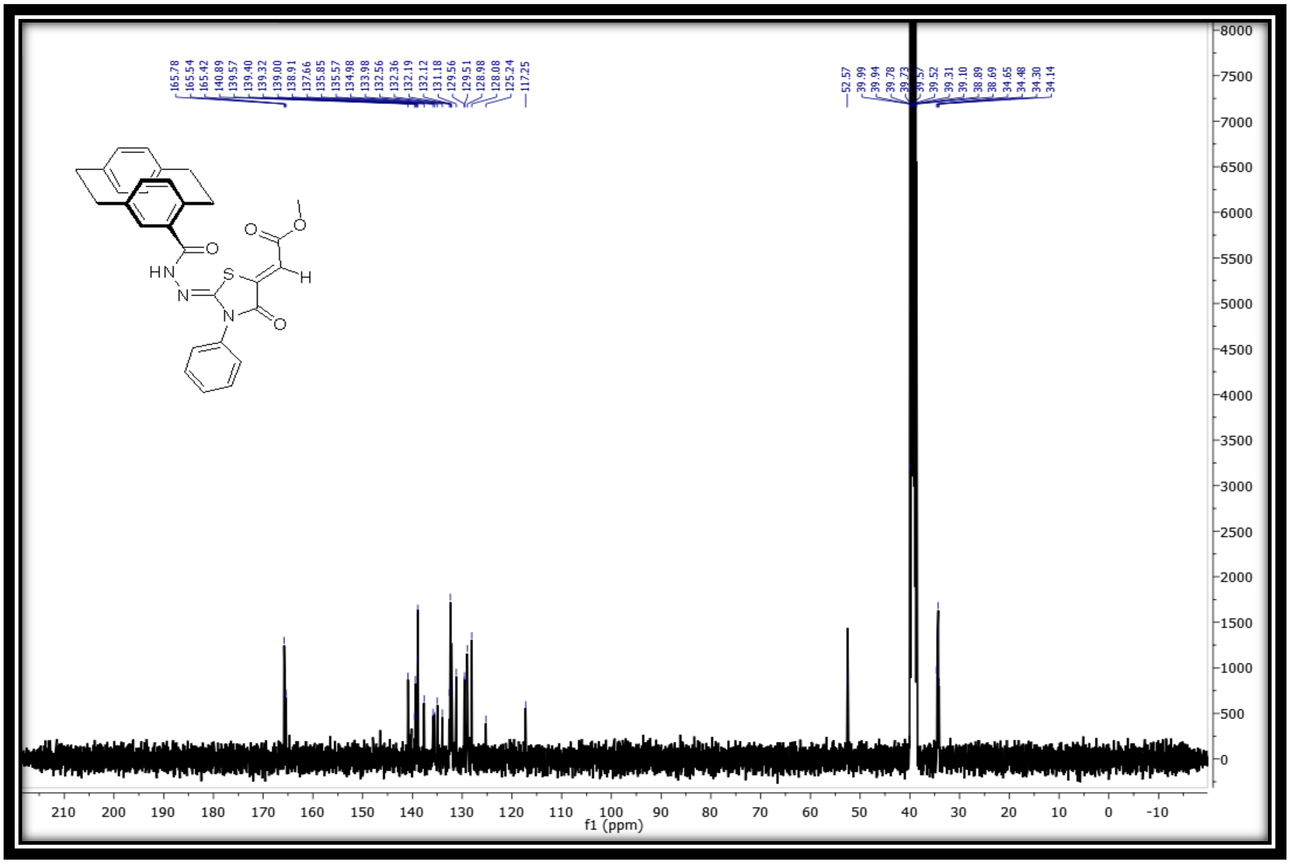

**Figure 28.** $^{13}$**C** NMR spectrum of compound **3a**

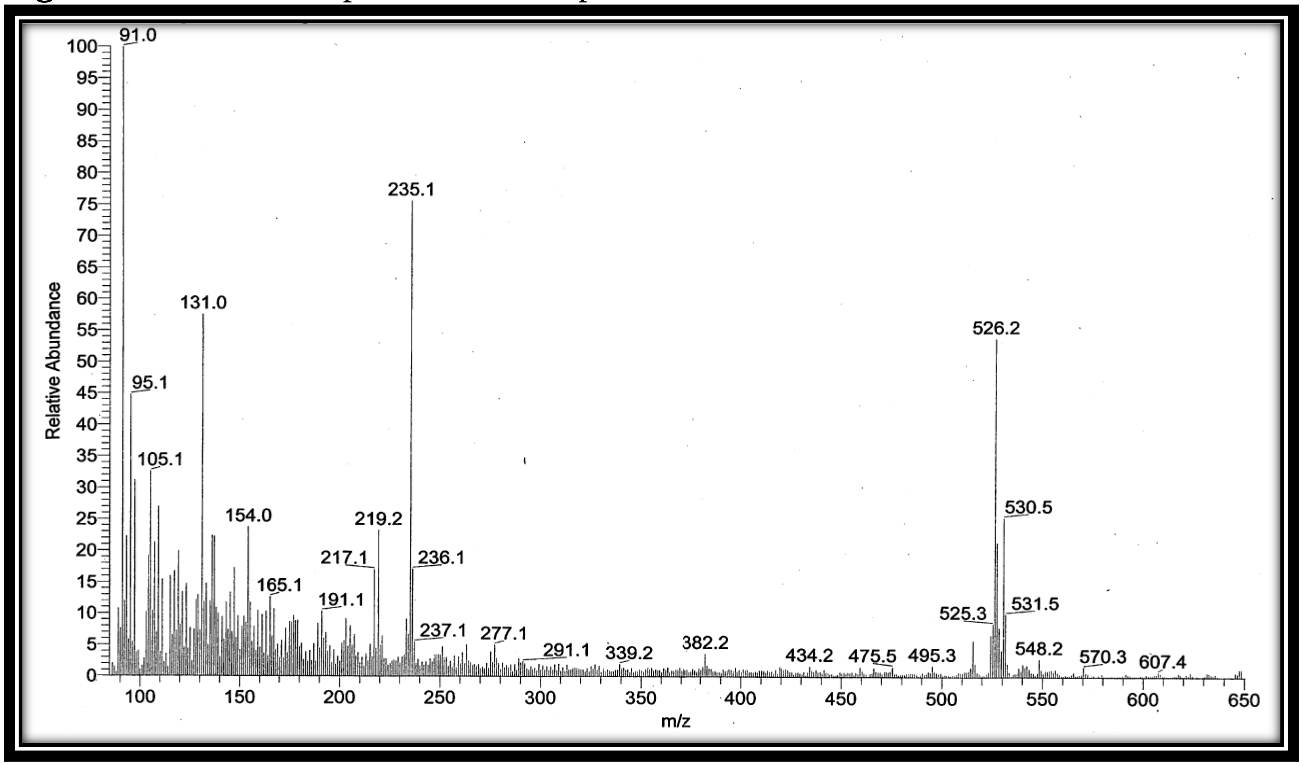

**Figure 29.** Mass spectrum of compound **3b**

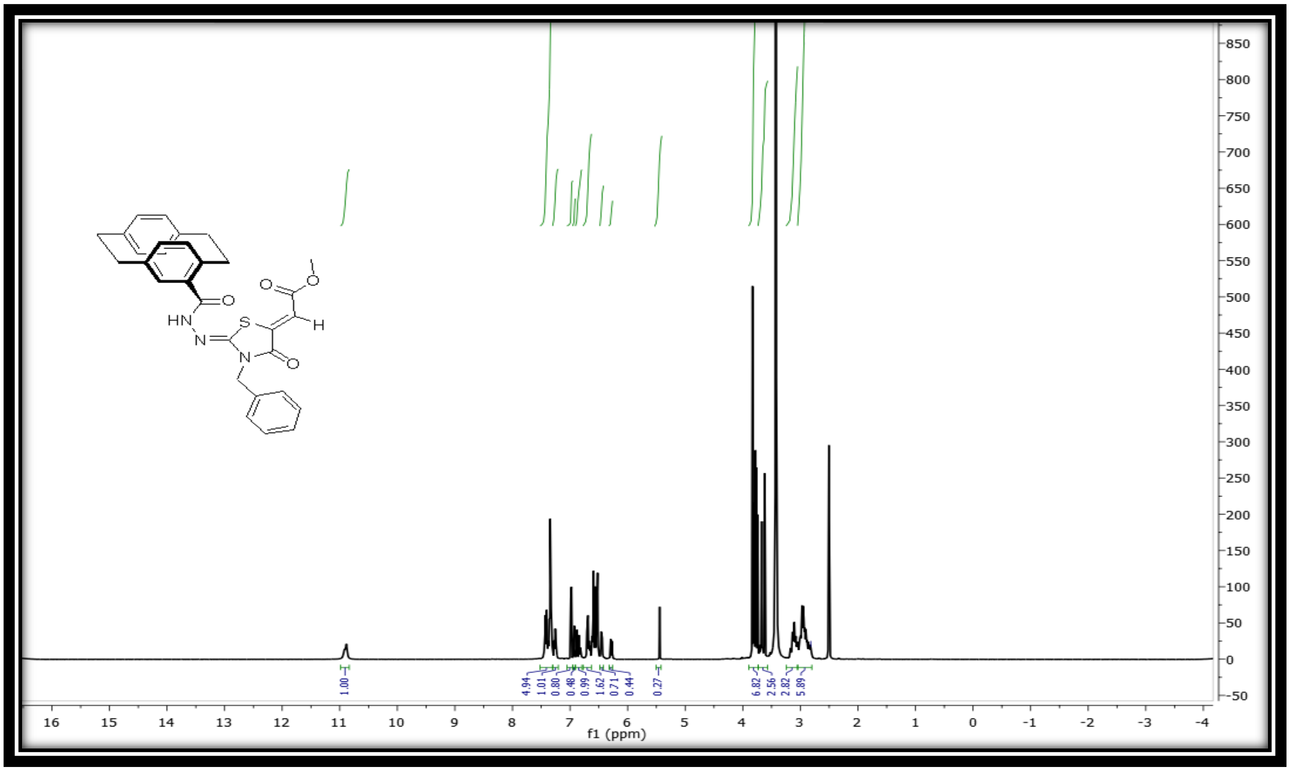


**Figure 30.** $^{1}$H NMR spectrum of compound **3b**

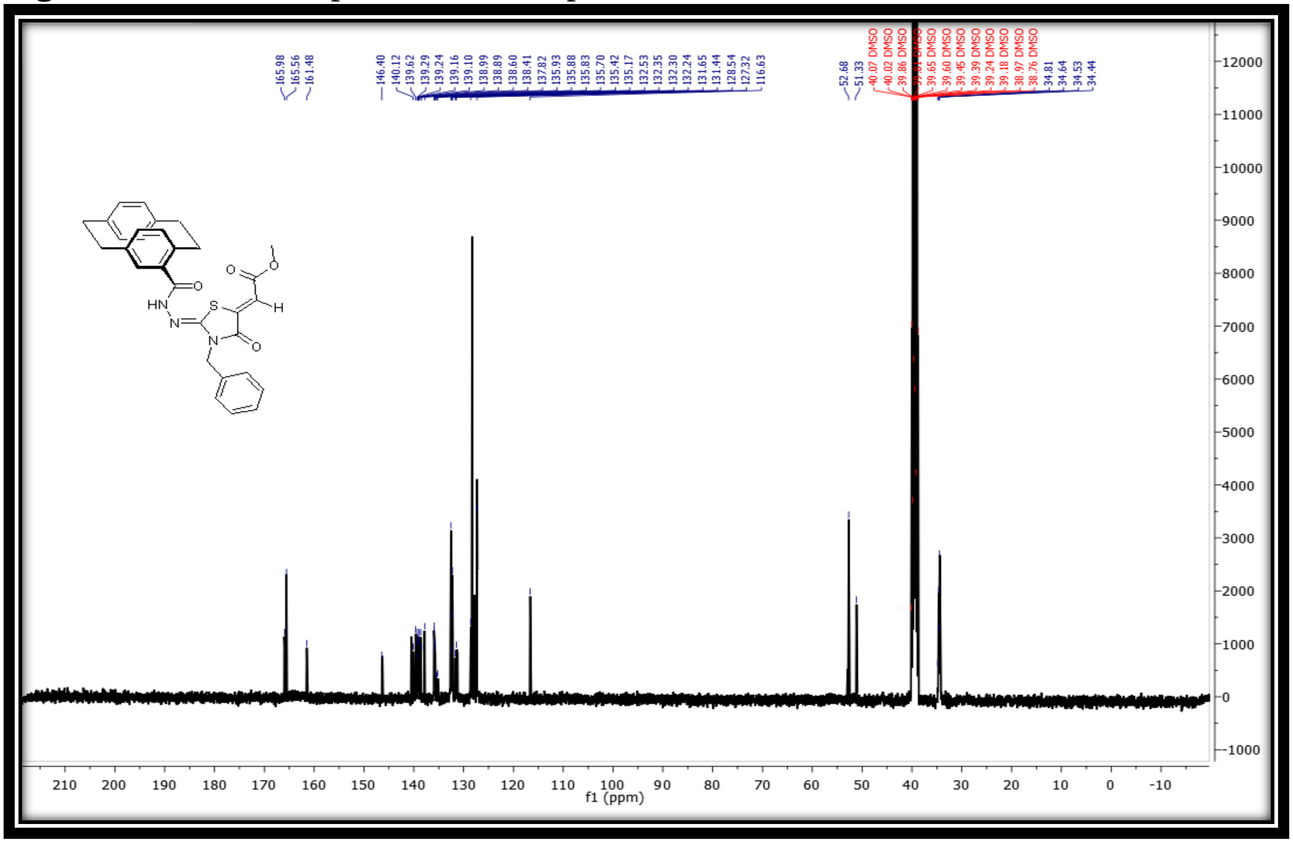


**Figure 32.** $^{13}$**C** NMR spectrum of compound **3b**

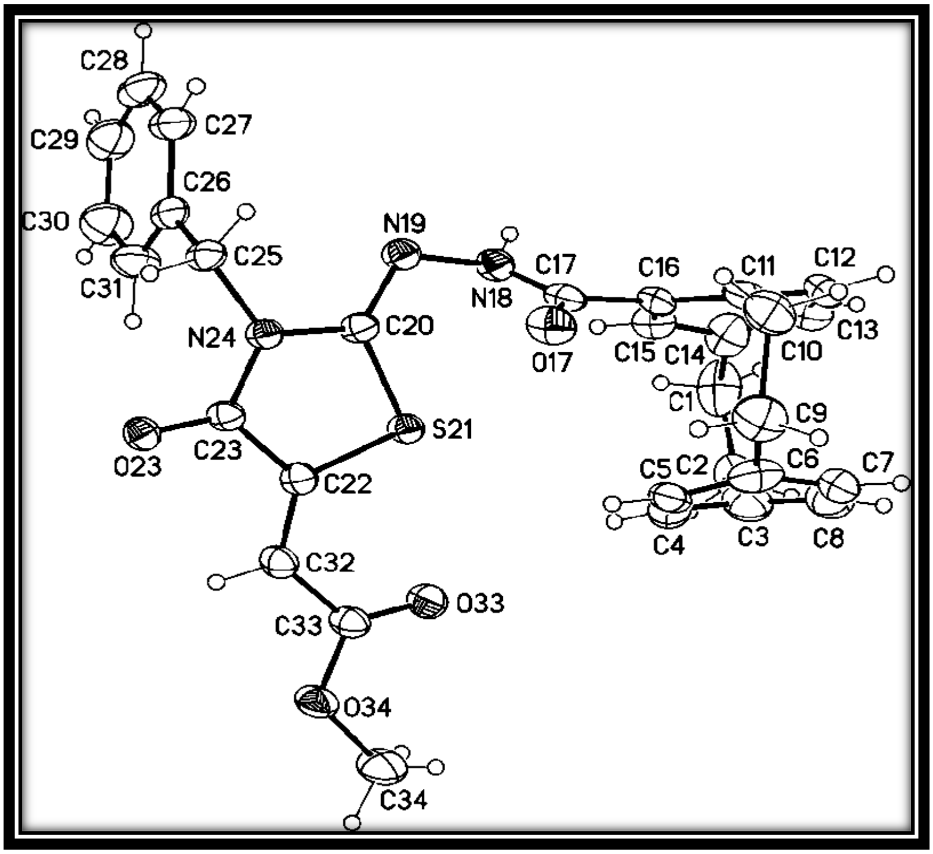


**Figure 33.** Molecular structure of compound **3b**

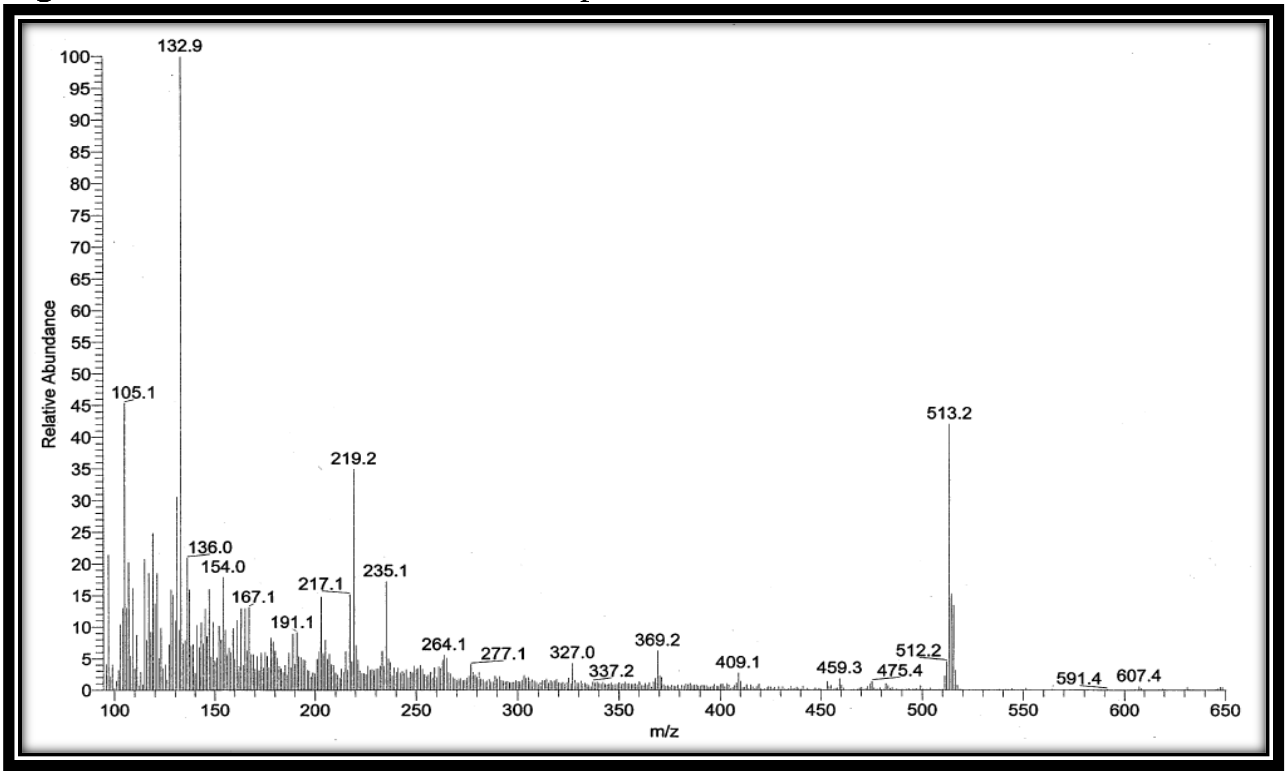


**Figure 34.** Mass spectrum of compound **3c**

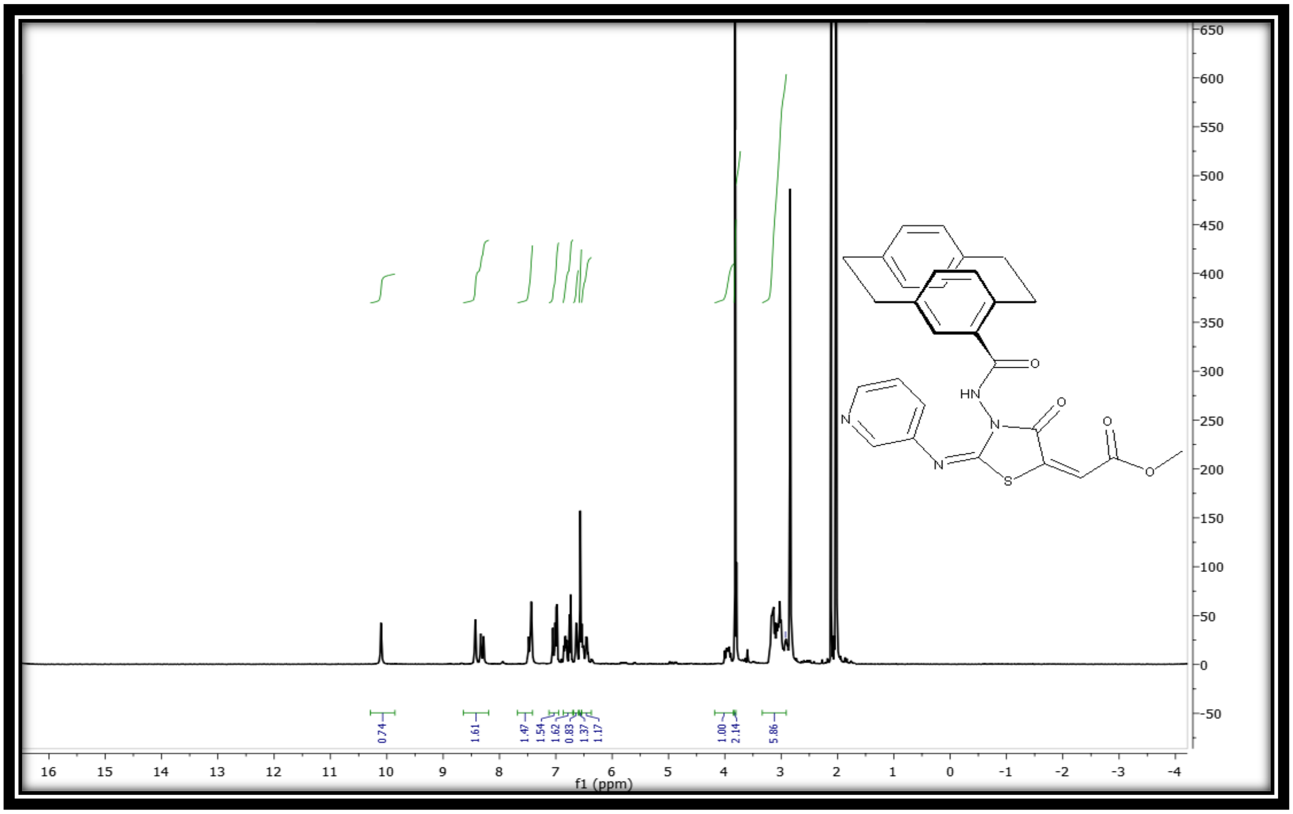


**Figure 35.** $^{1}$H NMR spectrum of compound **3c**

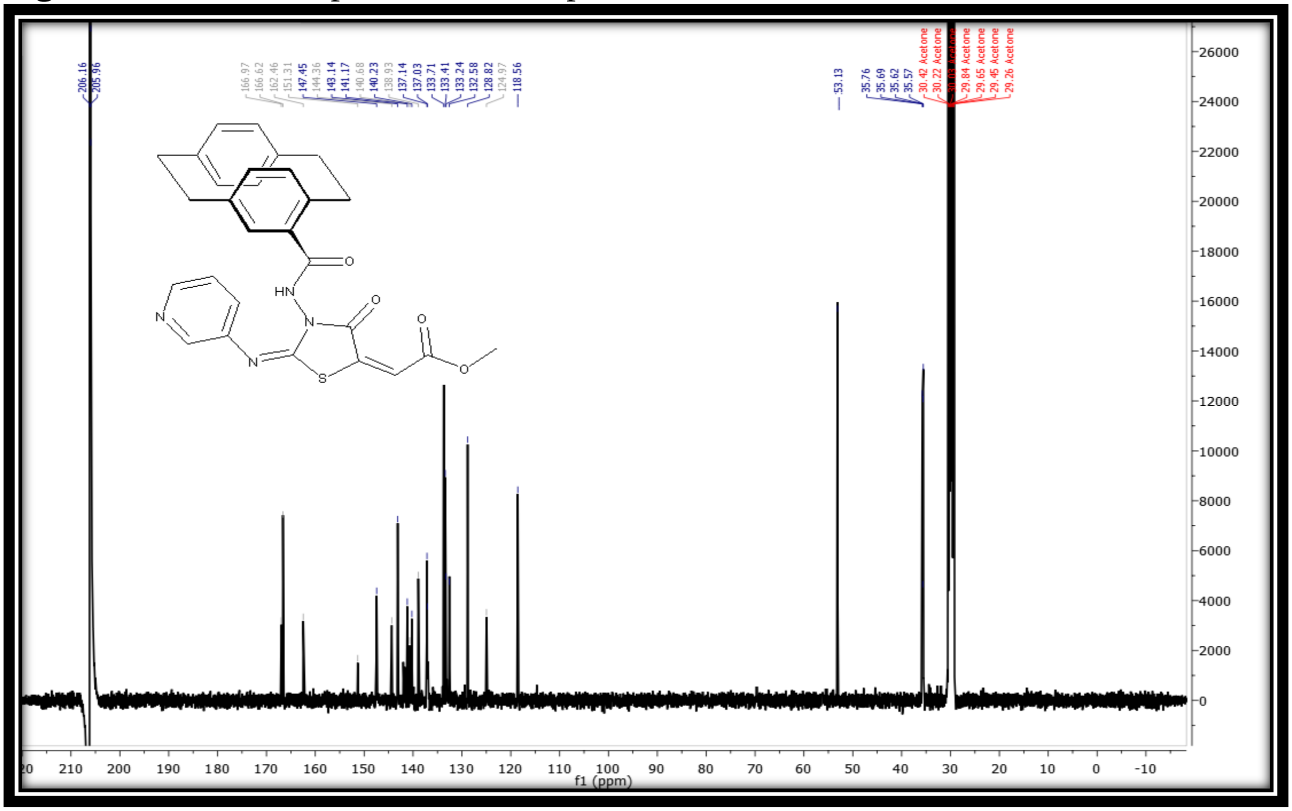


**Figure 36.** $^{13}$C NMR spectrum of compound **3c**

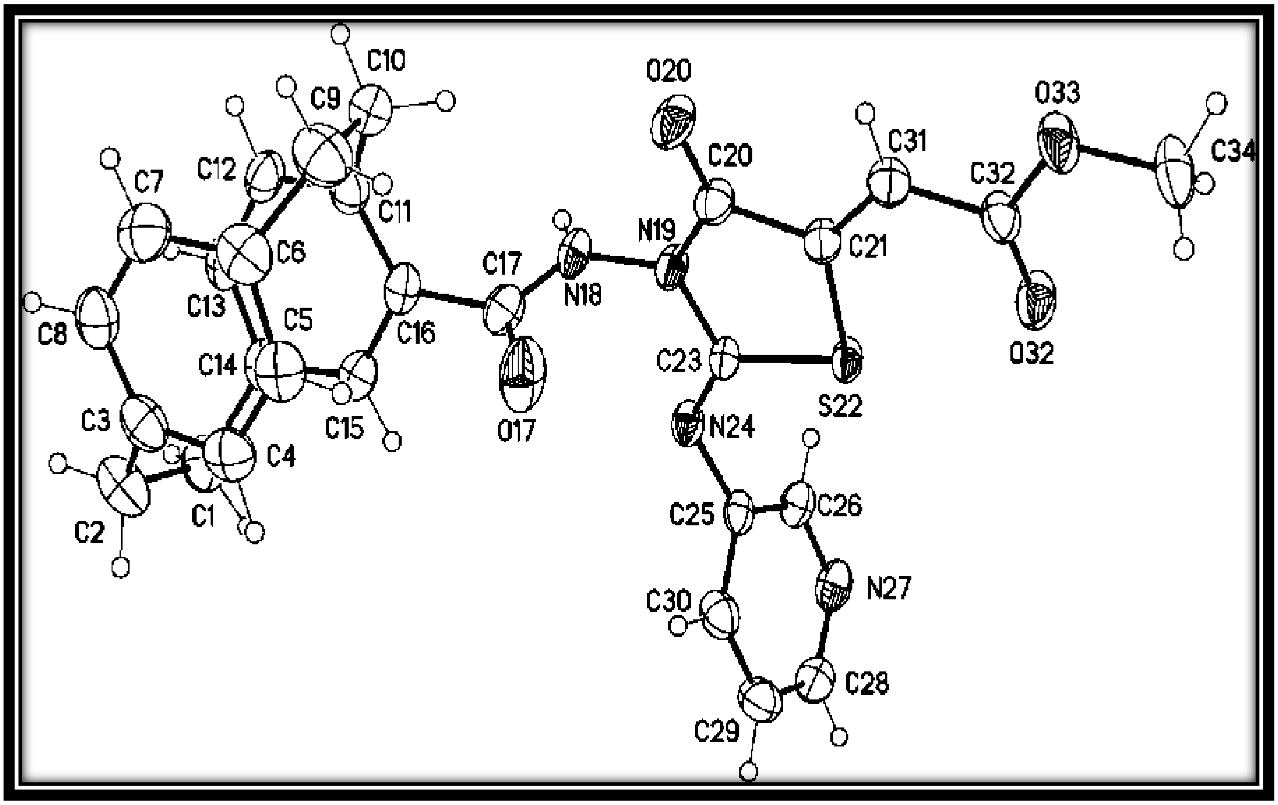


**Figure 37.** Molecular structure of compound **3c**

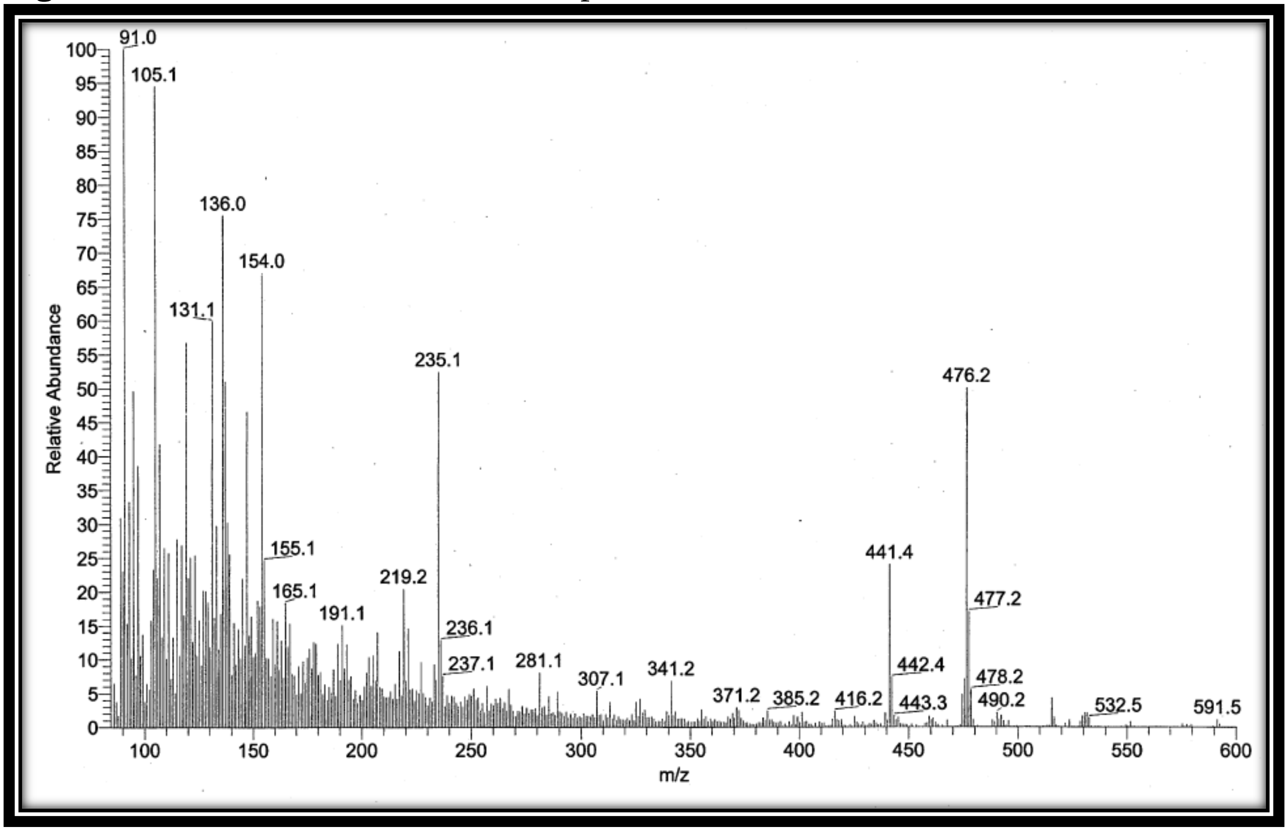


**Figure 38.** Mass spectrum of compound **3d**

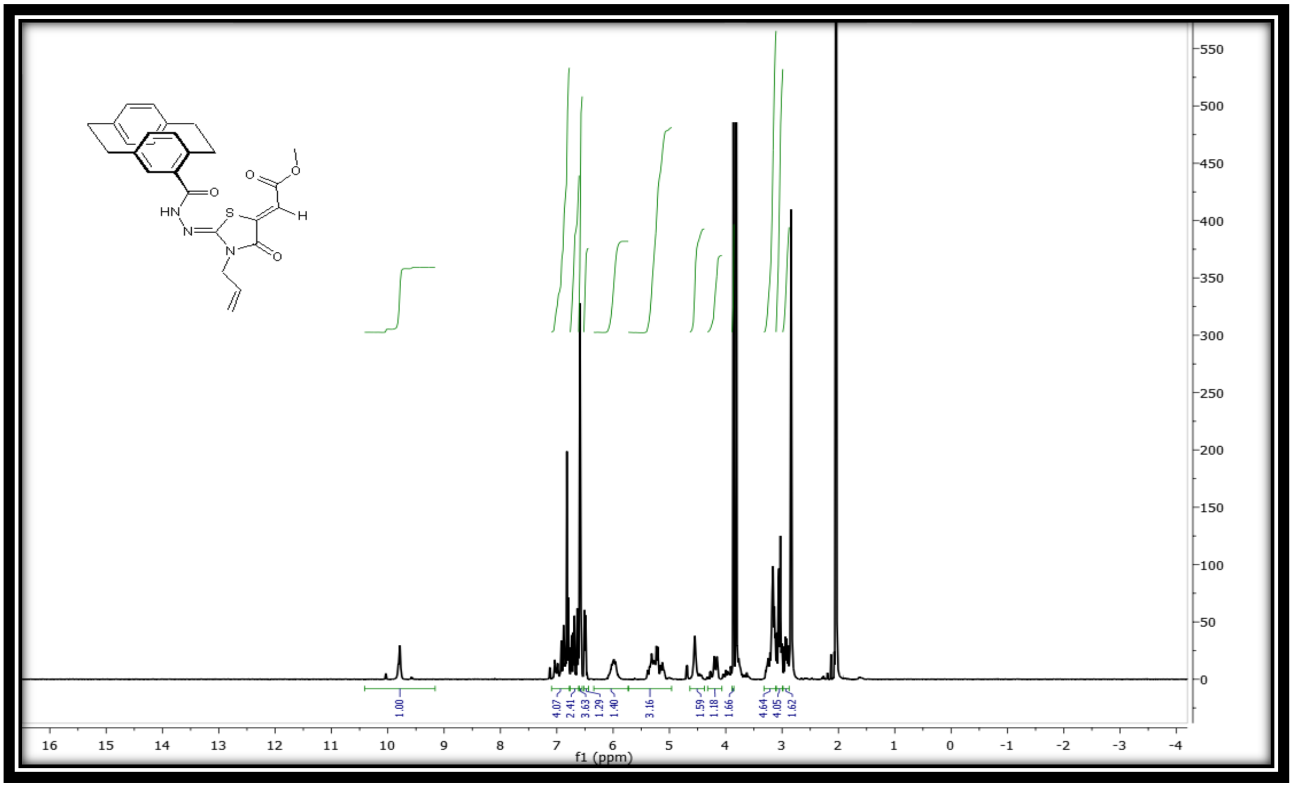

**Figure 39.** $^{1}$H NMR spectrum of compound **3d**

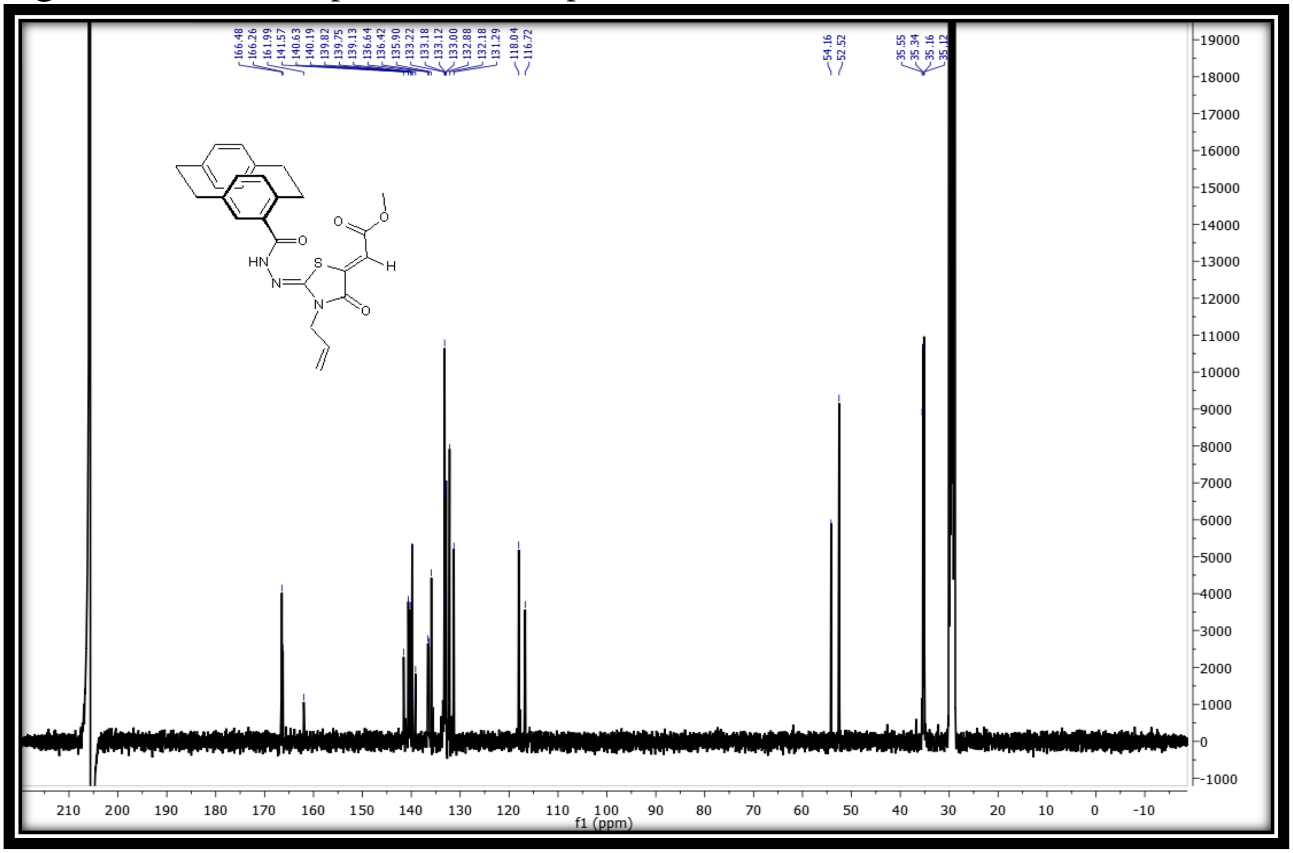

**Figure 40.** $^{13}$C NMR spectrum of compound **3d**

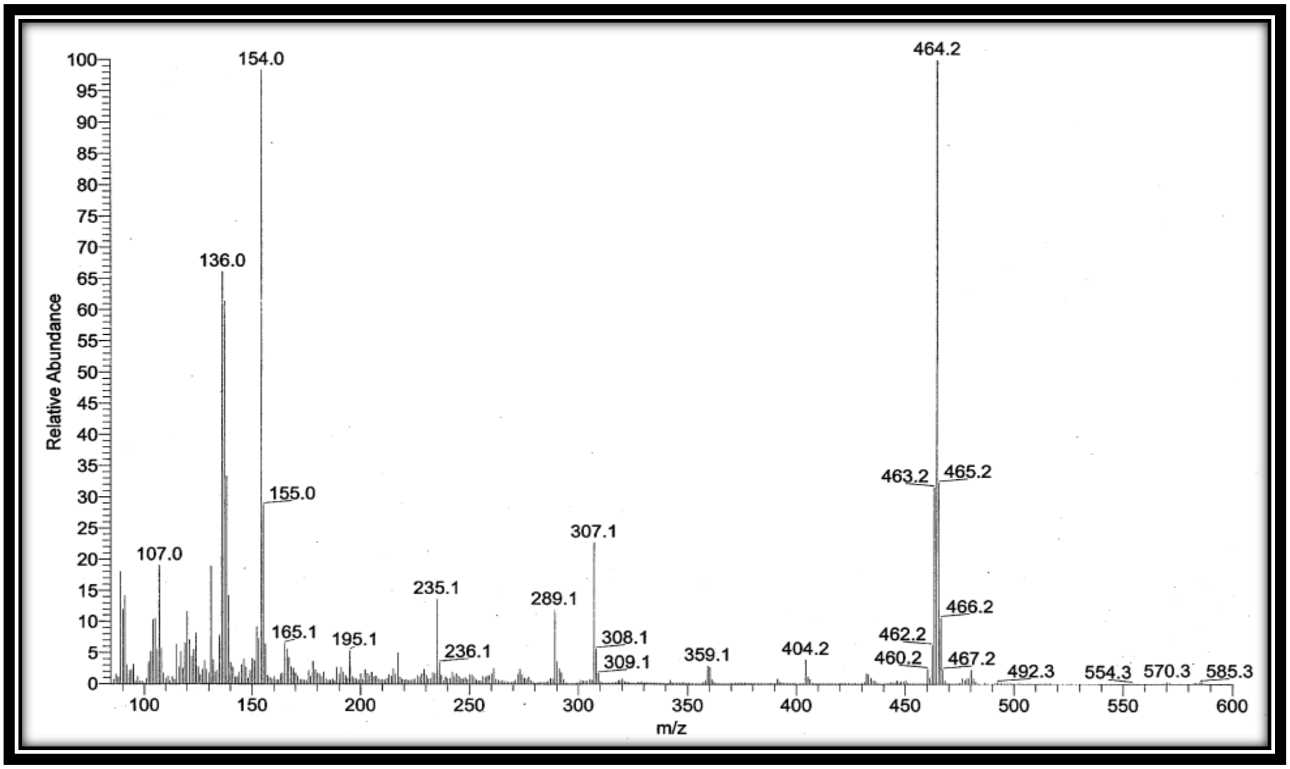


Figure 41. Mass spectrum of compound **3e**

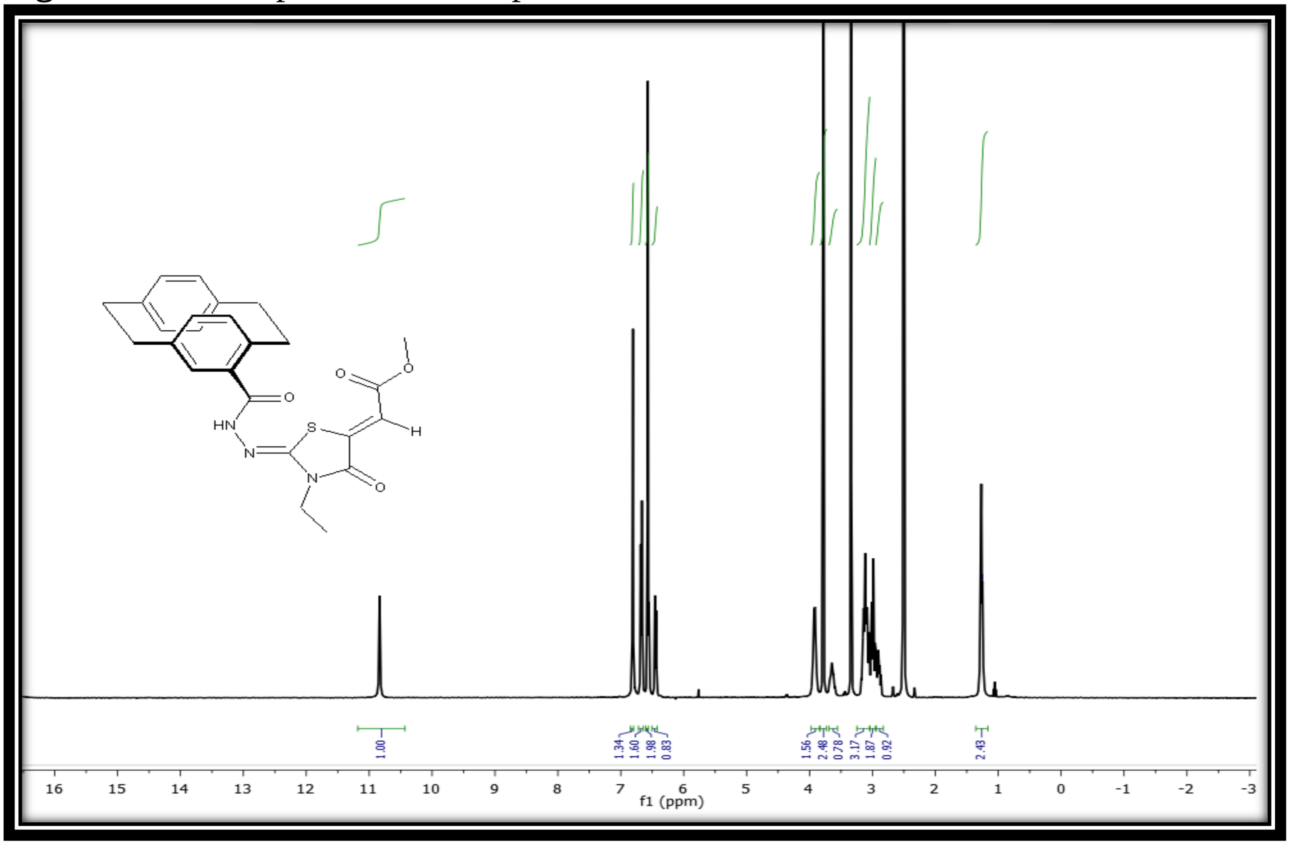


Figure 42. $^{1}$H NMR spectrum of compound **3e**

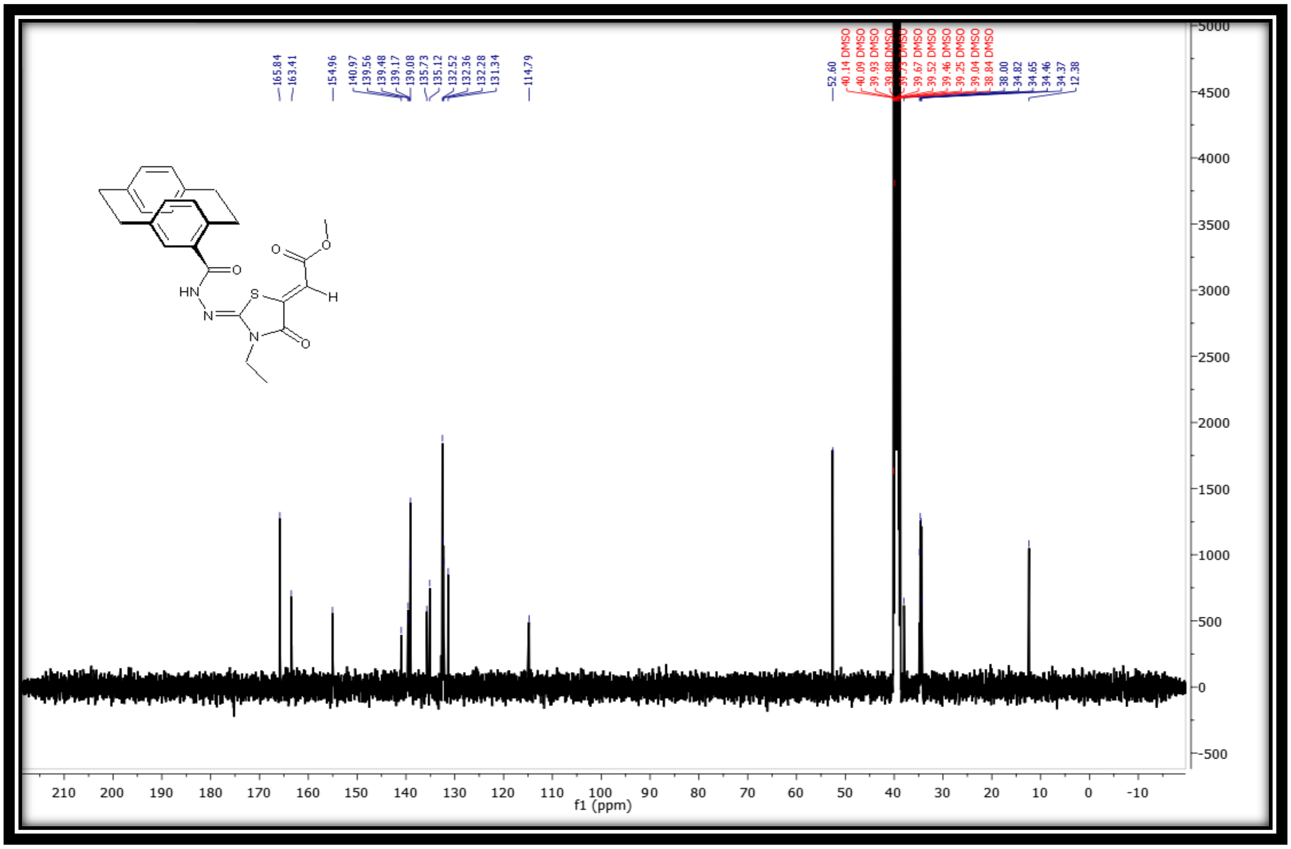

**Figure 43.** $^{13}$**C** NMR spectrum of compound **3e**

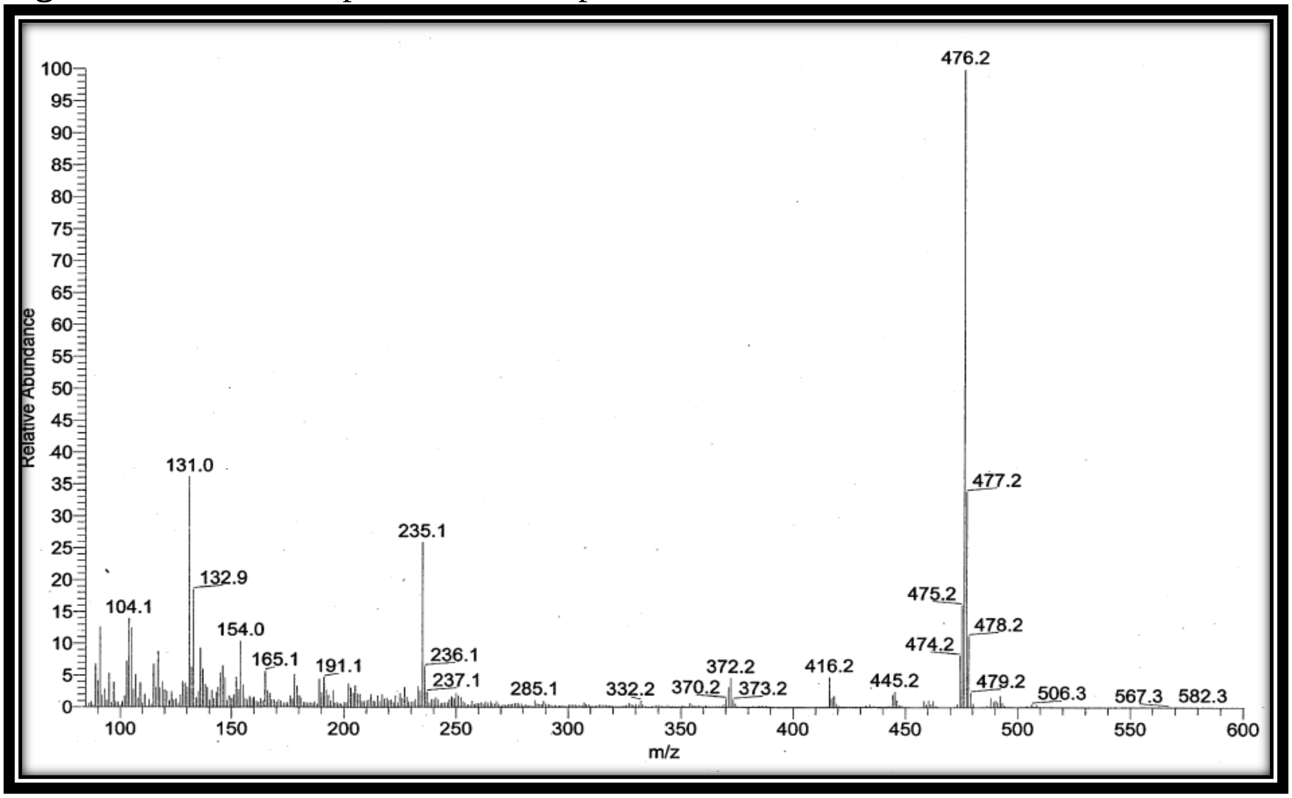

**Figure 44.** Mass spectrum of compound **3f**

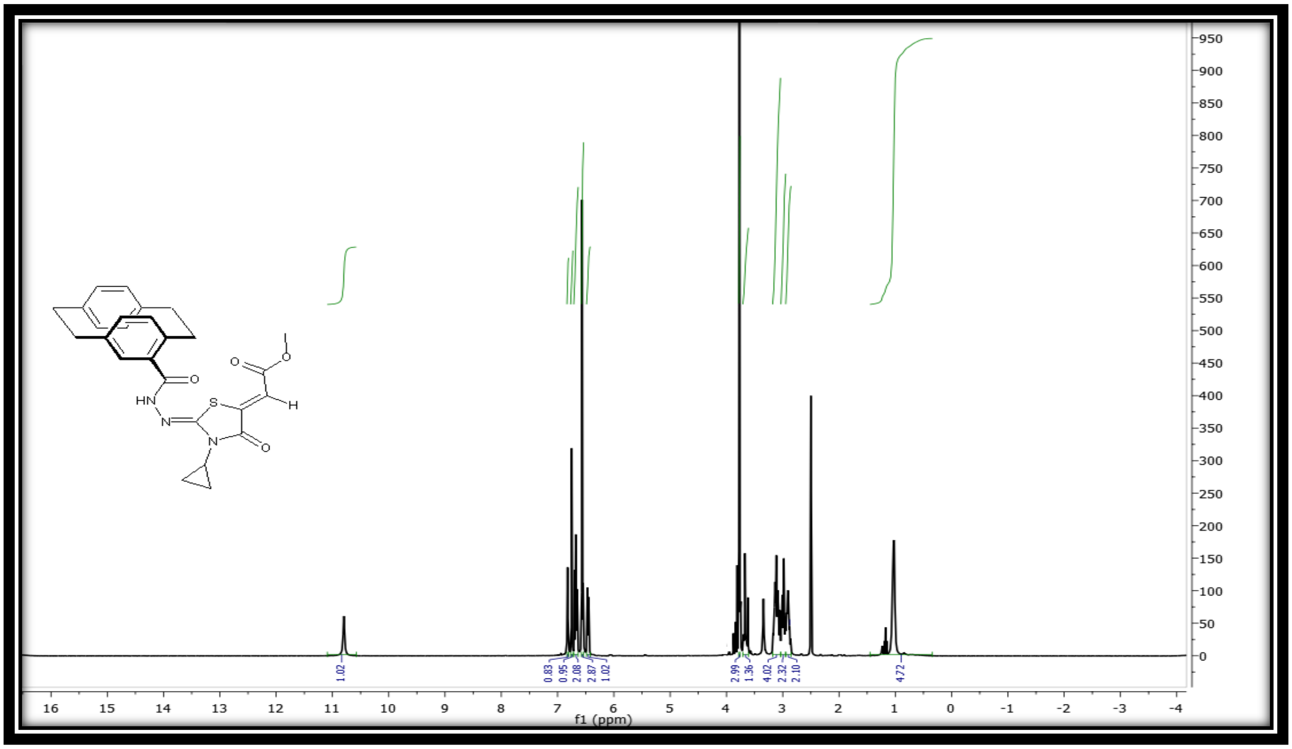

**Figure 45.** $^{1}$H NMR spectrum of compound **3f**

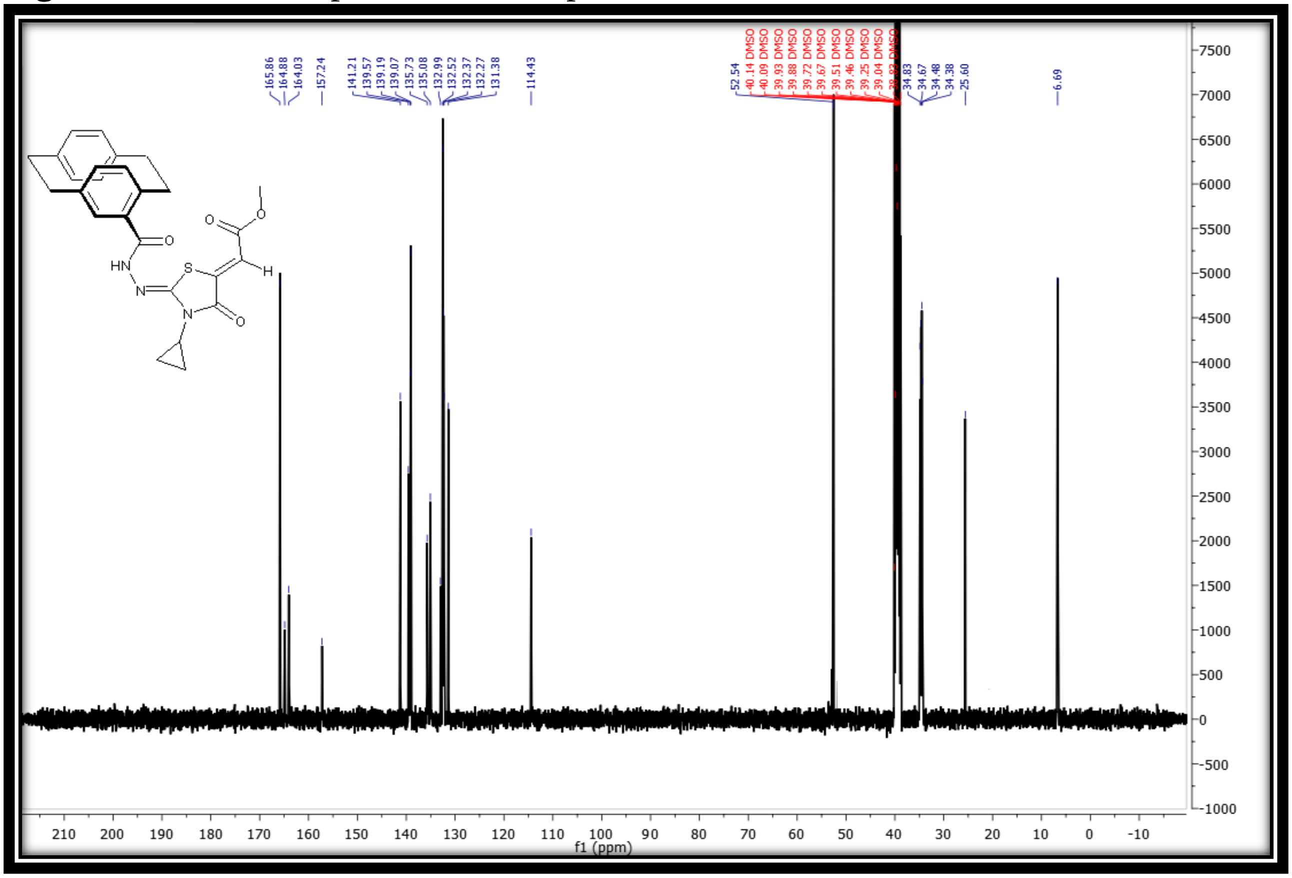

**Figure 46.** $^{13}$**C** NMR spectrum of compound **3f**

## Biology:

# One dose antriproliferative assay at the National Cancer Institute (NCI), USA.

## Compound 3a

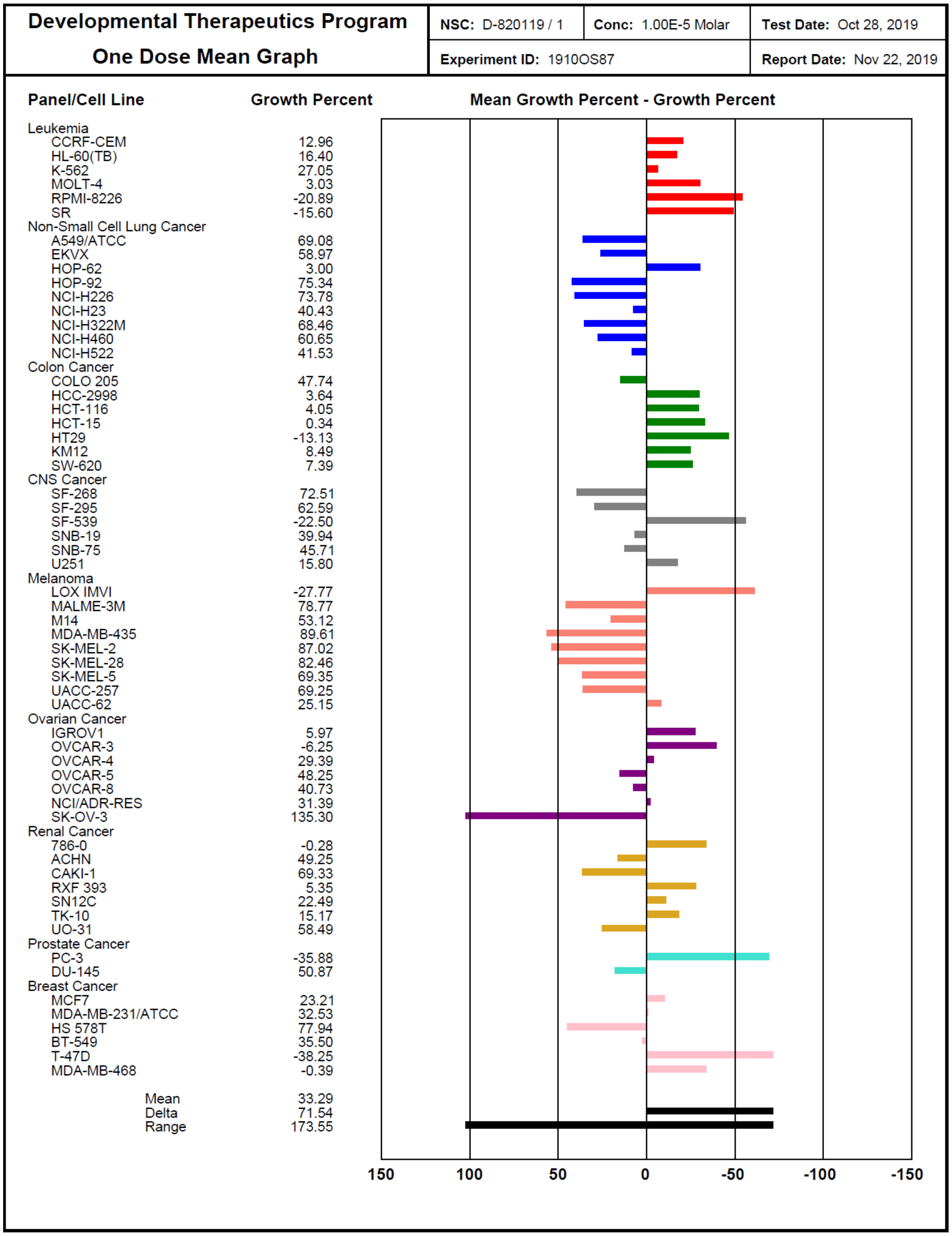

# Compound 3b

| Developmental Therapeutics Program One Dose Mean Graph | NSC: D-820123 / 1 | Conc: 1.00E-5 Molar | Test Date: Oct 28, 2019 |
|---|---|---|---|
| | Experiment ID: 1910OS87 | | Report Date: Nov 22, 2019 |

| Panel/Cell Line | Growth Percent |
|---|---|
| Leukemia | |
| CCRF-CEM | 68.73 |
| HL-60(TB) | 51.36 |
| K-562 | 79.28 |
| MOLT-4 | 78.14 |
| RPMI-8226 | 16.99 |
| SR | 25.82 |
| Non-Small Cell Lung Cancer | |
| A549/ATCC | 97.96 |
| EKVX | 97.39 |
| HOP-62 | 95.63 |
| HOP-92 | 91.86 |
| NCI-H226 | 92.52 |
| NCI-H23 | 90.82 |
| NCI-H322M | 98.28 |
| NCI-H460 | 99.25 |
| NCI-H522 | 82.26 |
| Colon Cancer | |
| COLO 205 | 104.13 |
| HCC-2998 | 108.95 |
| HCT-116 | 93.65 |
| HCT-15 | 88.25 |
| HT29 | 92.29 |
| KM12 | 92.68 |
| SW-620 | 89.16 |
| CNS Cancer | |
| SF-268 | 96.92 |
| SF-295 | 94.37 |
| SF-539 | 88.37 |
| SNB-19 | 83.36 |
| SNB-75 | 99.75 |
| U251 | 85.34 |
| Melanoma | |
| LOX IMVI | 90.22 |
| MALME-3M | 102.17 |
| M14 | 92.01 |
| MDA-MB-435 | 100.69 |
| SK-MEL-2 | 102.54 |
| SK-MEL-28 | 99.14 |
| SK-MEL-5 | 96.36 |
| UACC-257 | 99.17 |
| UACC-62 | 89.33 |
| Ovarian Cancer | |
| IGROV1 | 84.56 |
| OVCAR-3 | 95.49 |
| OVCAR-4 | 92.24 |
| OVCAR-5 | 99.39 |
| OVCAR-8 | 98.49 |
| NCI/ADR-RES | 95.30 |
| SK-OV-3 | 118.82 |
| Renal Cancer | |
| 786-0 | 91.48 |
| ACHN | 96.41 |
| CAKI-1 | 88.98 |
| RXF 393 | 95.22 |
| SN12C | 91.13 |
| TK-10 | 118.19 |
| UO-31 | 78.14 |
| Prostate Cancer | |
| PC-3 | 81.81 |
| DU-145 | 102.20 |
| Breast Cancer | |
| MCF7 | 73.25 |
| MDA-MB-231/ATCC | 89.36 |
| HS 578T | 100.38 |
| BT-549 | 81.59 |
| T-47D | 68.87 |
| MDA-MB-468 | 81.97 |
| Mean | 89.46 |
| Delta | 72.47 |
| Range | 101.83 |

Mean Growth Percent - Growth Percent

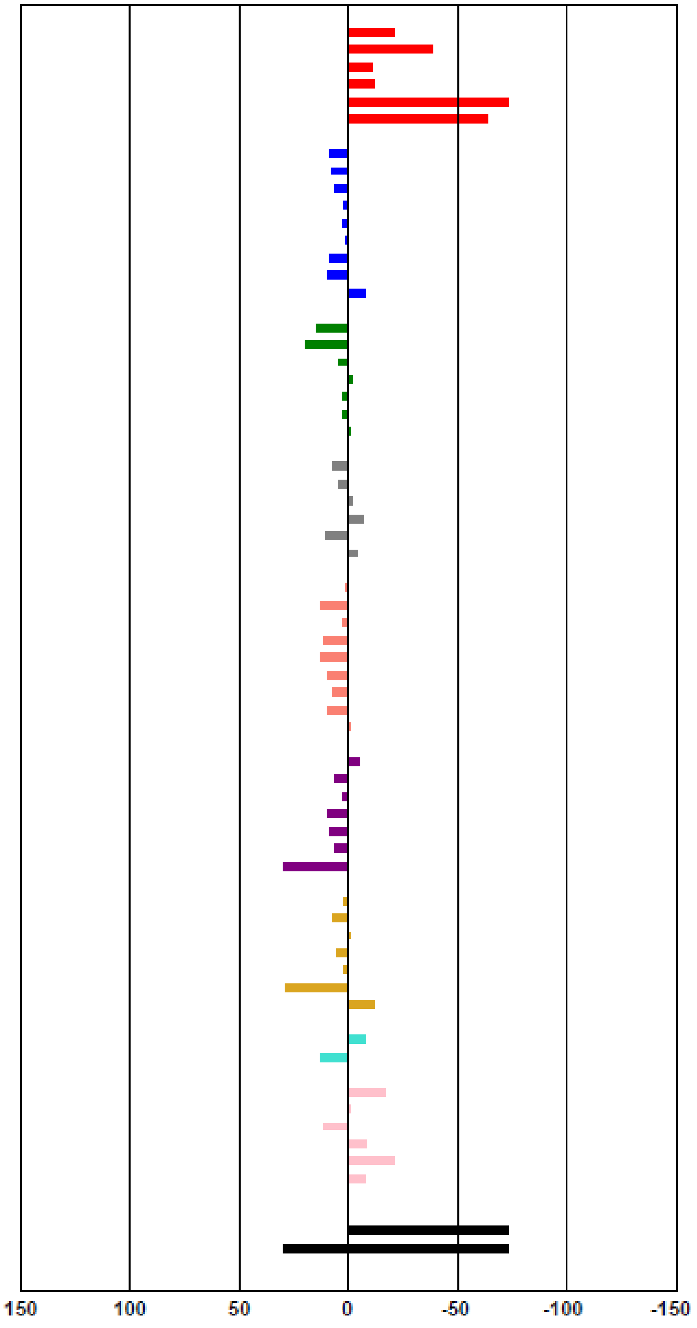

# Compound 3c

| Developmental Therapeutics Program One Dose Mean Graph | NSC: D-820122 / 1 | Conc: 1.00E-5 Molar | Test Date: Oct 28, 2019 |
|---|---|---|---|
| | Experiment ID: 1910OS87 | | Report Date: Nov 22, 2019 |

| Panel/Cell Line | Growth Percent |
|---|---|
| **Leukemia** | |
| CCRF-CEM | 3.83 |
| HL-60(TB) | -5.46 |
| K-562 | 18.87 |
| MOLT-4 | 1.09 |
| RPMI-8226 | -47.00 |
| SR | -14.70 |
| **Non-Small Cell Lung Cancer** | |
| A549/ATCC | 77.05 |
| EKVX | 77.70 |
| HOP-62 | 77.24 |
| HOP-92 | 75.50 |
| NCI-H226 | 64.80 |
| NCI-H23 | 77.36 |
| NCI-H322M | 93.30 |
| NCI-H460 | 93.71 |
| NCI-H522 | 15.94 |
| **Colon Cancer** | |
| COLO 205 | 72.69 |
| HCC-2998 | 79.51 |
| HCT-116 | 49.52 |
| HCT-15 | 27.84 |
| HT29 | 12.38 |
| KM12 | 65.10 |
| SW-620 | 18.17 |
| **CNS Cancer** | |
| SF-268 | 85.05 |
| SF-295 | 89.37 |
| SF-539 | 43.26 |
| SNB-19 | 56.92 |
| SNB-75 | 51.22 |
| U251 | 54.96 |
| **Melanoma** | |
| LOX IMVI | 6.21 |
| MALME-3M | 88.43 |
| M14 | 63.41 |
| MDA-MB-435 | 91.56 |
| SK-MEL-2 | 85.58 |
| SK-MEL-28 | 101.17 |
| SK-MEL-5 | 82.28 |
| UACC-257 | 80.98 |
| UACC-62 | 51.89 |
| **Ovarian Cancer** | |
| IGROV1 | 42.28 |
| OVCAR-3 | 20.29 |
| OVCAR-4 | 44.57 |
| OVCAR-5 | 65.35 |
| OVCAR-8 | 71.63 |
| NCI/ADR-RES | 70.39 |
| SK-OV-3 | 132.63 |
| **Renal Cancer** | |
| 786-0 | 49.55 |
| ACHN | 46.08 |
| CAKI-1 | 65.82 |
| RXF 393 | 15.57 |
| SN12C | 61.24 |
| TK-10 | 95.47 |
| UO-31 | 45.04 |
| **Prostate Cancer** | |
| PC-3 | 17.77 |
| DU-145 | 83.21 |
| **Breast Cancer** | |
| MCF7 | 32.74 |
| MDA-MB-231/ATCC | 56.84 |
| HS 578T | 87.42 |
| BT-549 | 10.32 |
| T-47D | -6.25 |
| MDA-MB-468 | 21.09 |
| Mean | 52.47 |
| Delta | 99.47 |
| Range | 179.63 |

Mean Growth Percent - Growth Percent

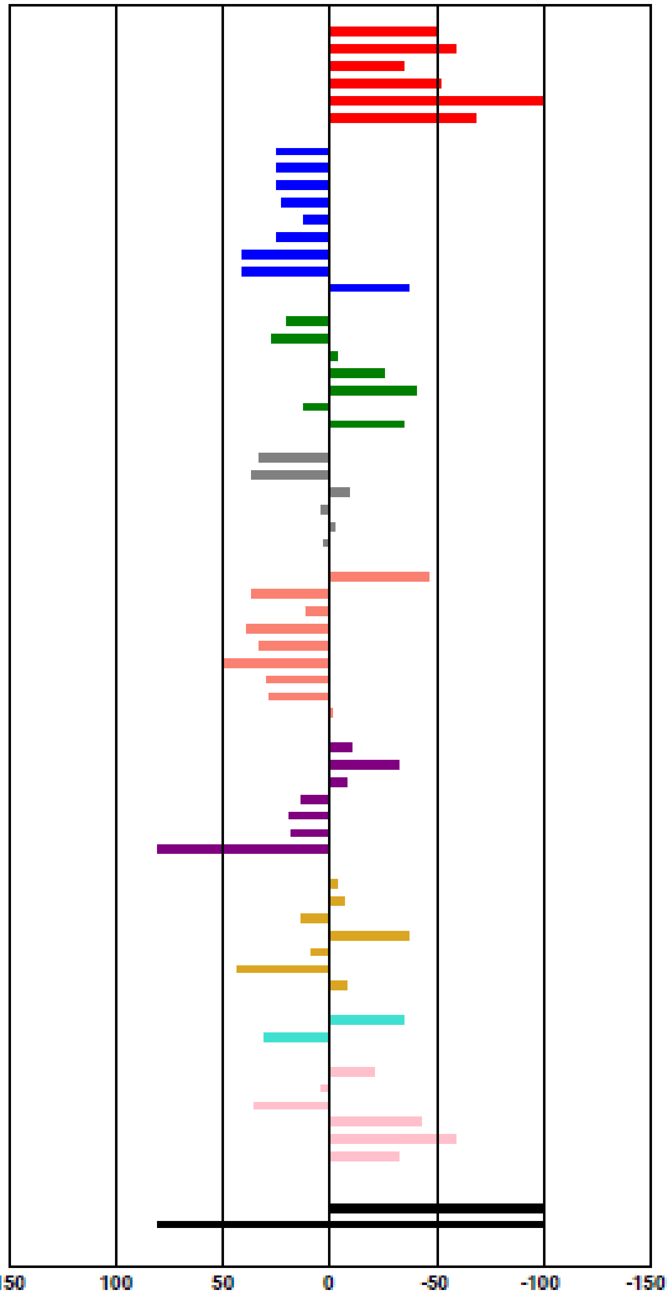

Compound 3d

| Developmental Therapeutics Program One Dose Mean Graph | NSC: D-820121 / 1 | Conc: 1.00E-5 Molar | Test Date: Oct 28, 2019 |
|---|---|---|---|
| | Experiment ID: 1910OS87 | | Report Date: Nov 22, 2019 |

| Panel/Cell Line | Growth Percent |
|---|---|
| Leukemia | |
| CCRF-CEM | 22.92 |
| HL-60(TB) | 91.84 |
| K-562 | 51.87 |
| MOLT-4 | 35.45 |
| RPMI-8226 | -9.36 |
| SR | 1.79 |
| Non-Small Cell Lung Cancer | |
| A549/ATCC | 94.80 |
| EKVX | 90.84 |
| HOP-62 | 106.61 |
| HOP-92 | 81.41 |
| NCI-H226 | 87.55 |
| NCI-H23 | 88.15 |
| NCI-H322M | 97.06 |
| NCI-H460 | 101.93 |
| NCI-H522 | 60.75 |
| Colon Cancer | |
| COLO 205 | 110.23 |
| HCC-2998 | 108.05 |
| HCT-116 | 53.98 |
| HCT-15 | 66.89 |
| HT29 | 46.01 |
| KM12 | 81.60 |
| SW-620 | 44.54 |
| CNS Cancer | |
| SF-268 | 103.02 |
| SF-295 | 93.58 |
| SF-539 | 99.67 |
| SNB-19 | 84.69 |
| SNB-75 | 78.08 |
| U251 | 81.83 |
| Melanoma | |
| LOX IMVI | 78.78 |
| MALME-3M | 92.44 |
| M14 | 78.86 |
| MDA-MB-435 | 100.35 |
| SK-MEL-2 | 97.30 |
| SK-MEL-28 | 102.07 |
| SK-MEL-5 | 92.47 |
| UACC-257 | 91.11 |
| UACC-62 | 77.16 |
| Ovarian Cancer | |
| IGROV1 | 89.24 |
| OVCAR-3 | 86.44 |
| OVCAR-4 | 97.07 |
| OVCAR-5 | 96.10 |
| OVCAR-8 | 88.22 |
| NCI/ADR-RES | 89.50 |
| SK-OV-3 | 140.00 |
| Renal Cancer | |
| 786-0 | 91.23 |
| ACHN | 87.90 |
| CAKI-1 | 87.79 |
| RXF 393 | 94.77 |
| SN12C | 86.86 |
| TK-10 | 107.62 |
| UO-31 | 75.38 |
| Prostate Cancer | |
| PC-3 | 48.38 |
| DU-145 | 105.72 |
| Breast Cancer | |
| MCF7 | 51.53 |
| MDA-MB-231/ATCC | 97.73 |
| HS 578T | 93.59 |
| BT-549 | 61.72 |
| T-47D | 62.75 |
| MDA-MB-468 | 76.32 |
| Mean | 81.05 |
| Delta | 90.41 |
| Range | 149.36 |

Mean Growth Percent - Growth Percent

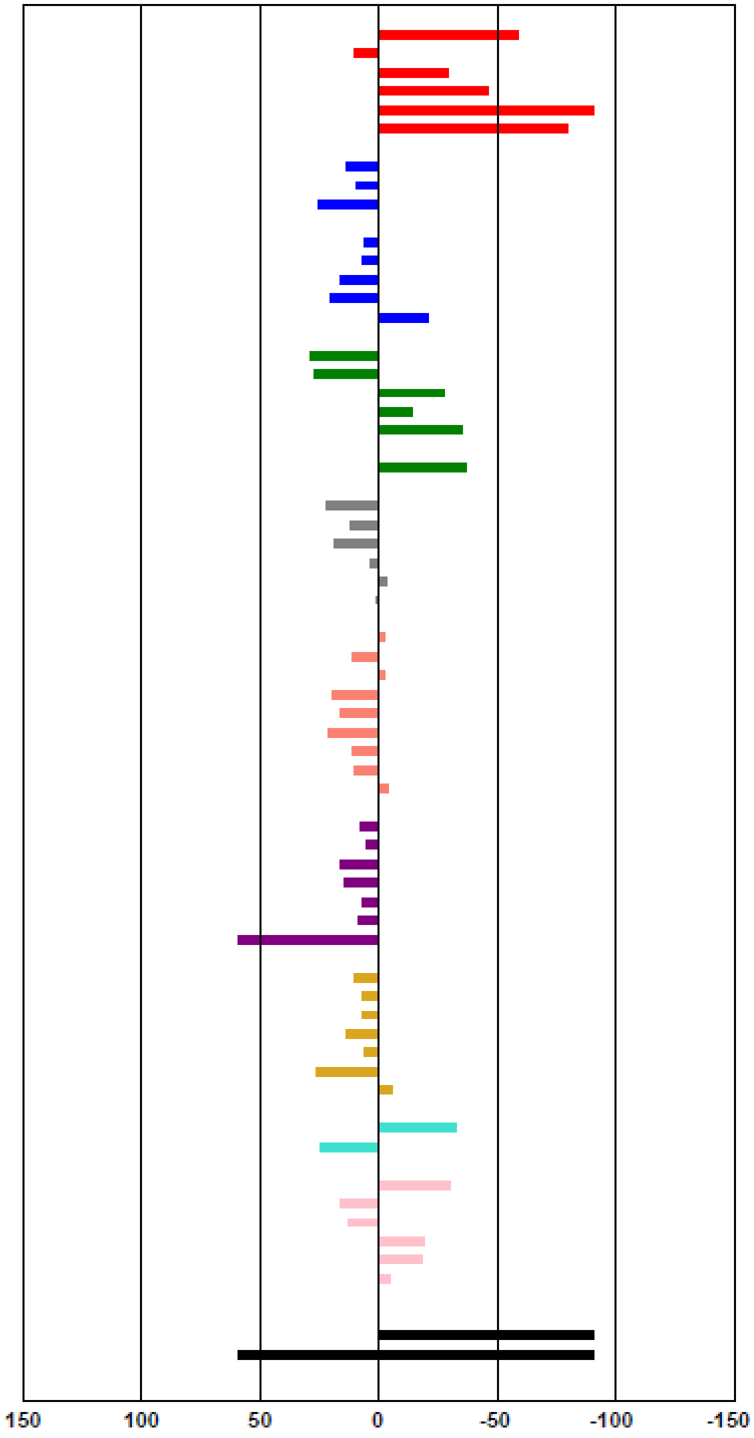

# Compound 3e

**Developmental Therapeutics Program**

**One Dose Mean Graph**

| NSC: D-820120 / 1 | Conc: 1.00E-5 Molar | Test Date: Oct 28, 2019 |
|---|---|---|
| Experiment ID: 1910OS87 | | Report Date: Nov 22, 2019 |

| Panel/Cell Line | Growth Percent |
|---|---|
| **Leukemia** | |
| CCRF-CEM | 33.92 |
| HL-60(TB) | 88.36 |
| K-562 | 54.22 |
| MOLT-4 | 37.15 |
| RPMI-8226 | -14.28 |
| SR | -13.40 |
| **Non-Small Cell Lung Cancer** | |
| A549/ATCC | 96.63 |
| EKVX | 93.56 |
| HOP-62 | 101.10 |
| HOP-92 | 81.57 |
| NCI-H226 | 92.69 |
| NCI-H23 | 99.60 |
| NCI-H322M | 92.25 |
| NCI-H460 | 104.24 |
| NCI-H522 | 59.69 |
| **Colon Cancer** | |
| COLO 205 | 108.10 |
| HCC-2998 | 105.43 |
| HCT-116 | 49.46 |
| HCT-15 | 68.40 |
| HT29 | 45.24 |
| KM12 | 84.99 |
| SW-620 | 8.79 |
| **CNS Cancer** | |
| SF-268 | 97.82 |
| SF-295 | 96.87 |
| SF-539 | 90.18 |
| SNB-19 | 85.57 |
| SNB-75 | 78.82 |
| U251 | 84.36 |
| **Melanoma** | |
| LOX IMVI | 73.49 |
| MALME-3M | 91.94 |
| M14 | 87.15 |
| MDA-MB-435 | 99.28 |
| SK-MEL-2 | 94.22 |
| SK-MEL-28 | 100.48 |
| SK-MEL-5 | 92.50 |
| UACC-257 | 92.74 |
| UACC-62 | 78.61 |
| **Ovarian Cancer** | |
| IGROV1 | 88.38 |
| OVCAR-3 | 92.91 |
| OVCAR-4 | 89.63 |
| OVCAR-5 | 100.40 |
| OVCAR-8 | 92.58 |
| NCI/ADR-RES | 86.54 |
| SK-OV-3 | 124.51 |
| **Renal Cancer** | |
| 786-0 | 90.55 |
| ACHN | 93.37 |
| CAKI-1 | 89.55 |
| RXF 393 | 100.53 |
| SN12C | 95.03 |
| TK-10 | 93.50 |
| UO-31 | 78.97 |
| **Prostate Cancer** | |
| PC-3 | 56.72 |
| DU-145 | 108.37 |
| **Breast Cancer** | |
| MCF7 | 43.71 |
| MDA-MB-231/ATCC | 96.05 |
| HS 578T | 98.97 |
| BT-549 | 92.27 |
| T-47D | 94.23 |
| MDA-MB-468 | 87.26 |
| Mean | 81.62 |
| Delta | 95.90 |
| Range | 138.79 |

Mean Growth Percent - Growth Percent

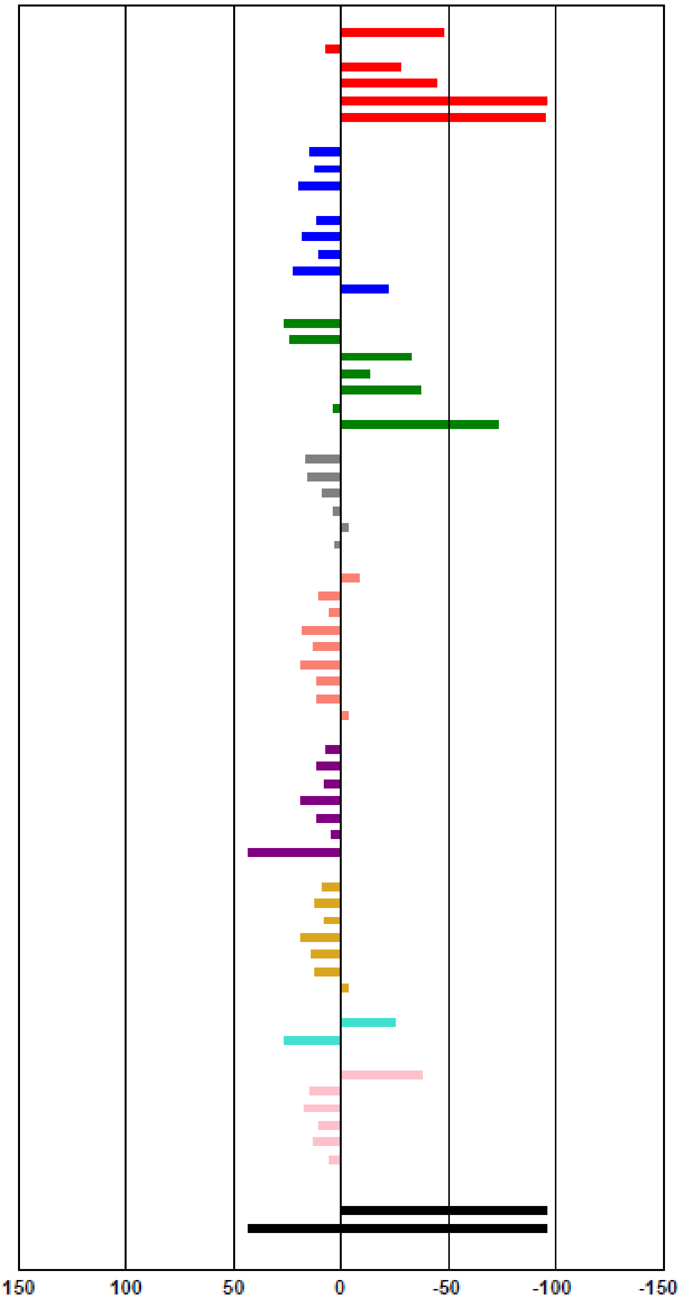

# Compound 3f

| Developmental Therapeutics Program One Dose Mean Graph | NSC: D-820117 / 1 | Conc: 1.00E-5 Molar | Test Date: Oct 28, 2019 |
|---|---|---|---|
| | Experiment ID: 1910OS87 | | Report Date: Nov 22, 2019 |

| Panel/Cell Line | Growth Percent |
|---|---|
| Leukemia | |
| CCRF-CEM | 58.57 |
| HL-60(TB) | 73.62 |
| K-562 | 69.48 |
| MOLT-4 | 44.39 |
| RPMI-8226 | 50.17 |
| SR | 60.87 |
| Non-Small Cell Lung Cancer | |
| A549/ATCC | 88.61 |
| EKVX | 87.84 |
| HOP-62 | 122.23 |
| HOP-92 | 83.84 |
| NCI-H226 | 90.59 |
| NCI-H23 | 90.68 |
| NCI-H322M | 95.57 |
| NCI-H460 | 98.02 |
| NCI-H522 | 69.99 |
| Colon Cancer | |
| COLO 205 | 95.52 |
| HCC-2998 | 108.91 |
| HCT-116 | 75.41 |
| HCT-15 | 70.57 |
| HT29 | 76.01 |
| KM12 | 98.68 |
| SW-620 | 87.06 |
| CNS Cancer | |
| SF-268 | 97.44 |
| SF-295 | 91.20 |
| SF-539 | 113.14 |
| SNB-19 | 88.74 |
| SNB-75 | 75.27 |
| U251 | 79.73 |
| Melanoma | |
| LOX IMVI | 90.59 |
| MALME-3M | 88.72 |
| M14 | 80.77 |
| MDA-MB-435 | 91.94 |
| SK-MEL-2 | 86.47 |
| SK-MEL-28 | 89.72 |
| SK-MEL-5 | 85.50 |
| UACC-257 | 83.67 |
| UACC-62 | 82.03 |
| Ovarian Cancer | |
| IGROV1 | 92.80 |
| OVCAR-3 | 84.13 |
| OVCAR-4 | 89.49 |
| OVCAR-5 | 102.27 |
| OVCAR-8 | 86.72 |
| NCI/ADR-RES | 92.28 |
| SK-OV-3 | 140.41 |
| Renal Cancer | |
| 786-0 | 85.38 |
| ACHN | 96.61 |
| CAKI-1 | 82.22 |
| RXF 393 | 75.51 |
| SN12C | 82.93 |
| TK-10 | 102.44 |
| UO-31 | 78.27 |
| Prostate Cancer | |
| PC-3 | 81.05 |
| DU-145 | 93.80 |
| Breast Cancer | |
| MCF7 | 63.62 |
| MDA-MB-231/ATCC | 96.50 |
| HS 578T | 94.99 |
| BT-549 | 73.19 |
| T-47D | 62.77 |
| MDA-MB-468 | 70.26 |
| Mean | 85.58 |
| Delta | 41.19 |
| Range | 96.02 |

Mean Growth Percent - Growth Percent

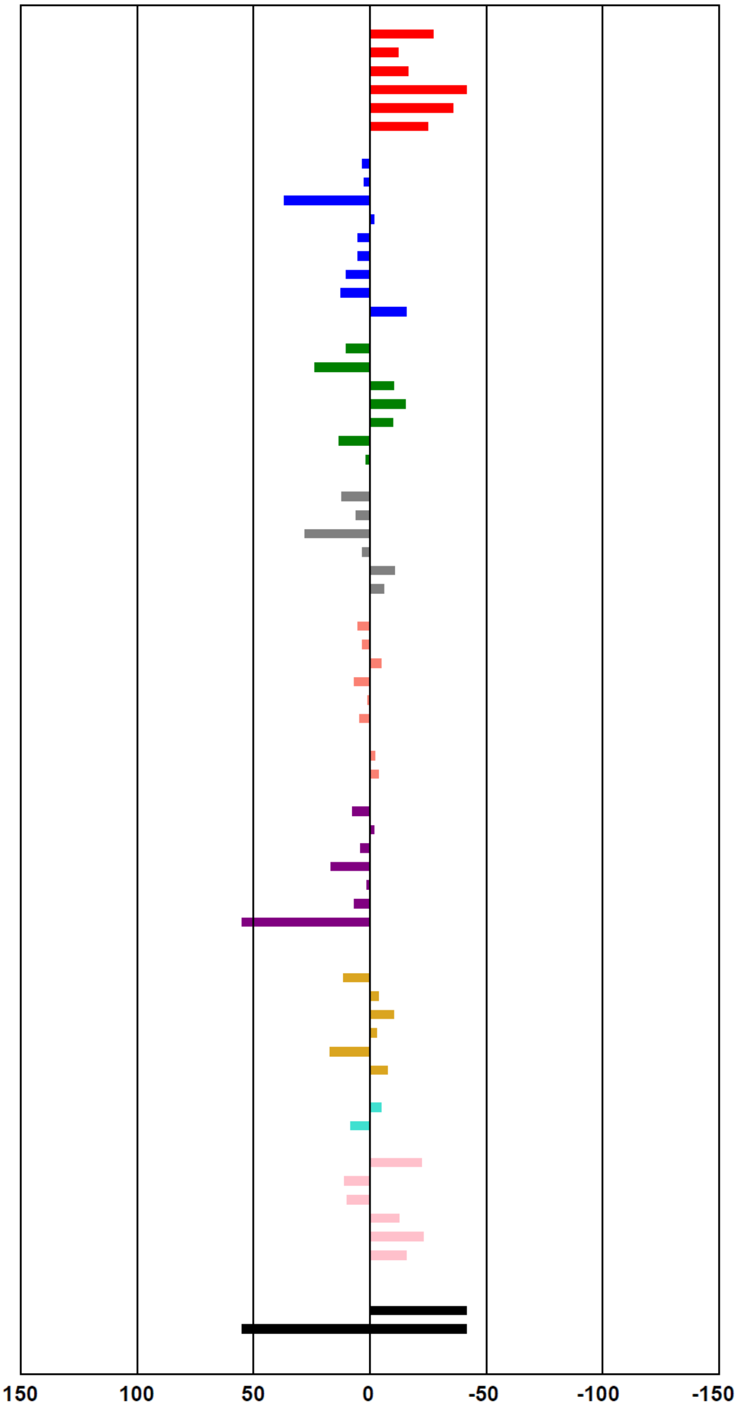

# Five dose full NCI 60 cell panel assay

National Cancer Institute Developmental Therapeutics Program
In-Vitro Testing Results

| | | | |
|---|---|---|---|
| NSC : D - 820119 / 1 | Experiment ID : 1912NS99 | Test Type : 08 | Units : Molar |
| Report Date : January 11, 2020 | Test Date : December 16, 2019 | QNS : | MC : |
| COMI : LE91 | Stain Reagent : SRB Dual-Pass Related | SSPL : 1B3N | |

| | | | Log10 Concentration | | | | | | | | | | | | | |
|---|---|---|---|---|---|---|---|---|---|---|---|---|---|---|---|---|
| | Time | | Mean Optical Densities | | | | | Percent Growth | | | | | | | | |
| Panel/Cell Line | Zero | Ctrl | -8.0 | -7.0 | -6.0 | -5.0 | -4.0 | -8.0 | -7.0 | -6.0 | -5.0 | -4.0 | GI50 | TGI | LC50 |
| Leukemia | | | | | | | | | | | | | | | |
| CCRF-CEM | 0.487 | 2.914 | 2.974 | 2.922 | 2.652 | 0.481 | 0.510 | 102 | 100 | 89 | -1 | 1 | 2.71E-6 | . | > 1.00E-4 |
| HL-60(TB) | 0.406 | 2.183 | 1.932 | 2.036 | 1.936 | 0.447 | 0.457 | 86 | 92 | 86 | 2 | 3 | 2.70E-6 | > 1.00E-4 | > 1.00E-4 |
| K-562 | 0.188 | 2.367 | 2.372 | 2.130 | 2.210 | 0.338 | 0.320 | 100 | 89 | 93 | 7 | 6 | 3.15E-6 | > 1.00E-4 | > 1.00E-4 |
| MOLT-4 | 0.547 | 2.770 | 2.824 | 2.852 | 2.657 | 0.636 | 0.608 | 102 | 104 | 95 | 4 | 3 | 3.12E-6 | > 1.00E-4 | > 1.00E-4 |
| RPMI-8226 | 0.785 | 2.894 | 2.888 | 2.888 | 2.671 | 0.558 | 0.588 | 100 | 100 | 89 | -29 | -25 | 2.15E-6 | 5.69E-6 | > 1.00E-4 |
| SR | 0.274 | 1.603 | 1.443 | 1.460 | 1.338 | 0.316 | 0.316 | 88 | 89 | 80 | 3 | 3 | 2.46E-6 | > 1.00E-4 | > 1.00E-4 |
| Non-Small Cell Lung Cancer | | | | | | | | | | | | | | | |
| A549/ATCC | 0.451 | 2.507 | 2.425 | 2.330 | 2.466 | 0.414 | 0.288 | 96 | 91 | 98 | -8 | -36 | 2.83E-6 | 8.37E-6 | > 1.00E-4 |
| EKVX | 0.739 | 2.159 | 2.111 | 2.027 | 2.028 | 0.340 | 0.090 | 97 | 91 | 91 | -54 | -88 | 1.91E-6 | 4.23E-6 | 9.38E-6 |
| HOP-62 | 0.765 | 2.634 | 2.633 | 2.553 | 2.663 | 0.621 | 0.330 | 100 | 96 | 102 | -19 | -57 | 2.68E-6 | 6.97E-6 | 6.60E-5 |
| HOP-92 | 1.240 | 1.907 | 1.803 | 1.754 | 1.712 | 1.205 | 0.747 | 84 | 77 | 71 | -3 | -40 | 1.91E-6 | 9.14E-6 | > 1.00E-4 |
| NCI-H226 | 0.851 | 1.538 | 1.487 | 1.519 | 1.505 | 1.021 | 0.545 | 93 | 97 | 95 | 25 | -36 | 4.38E-6 | 2.55E-5 | > 1.00E-4 |
| NCI-H23 | 0.613 | 1.933 | 1.880 | 1.862 | 1.856 | 0.257 | 0.197 | 96 | 95 | 94 | -58 | -68 | 1.95E-6 | 4.15E-6 | 8.85E-6 |
| NCI-H322M | 0.736 | 2.158 | 2.073 | 2.021 | 2.030 | 0.416 | 0.024 | 94 | 90 | 91 | -44 | -97 | 2.02E-6 | 4.75E-6 | 1.32E-5 |
| NCI-H460 | 0.403 | 3.139 | 3.175 | 3.169 | 3.162 | 0.167 | 0.115 | 101 | 101 | 101 | -59 | -72 | 2.08E-6 | 4.29E-6 | 8.82E-6 |
| NCI-H522 | 1.646 | 3.296 | 3.263 | 3.254 | 3.219 | 2.135 | 1.683 | 98 | 97 | 95 | 30 | 2 | 4.89E-6 | > 1.00E-4 | > 1.00E-4 |
| Colon Cancer | | | | | | | | | | | | | | | |
| COLO 205 | 0.438 | 1.938 | 2.017 | 1.890 | 1.924 | 0.219 | 0.236 | 105 | 97 | 99 | -50 | -46 | 2.13E-6 | 4.61E-6 | . |
| HCC-2998 | 0.812 | 2.712 | 2.557 | 2.559 | 2.594 | 0.158 | 0.088 | 92 | 92 | 94 | -81 | -89 | 1.78E-6 | 3.45E-6 | 6.68E-6 |
| HCT-116 | 0.279 | 2.366 | 2.314 | 2.208 | 2.166 | 0.084 | 0.030 | 98 | 92 | 90 | -70 | -89 | 1.79E-6 | 3.66E-6 | 7.51E-6 |
| HCT-15 | 0.280 | 1.739 | 1.718 | 1.648 | 1.601 | 0.116 | 0.092 | 99 | 94 | 91 | -59 | -67 | 1.87E-6 | 4.05E-6 | 8.76E-6 |
| HT29 | 0.402 | 2.477 | 2.462 | 2.389 | 2.357 | 0.159 | 0.109 | 99 | 96 | 94 | -60 | -73 | 1.93E-6 | 4.07E-6 | 8.56E-6 |
| KM12 | 0.607 | 3.019 | 3.088 | 3.020 | 2.999 | 0.236 | 0.196 | 103 | 100 | 99 | -61 | -68 | 2.03E-6 | 4.15E-6 | 8.51E-6 |
| SW-620 | 0.319 | 2.444 | 2.426 | 2.423 | 2.344 | 0.084 | 0.062 | 99 | 99 | 95 | -74 | -81 | 1.85E-6 | 3.66E-6 | 7.23E-6 |
| CNS Cancer | | | | | | | | | | | | | | | |
| SF-268 | 0.931 | 2.967 | 2.884 | 2.884 | 2.898 | 0.961 | 0.681 | 96 | 96 | 97 | 1 | -27 | 3.09E-6 | 1.12E-5 | > 1.00E-4 |
| SF-295 | 0.443 | 2.128 | 2.153 | 2.009 | 2.153 | 0.171 | 0.053 | 102 | 93 | 101 | -62 | -88 | 2.07E-6 | 4.19E-6 | 8.50E-6 |
| SF-539 | 0.878 | 2.905 | 2.811 | 2.817 | 2.840 | 0.099 | 0.009 | 95 | 96 | 97 | -89 | -99 | 1.79E-6 | 3.32E-6 | 6.18E-6 |
| SNB-19 | 0.591 | 2.168 | 2.040 | 2.056 | 2.057 | 0.267 | 0.049 | 92 | 93 | 93 | -55 | -92 | 1.95E-6 | 4.25E-6 | 9.26E-6 |
| SNB-75 | 2.041 | 3.178 | 2.993 | 3.059 | 2.976 | 2.478 | 2.060 | 84 | 90 | 82 | 38 | 2 | 5.44E-6 | > 1.00E-4 | > 1.00E-4 |
| U251 | 0.464 | 2.379 | 2.345 | 2.254 | 2.235 | 0.181 | 0.047 | 98 | 93 | 92 | -61 | -90 | 1.89E-6 | 4.00E-6 | 8.47E-6 |
| Melanoma | | | | | | | | | | | | | | | |
| LOX IMVI | 0.303 | 2.024 | 1.902 | 1.849 | 1.812 | 0.066 | 0.035 | 93 | 90 | 88 | -78 | -89 | 1.69E-6 | 3.37E-6 | 6.75E-6 |
| MALME-3M | 0.604 | 1.414 | 1.370 | 1.341 | 1.345 | 0.693 | 0.471 | 95 | 91 | 92 | 11 | -22 | 3.27E-6 | 2.14E-5 | > 1.00E-4 |
| M14 | 0.497 | 2.107 | 2.054 | 2.041 | 2.051 | 0.595 | 0.365 | 97 | 96 | 97 | 6 | -27 | 3.27E-6 | 1.54E-5 | > 1.00E-4 |
| MDA-MB-435 | 0.791 | 3.214 | 3.198 | 3.203 | 3.203 | 1.571 | 0.885 | 99 | 100 | 100 | 32 | 4 | 5.44E-6 | > 1.00E-4 | > 1.00E-4 |
| SK-MEL-2 | 1.053 | 2.159 | 2.074 | 2.034 | 2.108 | 0.929 | 0.578 | 92 | 89 | 95 | -12 | -45 | 2.65E-6 | 7.76E-6 | > 1.00E-4 |
| SK-MEL-28 | 0.642 | 2.000 | 1.975 | 1.966 | 1.936 | 0.639 | 0.355 | 98 | 98 | 95 | . | -45 | 2.97E-6 | 9.87E-6 | > 1.00E-4 |
| SK-MEL-5 | 0.996 | 3.323 | 3.299 | 3.288 | 3.305 | 2.002 | 1.342 | 99 | 98 | 99 | 43 | 15 | 7.56E-6 | > 1.00E-4 | > 1.00E-4 |
| UACC-257 | 1.150 | 2.795 | 2.749 | 2.635 | 2.642 | 1.701 | 1.581 | 97 | 90 | 91 | 33 | 26 | 5.14E-6 | > 1.00E-4 | > 1.00E-4 |
| UACC-62 | 0.905 | 3.021 | 2.934 | 2.863 | 2.896 | 0.392 | 0.247 | 96 | 93 | 94 | -57 | -73 | 1.96E-6 | 4.21E-6 | 9.02E-6 |
| Ovarian Cancer | | | | | | | | | | | | | | | |
| IGROV1 | 0.436 | 1.986 | 1.951 | 1.885 | 1.793 | 0.077 | 0.097 | 98 | 94 | 88 | -82 | -78 | 1.66E-6 | 3.27E-6 | 6.44E-6 |
| OVCAR-3 | 0.529 | 1.850 | 1.893 | 1.858 | 1.751 | 0.141 | 0.060 | 103 | 101 | 93 | -73 | -89 | 1.80E-6 | 3.61E-6 | 7.23E-6 |
| OVCAR-4 | 0.815 | 1.931 | 1.968 | 1.878 | 1.940 | 0.811 | 0.100 | 103 | 95 | 101 | . | -88 | 3.17E-6 | 9.88E-6 | 3.69E-5 |
| OVCAR-5 | 0.564 | 1.338 | 1.321 | 1.300 | 1.315 | 0.175 | 0.044 | 98 | 95 | 97 | -69 | -92 | 1.92E-6 | 3.84E-6 | 7.68E-6 |
| OVCAR-8 | 0.646 | 2.901 | 2.881 | 2.856 | 2.755 | 0.744 | 0.723 | 99 | 98 | 93 | 4 | 3 | 3.08E-6 | > 1.00E-4 | > 1.00E-4 |
| NCI/ADR-RES | 0.454 | 1.544 | 1.521 | 1.508 | 1.465 | 0.465 | 0.298 | 98 | 97 | 93 | 1 | -34 | 2.92E-6 | 1.06E-5 | > 1.00E-4 |
| SK-OV-3 | 0.661 | 1.881 | 1.748 | 1.777 | 1.865 | 0.786 | 0.652 | 89 | 91 | 99 | 10 | -1 | 3.55E-6 | 7.53E-5 | > 1.00E-4 |
| Renal Cancer | | | | | | | | | | | | | | | |
| 786-0 | 0.498 | 2.127 | 2.007 | 1.951 | 2.037 | 0.236 | 0.103 | 93 | 89 | 94 | -53 | -79 | 2.00E-6 | 4.38E-6 | 9.58E-6 |
| A498 | 1.944 | 2.497 | 2.192 | 2.275 | 2.261 | 1.850 | 1.639 | 45 | 60 | 57 | -5 | -16 | . | 8.36E-6 | > 1.00E-4 |
| ACHN | 0.301 | 1.222 | 1.198 | 1.198 | 1.195 | 0.029 | 0.044 | 97 | 97 | 97 | -91 | -85 | 1.78E-6 | 3.29E-6 | 6.08E-6 |
| CAKI-1 | 1.105 | 3.076 | 2.897 | 2.890 | 2.839 | 1.533 | 0.843 | 91 | 91 | 88 | 22 | -24 | 3.74E-6 | 3.00E-5 | > 1.00E-4 |
| RXF 393 | 1.595 | 2.363 | 2.305 | 2.264 | 2.153 | 0.778 | 0.285 | 92 | 87 | 73 | -51 | -82 | 1.52E-6 | 3.86E-6 | 9.78E-6 |
| SN12C | 0.525 | 2.199 | 2.104 | 2.104 | 2.072 | 0.383 | 0.266 | 94 | 94 | 92 | -27 | -49 | 2.27E-6 | 5.94E-6 | > 1.00E-4 |
| TK-10 | 1.057 | 2.023 | 1.854 | 1.752 | 1.877 | 0.755 | 0.269 | 83 | 72 | 85 | -29 | -75 | 2.03E-6 | 5.60E-6 | 2.92E-5 |
| UO-31 | 0.658 | 1.965 | 1.797 | 1.734 | 1.710 | 0.105 | 0.012 | 87 | 82 | 80 | -84 | -98 | 1.53E-6 | 3.08E-6 | 6.20E-6 |
| Prostate Cancer | | | | | | | | | | | | | | | |
| PC-3 | 0.525 | 2.132 | 2.058 | 2.094 | 1.903 | 0.172 | 0.127 | 95 | 98 | 86 | -67 | -76 | 1.71E-6 | 3.63E-6 | 7.70E-6 |
| DU-145 | 0.888 | 3.006 | 3.007 | 2.969 | 2.981 | 1.522 | 0.669 | 100 | 98 | 99 | 30 | -25 | 5.11E-6 | 3.53E-5 | > 1.00E-4 |
| Breast Cancer | | | | | | | | | | | | | | | |
| MCF7 | 0.517 | 2.575 | 2.438 | 2.389 | 2.203 | 0.331 | 0.126 | 93 | 91 | 82 | -36 | -76 | 1.87E-6 | 4.95E-6 | 2.25E-5 |
| MDA-MB-231/ATCC | 0.686 | 1.678 | 1.624 | 1.610 | 1.643 | 0.590 | 0.573 | 95 | 93 | 96 | -14 | -16 | 2.63E-6 | 7.46E-6 | > 1.00E-4 |
| HS 578T | 1.444 | 2.516 | 2.418 | 2.470 | 2.415 | 1.613 | 1.554 | 91 | 96 | 91 | 16 | 10 | 3.49E-6 | > 1.00E-4 | > 1.00E-4 |
| BT-549 | 0.928 | 2.081 | 2.007 | 1.941 | 2.028 | 0.598 | 0.257 | 94 | 88 | 95 | -36 | -72 | 2.22E-6 | 5.35E-6 | 2.47E-5 |
| T-47D | 0.994 | 2.332 | 2.241 | 2.147 | 1.996 | 0.416 | 0.437 | 93 | 86 | 75 | -58 | -56 | 1.54E-6 | 3.65E-6 | 8.68E-6 |
| MDA-MB-468 | 0.779 | 1.544 | 1.527 | 1.429 | 1.489 | 0.240 | 0.138 | 98 | 85 | 93 | -69 | -82 | 1.84E-6 | 3.74E-6 | 7.61E-6 |

**mitochondrial membrane potential assay for compound 3a, colchicine against control**

| Sample | | Results | |
|---|---|---|---|
| | | mitochondrial membrane potential | |
| s | Cpd.code | ΔΨm<br>**%**<br>**Apoptic cells** | **%**<br>**Live cells** |
| 1 | 3a/ **SR** | 31.19904 | 68.80096 |
| 2 | Colchicine/**SR** | 34.964784 | 65.03522 |
| 3 | cont.**SR** | 0.586224 | 99.41378 |

in vitro tubulin polymerization inhibitory activity

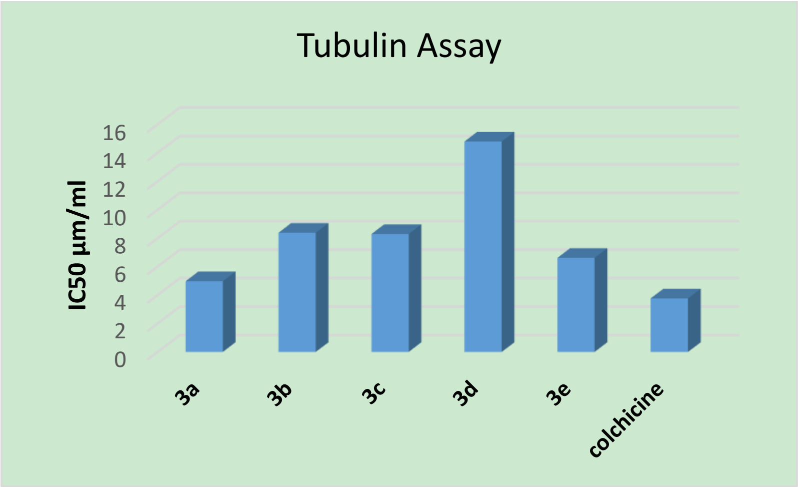

***Material and methods***

1. *NCI screening assay*

As mentioned, the methodology of the NCI procedure for primary anticancer assay was detailed on their site (http://www.dtp.nci.nih.gov). But briefly, the protocol performed at sixty human tumor cell lines panel derived from different nine neoplastic diseases. NCI-60 testing is performed in two parts: first, a single concentration is tested in all 60 cell lines at a single dose of 10-5 molar or 15 μg/mL in accordance with the protocol of the Drug Evaluation Branch, National Cancer Institute, Bethesda, USA. If the results obtained meet selection criteria, then the compound is tested again in all 60 cell lines in 5 x 10 folds of dilution with the top dose being 10-4 molar or 150 μg/mL. Detailed methods are described in supplementary material related to this article.

2. *MTT- Cytotoxicity assay method*

The MTT method of monitoring in vitro cytotoxicity is well suited for use with multi well plates. For best results, cells in the log phase of growth should be employed and final cell number should not exceed 106 cells/cm$^2$. Each test should include a blank containing complete medium without cells.

1. Remove cultures from incubator into laminar flow hood or other sterile work area.
2. Reconstitute each vial of MTT [M-5655] to be used with 3 ml of medium or balanced salt solution without phenol red and serum. Add reconstituted MTT in an amount equal to 10% of the culture medium volume.
3. Return cultures to incubator for 2-4 h depending on cell type and maximum cell density. (An incubation period of 2 h is generally adequate but may be lengthened for low cell

densities or cells with lower metabolic activity.) Incubation times should be consistent when making comparisons.

4. After the incubation period, remove cultures from incubator and dissolve the resulting formazan crystals by adding an amount of MTT Solubilization Solution [M-8910] equal to the original culture medium volume.

5. Gentle mixing in a gyratory shaker will enhance dissolution. Occasionally, especially in dense cultures, pipetting up and down [trituration] may be required to completely dissolve the MTT formazan crystals.

6. Spectrophotometrically measure absorbance at a wavelength of 570 nm. Measure the background absorbance of multi-well plates at 690 nm and subtract from the 450 nm measurement. Tests performed in multi-well plates can be read using the appropriate type of plate reader or the contents of individual wells may be transferred to appropriate size cuvets for spectrophotometric measurement.

*3. Analysis of cell cycle by flow cytometry*

Cytometers are Becton Dickinson Immunocytometry Systems, Beckman/Coulter Inc., DACO/Cytomation, and PARTEC GmbH.

1. The software used to deconvolute the DNA content frequency histograms to estimate the proportions of cells in the respective phases of the cycle, is available from Phoenix Flow Systems and Verity Software House.

2. Centrifuge that can accommodate 5-mL tubes.

3. PI staining solution: 0.1% (v/v) Triton X-100, 10 mg/mL PI (Molecular Probes, Inc.) and 100 mg/mL of DNase-free RNase A in PBS.

4. PBS (phosphate buffered saline, e.g. Dulbecco PBS): 136.9 mM NaCl, 2.68 mM KCl, 8.1 mM $Na_2HPO_4$, 0.9 mM $CaCl_2$, 0.49 mM $MgCl_2$.
5. DAPI staining solution: 0.1% (v/v) Triton X-100 and 1 mg/mL DAPI (Molecular Probes, Inc.) in PBS.
6. Monoclonal or polyclonal antibodies (Abs) applicable to cell-cycle analysis, including cyclin Abs (provided, e.g., by DACO Corporation, Sigma Chemical Co., Upstate Biotechnology Incorporated, B.D. Biosciences/PharMingen, and Santa Cruz Biotechnology, Inc.).
7. Cell permeabilizing solution: 0.25% Triton X-100, 0.01% sodium azide in PBS.
8. Rinsing solution: 1% bovine serum albumin (BSA), 0.01% sodium azide in PBS.
9. DNA denaturation buffer: 0.1 mM Na-EDTA in 1 mM Nacacodylate; adjust pH to 6.0. To make 0.2 M stock solution of cacodylate buffer, dissolve 42.8 g $Na(CH_3)_2\ As_2\ 3H_2O$ in 100 mL $H_2O$, take 50 mL of this solution, add to it 29.6 mL of 0.2 M HCl, and adjust volume to 200 mL with $H_2O$.
10. Diluting buffer: 0.1% Triton X-100, 0.5% (w/v) BSA in PBS.
11. 0.2 M phosphate buffer, pH 7.4 (mixture of 81 vol of 0.2 M $Na_2HPO_4$ with 19 vol of 0.2 M $KH_2PO_4$).

*4. Tubulin polymerization inhibitory activity*

Tubulin polymerization inhibitory activity was measured using kits pre-coated with biotin conjugated antibody specific to TUBb that bound to TUBb provided after addition of samples or standards. Avidin protein conjugated to horseradish peroxidase (HRP) enzyme was provided to bind the biotin labeled antibody. This complex gave a characteristic color change upon substrate addition *via* HRP enzyme-substrate reaction. The color change was measured

spectrophotometrically at a wavelength of 450 nm ± 10 nm. The decrease of color intensity was measured as a sign for tubulin inhibition. The results were calculated as the concentration of TUBb available for antibody reaction, and the percent inhibition of TUBb was calculated for each sample as a percent of control. In vitro kinetics of microtubule assembly was measured using ELISA kit for TUBb (Cloud-Clone. Corp.) on SR cell line. Briefly, growing cells from Leukemia SR cell lines were trypsinized, counted and seeded at the appropriate densities into 96-well microtiter plates. Cells then were incubated in a humidified atmosphere at $^{o}$C for 24 h. The standards, the tested compounds, and the control colchicine were diluted to designated concentrations. On the 96-well microtiter plates standard or sample was added to each well in 100 mL and incubated at $^{o}$C for 2 h. The solution was aspirated, and 100 mL of prepared detection reagent A was added to each well. Incubation was done at $^{o}$C for 2 h. After washing 100 mL of prepared Detection Reagent B was added and incubation was continued at $^{o}$C for 30 min. Five washings were done, then 90 mL of 3,30,5,50-tetramethylbenzidine (TMB) substrate solution was added and incubated at $^{o}$C for 15-25 min. Stop solution was added in 50 mL. Optical density (O.D.) was measured at 450 nm using micro-plate reader (Spectromax Plus 96 well plate spectrophotometer).

Results for each compound were reported, at 10 mM concentration, as the percent inhibition of the treated cells compared to that of the untreated control cells.

5. *Multidrug resistance Assay*

Before adding to wells, equilibrate the SABC working solution and TMB substrate for at least 30 min at room temperature (32 ℃). When diluting samples and reagents, they must be mixed completely and evenly. It is recommended plotting a standard curve for each test.

1. Set standard, test sample and control (zero) wells on the pre-coated plate respectively, and then, record their positions. It is recommended measuring each standard and sample in duplicate. Wash plate 2 times before adding standard, sample and control (zero) wells!
2. Aliquot 0.1ml of 20 ng/mL,10 ng/mL, 5 ng/mL, 2.5 ng/mL, 1.25 ng/mL, 0.625 ng/mL, 0.312 ng/mL, standard solutions into the standard wells.
3. Add 0.1 mL of Sample / Standard dilution buffer into the control (zero) well.
4. Add 0.1 mL of properly diluted sample (Rat serum, plasma, tissue homogenates and other biological fluids.) into test sample wells. Seal the plate with a cover and incubate at 32 ℃ for 90 min.
5. Remove the cover and discard the plate content, clap the plate on the absorbent filter papers or other absorbent material. Do NOT let the wells completely dry at any time. Do Not Wash Plate!
6. Add 0.1 mL of Biotin-detection antibody working solution into the above wells (standard, test sample & zero wells). Add the solution at the bottom of each well without touching the side wall. Seal the plate with a cover and incubate at 37 ℃ for 60 min.
7. Remove the cover, and wash plate 3 times with Wash buffer. Add 0.1 ml of SABC working solution into each well, cover the plate and incubate at ℃ for 30 min. Remove the cover and wash plate 5 times with washed buffer, and each time let the wash buffer stay in the wells for 1-2 min.
8. Add 90 µL of TMB substrate into each well, cover the plate and incubate at ℃ in dark within 15-30 min. (Note: This incubation time is for reference use only, the optimal time should be determined by end user) and the shades of blue can be seen in the first 3-4 wells (with most concentrated Abcb1 standard solutions), the other wells show no obvious

color. Add 50 µL of Stop solution into each well and mix thoroughly. The color changes into yellow immediately.

9. Read the O.D. absorbance at 450 nm in a micro-plate reader immediately after adding the stop solution.

*6. Caspase Assay*

Caspase assay is performed according to the following procedures:

1. Allowing all reagents to reach room temperature before use. Gently mix all liquid reagents prior to use (Note: A standard curve must be run with each assay).
2. Determine the number of 8-well strips needed for the assay. Insert these in the frame(s) for current use. (Re-bag extra strips and frame. Store these in the refrigerator for future use).
3. Add 100 µL of the Standard Diluent Buffer to the zero standard wells. Well(s) reserved for chromogen blank should be left empty.
4. Add 100 µL of standards and controls or diluted samples to the appropriate microtiter wells. The sample dilution chosen should be optimized for each experimental system. Tap gently on side of plate to mix.
5. Cover wells with plate cover and incubate for 2 hours at room temperature.
6. Thoroughly aspirate or decant solution from wells and discard the liquid. Wash wells 4 times. See Directions for Washing.
7. Pipette 100 µL of Caspase-3 (Active) Detection Antibody solution into each well except the chromogen blank(s). Tap gently on the side of the plate to mix.
8. Cover plate with plate cover and incubate for 1 h at room temperature.
9. Thoroughly aspirate or decant solution from wells and discard the liquid.

10. Wash wells 4 times. See Directions for Washing.
11. Add 100 μL Anti-Rabbit IgG HRP Working Solution to each well except the chromogen blank(s). Prepare the working dilution as described in Preparing IgG HRP.
12. Cover wells with the plate cover and incubate for 30 minutes at room temperature.
13. Thoroughly aspirate or decant solution from wells and discard the liquid. Wash wells 4 times. See Directions for Washing.
14. Add 100 μL of Stabilized Chromogen to each well. The liquid in the wells will begin to turn blue.
15. Incubate for 30 min at room temperature and in the dark. Note: Do not cover the plate with aluminum foil or metalized mylar. The incubation time for chromogen substrate is often determined by the microtiter plate reader used. Many plate readers have the capacity to record a maximum optical density (O.D.) of 2.0. The O.D. values should be monitored and the substrate reaction stopped before the O.D. of the positive wells exceeds the limits of the instrument. The O.D. values at 450 nm can only be read after the Stop Solution has been added to each well. If using a reader that records only to 2.0 O.D., stopping the assay after 20 to 25 minutes is suggested.
16. Add 100 μL of Stop Solution to each well. Tap side of plate gently to mix. The solution in the wells should change from blue to yellow.
17. Read the absorbance of each well at 450 nm having blanked the plate reader against a chromogen blank composed of 100 μL each of Stabilized Chromogen and Stop Solution. Read the plate within 2 hours after adding the Stop Solution.
18. Use a curve fitting software to generate the standard curve. A four-parameter algorithm provides the best standard curve fit.

19. Read the concentrations for unknown samples and controls from the standard curve. Multiply value(s) obtained for sample(s) by the appropriate dilution factor to correct for the dilution in step 3. Samples producing signals greater than that of the highest standard should be diluted in Standard Diluent Buffer and reanalyzed.

*3.8* BAX assay

Bring all reagents, except the human Bax-α Standard, to room temperature for at least 30 minutes prior to opening.

The human Bax-α Standard solution should not be left at room temperature for more than 10 minutes.

All standards, controls and samples should be run in duplicate.

1. Refer to the Assay Layout Sheet to determine the number of wells to be used and put any remaining wells with the

desiccant back into the pouch and seal the ziploc. Store unused wells at 4 ℃.

2. Pipet 100 μL of Assay Buffer into the S0 (0 pg/mL standard) wells.
3. Pipet 100 μL of Standards #1 through #6 into the appropriate wells.
4. Pipet 100 μL of the Samples into the appropriate wells.
5. Tap the plate gently to mix the contents.
6. Seal the plate and incubate at room temperature on a plate shaker for 1 hour at ~500 rpm.
7. Empty the contents of the wells and wash by adding 400 μL of wash solution to every well.
8. Repeat the wash 4 more times for a total of 5 washes.
9. After the final wash, empty or aspirate the wells and firmly tap the plate on a lint free paper towel to remove any remaining wash buffer.
10. Pipet 100 μL of yellow Antibody into each well, except the Blank.
11. Seal the plate and incubate at room temperature on a plate shaker for 1 hour at ~500 rpm.
12. Empty the contents of the wells and wash by adding 400 μL of wash solution to every well.
13. Repeat the wash 4 more times for a total of 5 washes.
14. After the final wash, empty or aspirate the wells and firmly tap the plate on a lint free paper towel to remove any remaining wash buffer.

15. Add 100 µL of blue Conjugate to each well, except the Blank.
16. Seal the plate and incubate at room temperature on a plate shaker for 30 minutes at ~500 rpm.
17. Empty the contents of the wells and wash by adding 400 µL of wash solution to every well.
18. Repeat the wash 4 more times for a total of 5 washes.
19. After the final wash, empty or aspirate the wells and firmly tap the plate on a lint free paper towel to remove any remaining wash buffer.
20. Pipet 100 µL of Substrate Solution into each well.
21. Incubate for 30 minutes at room temperature on a plate shaker at ~500 rpm.
22. Pipet 100 µL Stop Solution to each well.
23. Blank the plate reader against the Blank wells, read the optical density at 450 nm, preferably with correction between 570 and 590 nm. If the plate reader is not able to be blanked against the Blank wells, manually subtract the mean optical density of the Blank wells from all the readings.

*3.9. BcL2 Assay*

Assay was performed as the following protocol

1. Mix all reagents thoroughly without foaming before use.
2. Wash the microwells twice with approximately 300 µL Wash Buffer per well with thorough aspiration of microwell contents between washes.
3. Take caution not to scratch the surface of the microwells.
4. After the last wash, empty the wells and tap microwell strips on absorbent pad or paper towel to remove excess Wash Buffer.
5. Use the microwell strips immediately after washing or place upside down on a wet absorbent paper for not longer than 15 minutes.
6. Do not allow wells to dry. Add 100 µL of Sample Diluent in duplicate to all standard wells and to the blank wells. Prepare standard (1:2 dilution) in duplicate ranging from 32 ng/mL to 0.5 ng/mLAdd 100 µL of Sample Diluent, in duplicate, to the blank wells. Add 80 µL of Sample Diluent, in duplicate, to the sample wells.

7. Add 20 µL of each Sample , in duplicate, to the designated wells.
8. Add 50 µL of diluted biotin-conjugate to all wells, including the blank wells.
9. Cover with a plate cover and incubate at room temperature, on a microplate shaker at 100 rpm if available, for 2 hours.
10. Remove plate cover and empty the wells. Wash microwell strips 3 times as described in step 2.
11. Add 100 µL of diluted Streptavidin-HRP to all wells, including the blank wells.
12. Cover with a plate cover and incubate at room temperature, on a microplate shaker at 100 rpm if available, for 1 hour.
13. Remove plate cover and empty the wells. Wash microwell strips 3 times as described in step 2. Proceed to the next step.
14. Pipette 100 µl of mixed TMB Substrate Solution to all wells, including the blanks.
15. Incubate the microwell strips at room temperature (18 °to 25 ℃) for about 15 minutes, if available on a rotator set at 100 rpm. Avoid direct exposure to intense light. The point, at which the substrate reaction is stopped, is often determined by the ELISA reader. Many ELISA readers record absorbance only up to 2.0 O.D. Therefore, the color development within individual microwells must be watched by the person running the assay and the substrate reaction stopped before positive wells are no longer properly detectable.
16. Stop the enzyme reaction by quickly pipetting 100 µL of Stop Solution into each well, including the blank wells. It is important that the Stop Solution is spreaded quickly and uniformly throughout the microwells to completely inactivate the enzyme. Results must be read immediately after the Stop Solution is added or within one hour if the microwell strips are stored at 2 - 8 ℃ in the dark.
17. Read absorbance of each microwell on a spectrophotometer using 450 nm as the primary wave length (optionally 620 nm as the reference wave length; 610 nm to 650 nm is acceptable).

*3.10 Assessment of Mitochondrial Changes*

*The assay was done as following*

**a.** Grow cells (adherent or suspension) in appropriate media to obtain at least of 3 x 104 cells per assayed conditions; positive, negative and experimental controls, and test compound(s). Ensure

that adherent cells are sub-confluent. Account for cell loss during the processing. Negative control – unlabeled cells not exposed to ROS Inducer or treatment, Positive control – cells incubated with 1X ROS Label only.

Experimental control – labeled cells treated with 1X ROS Inducer.

1. Harvest the suspension cells by centrifugation at 300 x g for 5 min at room temperature. Use these setting throughout the entire protocol for both cell types.
2. Fully detach adherent cells (e.g. trypsinize and quench with media) and harvest by centrifugation. Resuspend the cell pellets in culture media with 1X ROS Label.
3. Ensure a single cell suspension by gently pipetting up and down and incubate for 30 minutes at ℃ protected from light.
4. Upon completion, spin down the cells and remove the media. DO NOT wash the cells. Treat the cells with compound(s) of interest for desired time period directly in culture media, ROS Assay Buffer supplemented with 10% FBS, or culture media without phenol red. Include appropriate controls. If using ROS Inducer as an experimental control, dilute the stock to 1X and treat the cells for 1 hour prior to analyses.
5. Adjust the cell concentration so at least 1 x104 cells should be analyzed per experimental condition. Gently pipette cells up/down to ensure single cell suspension and analyze on flow cytometer in FL-1 channel. Establish forward and side scatter gates from negative control cells to exclude debris and cellular aggregates. Mean fluorescence intensity in Ex/Em = 495/529 nm can be quantified and compared between untreated cells and cells treated with test compounds, or between different cell types.

*3.11 Detection of ROS in Suspension and Adherent Cells by Microplate Assay:*

1. Seed 2.5 x 104 adherent cells per well in 96-well plate to obtain ~ 70-80% confluency on the day of experiment.
2. Allow cells to adhere overnight. Grow suspension cells so that approximately 1.5 x 105 cells per well are available. Next day, remove the media and wash the adherent cells in 100 µl of ROS Assay Buffer. Collect suspension cells by centrifugation and wash once in PBS. Discard the wash.
3. Add 100 µL of 1X ROS Label diluted in ROS Assay Buffer per well into adherent cells or re suspend the pelleted cells at 1.5 x 106 cells/mL. Incubate for 45 min at ℃ in the dark.
4. For adherent cells: remove the ROS Label, add 100 µl of ROS Assay Buffer or PBS and measure fluorescence immediately, or treat the cells with 100 µL of diluted test compound(s) for desired period of time. Include appropriate controls as well as blank wells (media or buffer only). For suspension cells: wash the cells by centrifugation in ROS Assay Buffer, maintain the same cell concentration.
5. Seed 100,000 labeled cells per well in 100 µl volume and measure the ROS or treat the cells with test compound(s) in ROS Assay Buffer supplemented with 10% FBS or media without phenol red for appropriate time. If using ROS Inducer as an **experimental** control, dilute the ROS inducer stock to 1X and treat the cells for 1 hour prior to analyses.
6. Measure fluorescence at Ex/Em= 495/529 nm in end point mode in presence of compounds and controls. Determine change in fluorescence after background subtraction.

*3.12. Docking studies*

Docking simulation study is performed using Molecular Operating Environment (MOE®) version 2014.09, Chemical Computing Group Inc., Montreal, Canada. The computational software operated under “Windows XP” installed on an Intel Pentium IV PC with a 1.6 GHz processor and 512 MB memory. The target compounds were constructed into a 3d model using the builder interface of the MOE program. After checking their structures and the formal charges on atoms by 2D depiction, the following steps were carried out:

- All conformers were subjected to energy minimization, all the minimizations were performed with MOE until a RMSD gradient of 0.01 Kcal/mole and RMS (RootMean Square) distance of 0.1 Å with MMFF94X force-field and the partial charges were automatically calculated.
- The obtained database was then saved as Molecular Data Base (MDB) file to be Used in the docking calculations.

*3.12.1. Optimization of the target*

The X-ray crystallographic structure of the target β-tubulin (PDB: 3HKC) obtained from Protein data bank. The compounds were docked on the active site the target enzyme.

The enzyme was prepared for docking studies by:

- The co-crystallized ligand was deleted.
- Hydrogen atoms were added to the system with their standard geometry.
- The atoms connection and type were checked for any errors with automatic correction.
- Selection of the receptor and its atoms potential were fixed.

*3.12.2. Docking of the target molecules to colchicine active site*

Docking of the target compounds was done using MOE-Dock software. The following methodology was generally applied:

- The enzyme active site file was loaded, and the Dock tool was initiated. The program specifications were adjusted to:

- Dummy atoms as the docking site.

- Triangle matcher as the placement methodology to be used.

- London dG as Scoring methodology to be used and was adjusted to its default values.

- The MDB file of the ligand to be docked was loaded and Dock calculations were run automatically.
- The obtained poses were studied, and the poses showed best ligand-enzyme interactions were selected and stored for energy calculations.
